# Supplementary material for: Improved polygenic prediction by Bayesian multiple regression on summary statistics
Source: Nat Commun. 2019 Nov 8;10:5086. doi: 10.1038/s41467-019-12653-0 (PMC6841727; doi:10.1038/s41467-019-12653-0)
Supplement: Supplementary file 1 — Supplementary Information [file 41467_2019_12653_MOESM1_ESM.pdf]

Supplementary information - Improved polygenic prediction by Bayesian multiple regression on summary statistics

Lloyd-Jones, Zeng et al.

Supplementary Figures

Supplementary Figure 1

Prediction accuracy (R^2) for SBayesR using different LD matrix reference cohorts in the simulation on chromosomes 21 and 22 6

Supplementary Figure 2

SNP-based heritability estimation (h^2_{SNP}) for SBayesR using different LD matrix reference cohorts in the simulation on chromosomes 21 and 22. . . . 7

Supplementary Figure 3

Prediction accuracy performance using different methods in the simulation on chromosomes 21 and 22. 8

Supplementary Figure 4

SNP-based heritability (h^2_{SNP}) estimation for different methods in the simulation on chromosomes 21 and 22. 9

Supplementary Figure 5

Slope estimates from regression of observed phenotypic values on the predicted values from SBayesR in genome-wide simulation. 10

Supplementary Figure 6

SNP-based heritability (h^2_{SNP}) estimation performance for different methods in UKB genome-wide simulation 11

Supplementary Figure 7

BayesR prediction accuracy and SNP-based heritability (h^2_{SNP}) estimation as a function of MCMC chain length for one scenario of UKB genome-wide simulation. 12

Supplementary Figure 8

SBayesR prediction accuracy and SNP-based heritability (h^2_{SNP}) estimation change with MCMC chain length for one scenario of UKB genome-wide simulation. 13

Supplementary Figure 9

Regression with Summary Statistics (RSS)[1] prediction accuracy for results generated from 200,000 (200k) and 2,000,000 (2M) iterations of the MCMC chain for all scenarios of the UKB genome-wide simulation. 14

Supplementary Figure 10

Regression with Summary Statistics (RSS)[1] SNP-based heritability (h^2_{SNP}) estimates for results generated from 200,000 (200k) and 2,000,000 (2M) iterations of the MCMC chain for all scenarios of the UKB genome-wide simulation. 15

Supplementary Figure 11

Runtime (log(hours)) comparison for BayesR, SBayesR, RSS, LDpred and SBLUP for UKB genome-wide simulation. 16

Supplementary Figure 12

Memory usage in gigabytes (GB) comparison for BayesR, SBayesR, RSS, LDpred and SBLUP for UKB genome-wide simulation. 17

Supplementary Figure 13

SBayesR prediction accuracy change as a function of number of mixtures fitted. 18

Supplementary Figure 14

SBayesR computational time change as a function of number of mixtures fitted. 19

Supplementary Figure 15

Slope estimates from regression of observed phenotypic values on the predicted values from SBayesR in UKB cross-validation. 20

Supplementary Figure 16

SNP-based heritability (h^2_{SNP}) estimation performance for different methods in the 5-fold cross-validation analysis of 12 quantitative traits in the UKB. . 21

Supplementary Figure 17

SNP-based heritability (h^2_{SNP}) point estimates and highest-probability densities for SBayesR in the 5-fold cross-validation analysis of 12 traits in the UKB. 22

Supplementary Figure 18

Runtime (log(hours)) comparison for BayesR, SBayesR, RSS, LDpred and SBLUP in cross-validation analysis of 12 traits in the UKB. 23

Supplementary Figure 19

Memory usage comparison in gigabytes (GB) for cross validation analysis of 10 quantitative traits in the UKB. 24

Supplementary Figure 20

Regression with Summary Statistics (RSS)[1] prediction accuracy for results generated from 200,000 (200k) and 2,000,000 (2M) iterations of the MCMC chain in the 5-fold cross-validation analysis of 10 quantitative traits in the UKB. 25

Supplementary Figure 21

Regression with Summary Statistics (RSS)[1] SNP-based heritability (h^2_{SNP}) estimates for results generated from 200,000 (200k) and 2,000,000 (2M) iterations of the MCMC chain in the 5-fold cross-validation analysis of 10 quantitative traits in the UKB. 26

Supplementary Figure 22

Variability in the number of per variant window width (measured in Mb) from the shrunk-sparse LD correlation matrix within chromosome for each of 1.09 million HapMap3 variants in the UKB. 27

Supplementary Figure 23

Distribution and truncation of per-variant sample size from BMI and height summary statistics for 982,000 HapMap3 variants from Yengo *et al.*[2] 28

Supplementary Tables

Supplementary Table 1

Summary of across-biobank predictions and testing of polygenic risk scores (PRSs) variance explained. 29

Supplementary Notes

1 Supplementary Note 1 - Simulation study using chromosomes 21 and 22 30

1.1 Description 30

1.2 Results 34

2 Supplementary Note 2 - Bayesian multiple regression 36

2.1 Joint sampling of δ_j and β_j 42

3 Supplementary Note 3 - Summary statistics based Bayesian multiple regression 48

3.1 Sampling β_j 50

3.2 Sampling σ_ε^2 52

3.3 Computing estimate of genotypic variance 54

4 Supplementary Note 4 - Method summary and implementation 55

5 Supplementary Note 5 - Full-data likelihood equivalence 58

6 Supplementary Note 5 - Additional acknowledgements 60

Supplementary References 63

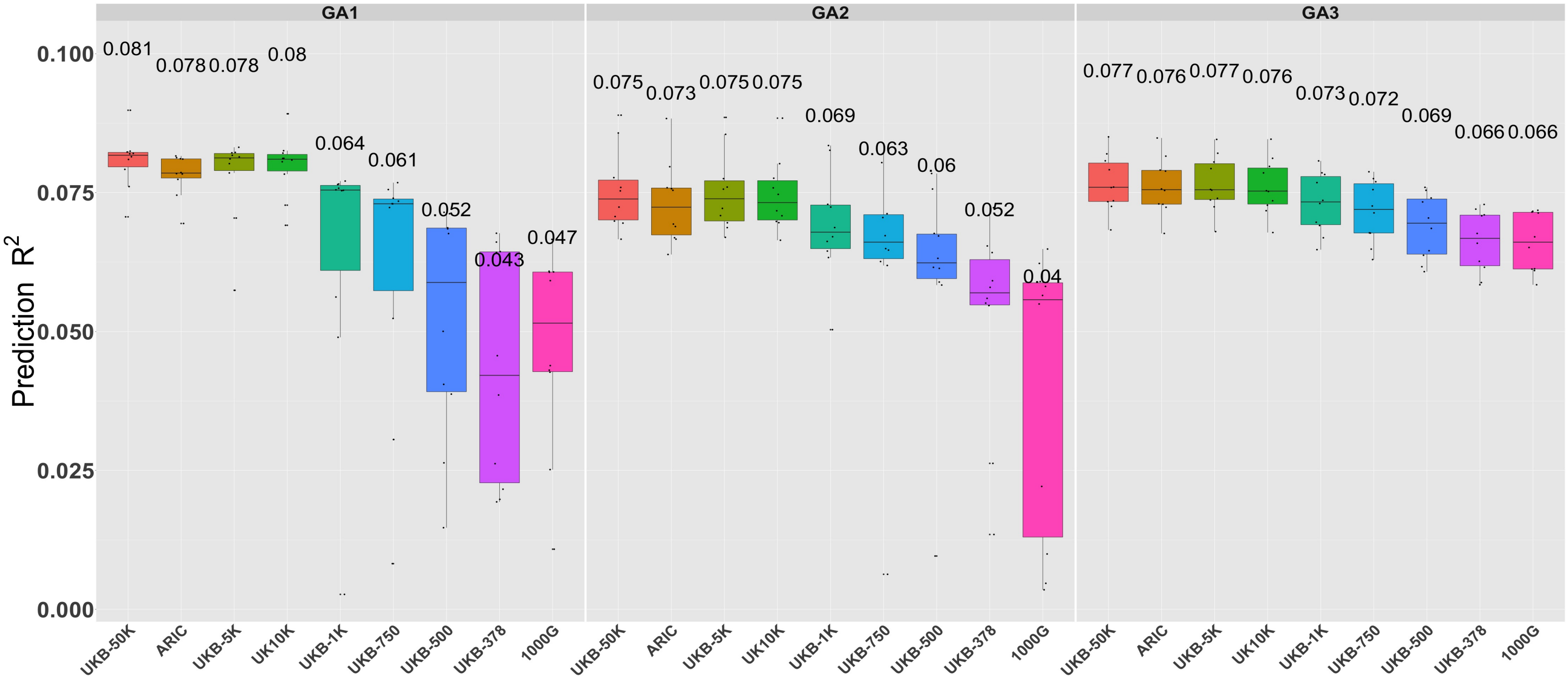

Supplementary Figure 1 Prediction accuracy (R^2) for SBayesR using different LD matrix reference cohorts in the simulation on chromosomes 21 and 22. Each panel displays boxplot summaries of the prediction R^2 (y-axis) from the SBayesR method in the 10,000 individual validation data set for each LD reference cohort (x-axis). Each boxplot summarises results from 10 replicates for each of the three simulation scenarios i.e., the first genetic architecture (GA1) contained two causal variants explaining 3% and 2% of the phenotypic variance and 1,498 causal variants sampled from a N(0, 0.05/1, 498). The second architecture (GA2) was simulated under a BayesR model with three sets of causal variants: the first with 1,445 causal variants sampled from a $N(0, 0.06/1445)$ distribution, the second 50 causal variants from $N(0, 0.02/50)$ and the third 5 causal variants $N(0, 0.02/5)$. The third architecture (GA3) contained 1,500 variants sampled from a $N(0, 0.1/1500)$ distribution. The mean R^2 across the 10 replicates is displayed above the boxplot for each cohort. Poorer prediction accuracies for the 1000G cohort are hypothesised to be primarily driven by the small sample size ($n = 378$) of this reference, with small references having a larger sampling variance for the "non-true" LD matrix entries, which can influence the convergence of the approximate SBayesR Gibbs sampling algorithm. All UKB cohorts are random sub samples from the UK Biobank unrelated individuals. Sample size for Atherosclerosis Risk in Communities (ARIC)[3] and GENEVA Diabetes study was 12,942, Phase 3 of the 1000 Genomes Project (1000G)[4] contained 378 individuals with and the UK10K project[5] 3,642 individuals. The box plot centre line is the median, the bottom and top of the box are the first (Q1) and third quartiles (Q3) and the lower and upper whiskers are Q1 – 1.5 IQR and Q3 + 1.5 IQR, respectively, where IQR = Q3 – Q1. The points depict the prediction R^2 for each replicate.

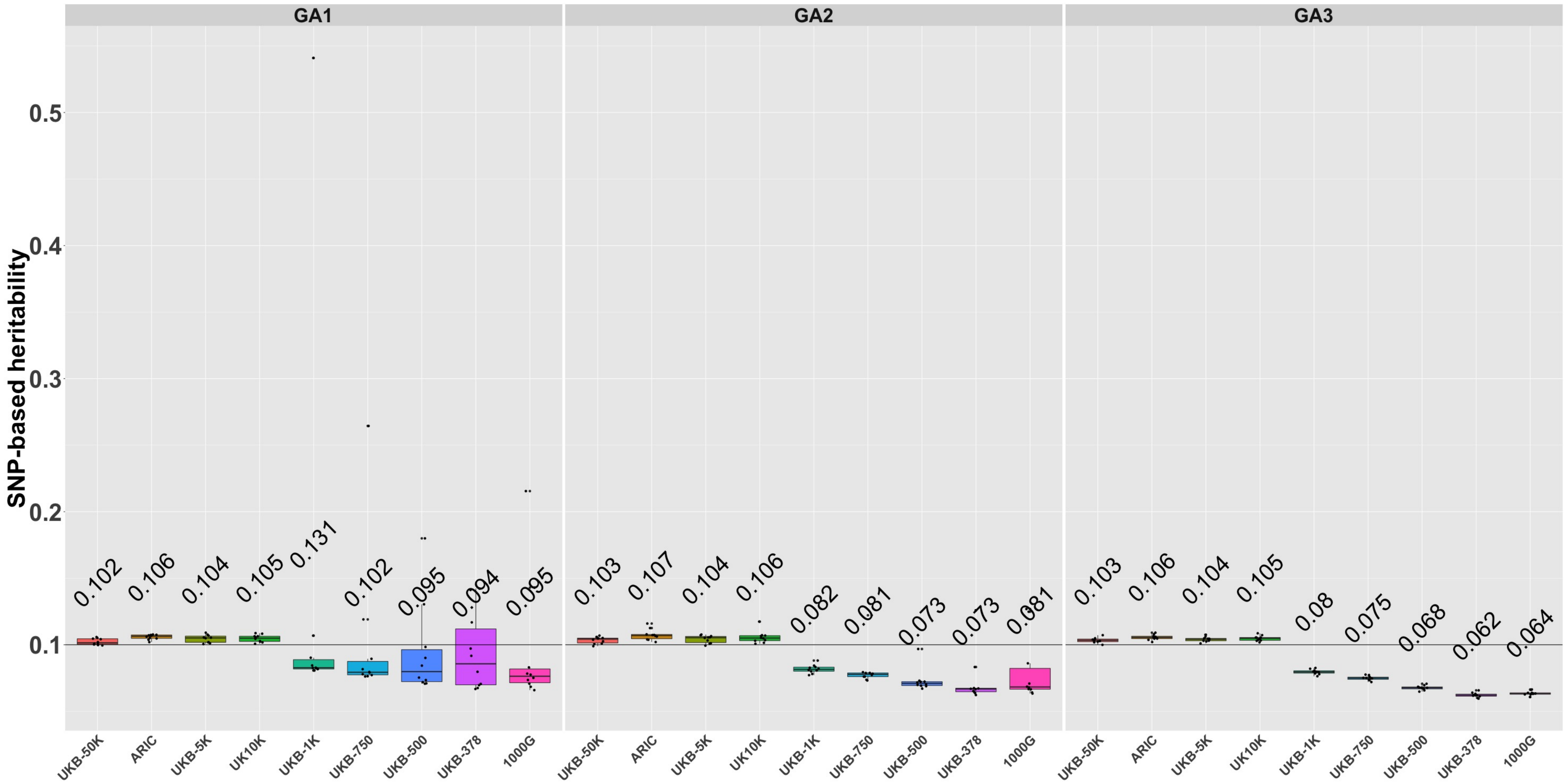

Supplementary Figure 2 SNP-based heritability estimation (h^2_{SNP}) for SBayesR using different LD matrix reference cohorts in the simulation on chromosomes 21 and 22. Each panel displays boxplot summaries of h^2_{SNP} estimates (y-axis) for each LD reference cohort (x-axis) across the 10 replicates for each of the three simulation scenarios in the chromosome 21 and 22 simulation. Each trait has a simulated true $h^2_{SNP} = 0.1$ (horizontal line) and 1,500 causal variants. The mean h^2_{SNP} across the 10 replicates is displayed above the boxplot for each cohort. Inflated h^2_{SNP} estimates for the 1000G cohort are hypothesised to be primarily driven by the small sample size ($n = 378$) of this reference. See Supplementary Figure 1 for descriptions of the genetic architectures (GA1, GA2, GA3).The box plot centre line is the median, the bottom and top of the box are the first (Q1) and third quartiles (Q3) and the lower and upper whiskers are Q1 – 1.5 IQR and Q3 + 1.5 IQR, respectively, where IQR = Q3 – Q1. The points depict the h^2_{SNP} estimate for each replicate.

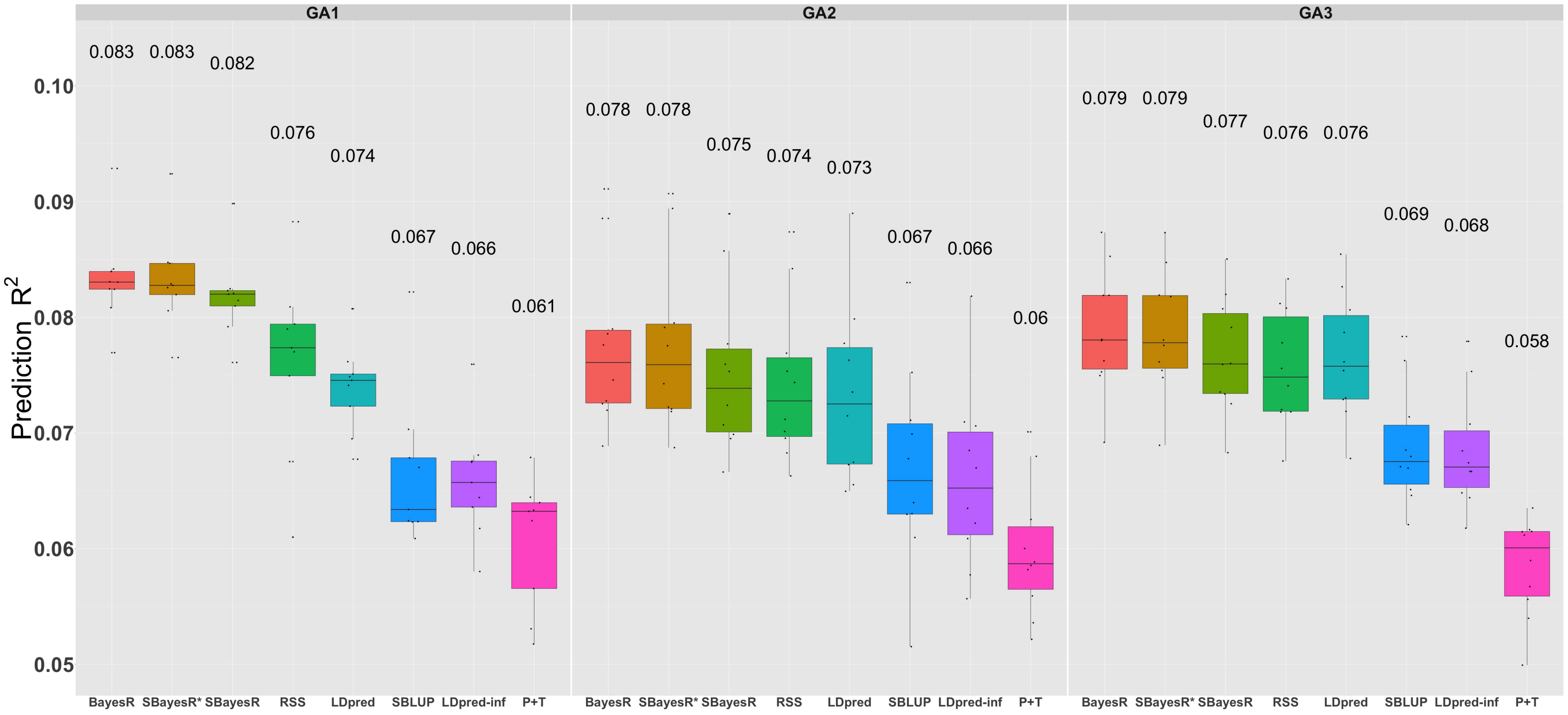

Supplementary Figure 3 Prediction accuracy performance using different methods in the simulation on chromosomes 21 and 22. Each panel displays boxplot summaries of the prediction R^2 (y-axis) in the 10,000 individual validation data set and an LD reference generated from a random subset of 50,000 individuals from the UKB. Each boxplot shows the prediction R^2 across the 10 replicates for each of the three simulation scenarios in the chromosome 21 and 22 simulation i.e., the first genetic architecture (GA1) contained two causal variants of large effect explaining 3% and 2% of the phenotypic variance respectively and a polygenic tail of 1,498 causal variants sampled from a N(0, 0.05/1, 498) distribution such that the expected total genetic variance explained by all variants was 0.1. The second architecture (GA2) was simulated under a BayesR model with three sets of causal variants: the first contained 1,445 causal variants sampled from a $N(0, 0.06/1445)$ distribution, the second contained 50 causal variants sampled from a $N(0, 0.02/50)$ distribution and the third five causal variants sampled from $N(0, 0.02/5)$ distribution. The third architecture (GA3) contained 1,500 variants sampled from a $N(0, 0.1/1500)$ distribution. SBayesR* corresponds to the analysis using the SBayesR model and the full set of 100,000 individuals used in the GWAS analysis to create the LD matrix. This LD matrix includes all pairwise correlations i.e., includes inter-chromosomal LD. The mean R^2 across the 10 replicates is displayed above the boxplot for each cohort. The box plot centre line is the median, the bottom and top of the box are the first (Q1) and third quartiles (Q3) and the lower and upper whiskers are Q1 – 1.5 IQR and Q3 + 1.5 IQR, respectively, where IQR = Q3 – Q1.

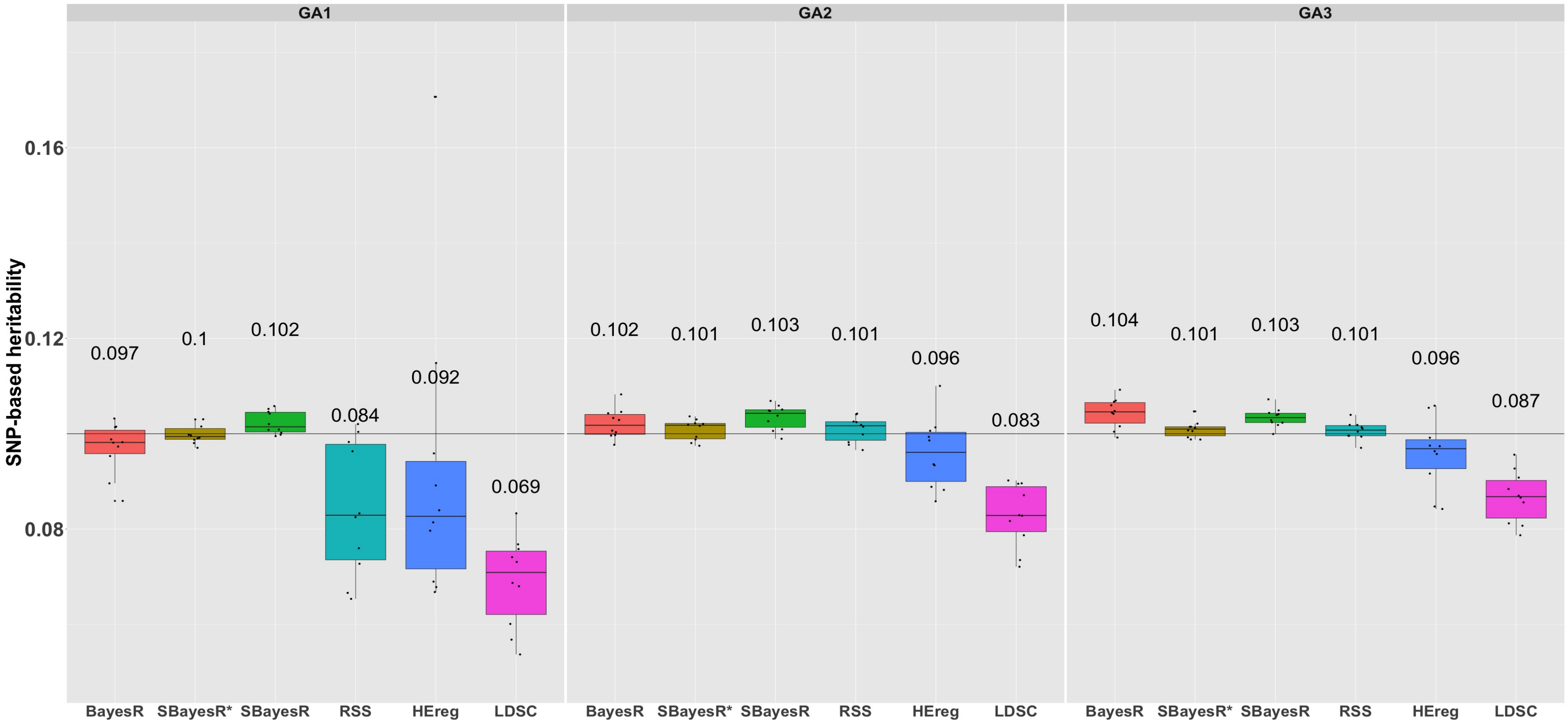

Supplementary Figure 4 SNP-based heritability (h^2_{SNP}) estimation for different methods in the simulation on chromosomes 21 and 22. Each panel displays boxplot summaries of the h^2_{SNP} estimates (y-axis) for each method (x-axis) across the 10 replicates in each of the three scenarios in the chromosome 21 and 22 simulation. Each trait has a simulated true $h^2_{SNP} = 0.1$ (horizontal line) and 1,500 causal variants. SBayesR* corresponds to the analysis using the SBayesR model and the full set of 100,000 individuals used in the GWAS analysis to create the LD matrix. The mean h^2_{SNP} across the 10 replicates is displayed above the boxplot for each method. See Supplementary Figure 1 for descriptions of the genetic architectures (GA1, GA2, GA3). The deflation of the LDSC estimate across scenarios should be interpreted with caution as it is a likely result of the use of the small number of variants in this simulation. Simulations (not shown) using chromosomes 1-3 and the GA3 simulation scenario show unbiased LDSC estimates.The box plot centre line is the median, the bottom and top of the box are the first (Q1) and third quartiles (Q3) and the lower and upper whiskers are Q1 – 1.5 IQR and Q3 + 1.5 IQR, respectively, where IQR = Q3 – Q1.

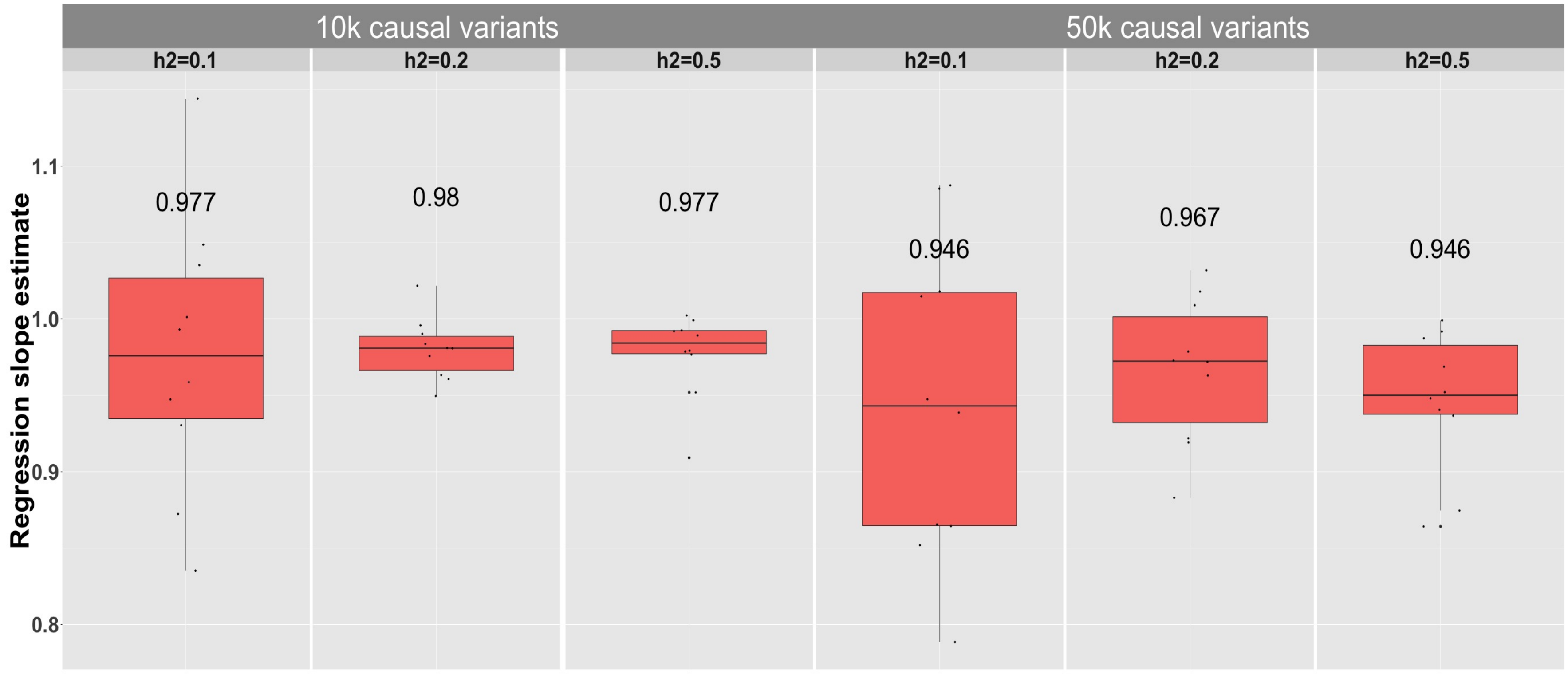

Supplementary Figure 5 Slope estimates from regression of observed phenotypic values on the predicted values from SBayesR for quantitative phenotypes in the genome-wide simulation studies. Each panel shows a boxplot summary of the estimated regression slope across the 10 replicates for each scenario with the mean displayed above each method's boxplot.The box plot centre line is the median, the bottom and top of the box are the first (Q1) and third quartiles (Q3) and the lower and upper whiskers are Q1 – 1.5 IQR and Q3 + 1.5 IQR, respectively, where IQR = Q3 – Q1.

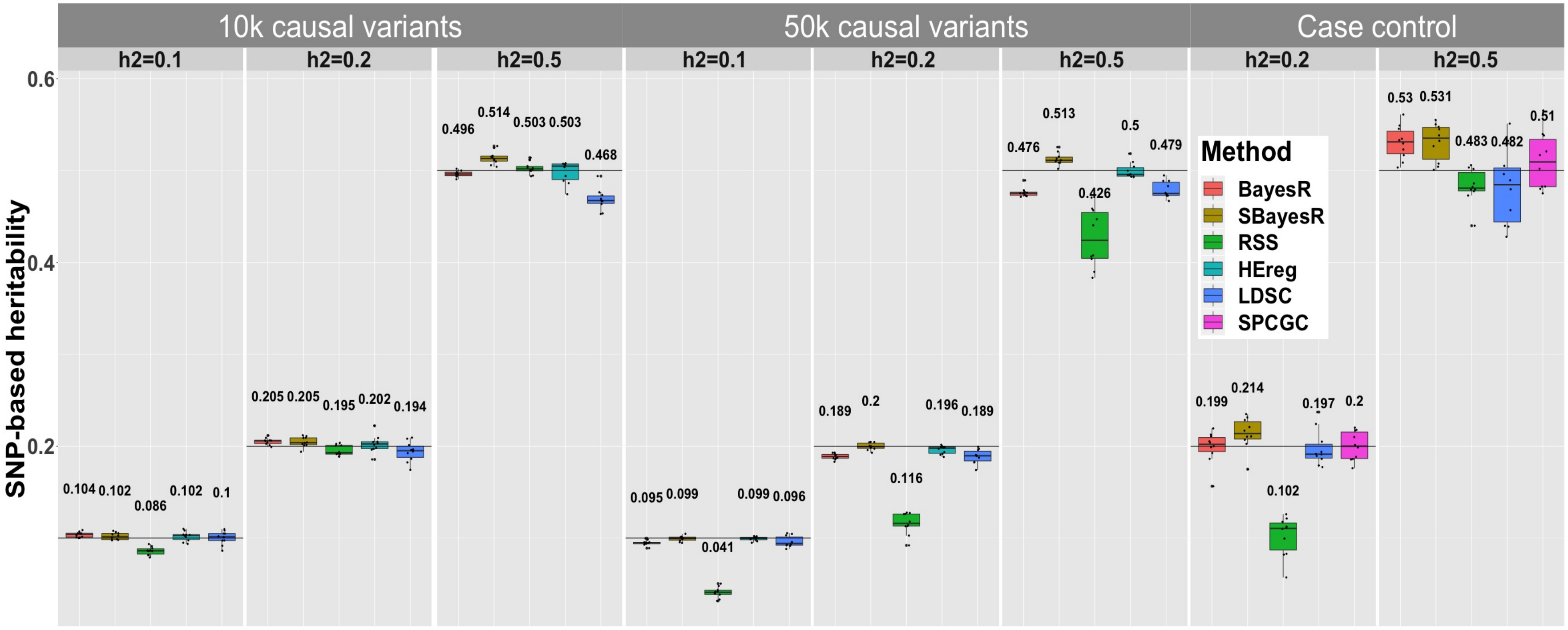

Supplementary Figure 6 SNP-based heritability (h^2_{SNP}) estimation performance for different methods in UKB genome-wide simulation. Each panel displays boxplot summaries of h^2_{SNP} estimates (y-axis) for each method (x-axis) across the 10 replicates for each of the six simulation scenarios that varied in the number of causal variants, 10k and 50k, and the true simulated $h^2_{SNP} = (0.1, 0.2, 0.5)$. Two genetic architecture scenarios were generated: 10,000 causal variants sampled under the SBayesR model i.e., 2500, 5000, and 2500 variants from each of $N(0, 0.01\sigma^2_\beta)$, $N(0, 0.1\sigma^2_\beta)$, and $N(0, \sigma^2_\beta)$ distributions respectively and $\sigma^2_\beta = 1$. For the second architecture, 50,000 causal variants were sampled from a standard normal distribution. For each replicate a new sample of causal variants was chosen at random from the set of 1,094,841 HapMap 3 variants. The mean h^2_{SNP} estimate across the 10 replicates is displayed above the boxplot for each method. Case-control phenotypes were generated from the liability threshold model using the 10,000 causal variants BayesR model and were generated using the GCTA software with a simulated disease prevalence of 0.05 and h^2_{SNP} = (0.2, 0.5). The box plot centre line is the median, the bottom and top of the box are the first (Q1) and third quartiles (Q3) and the lower and upper whiskers are Q1 – 1.5 IQR and Q3 + 1.5 IQR, respectively, where IQR = Q3 – Q1.

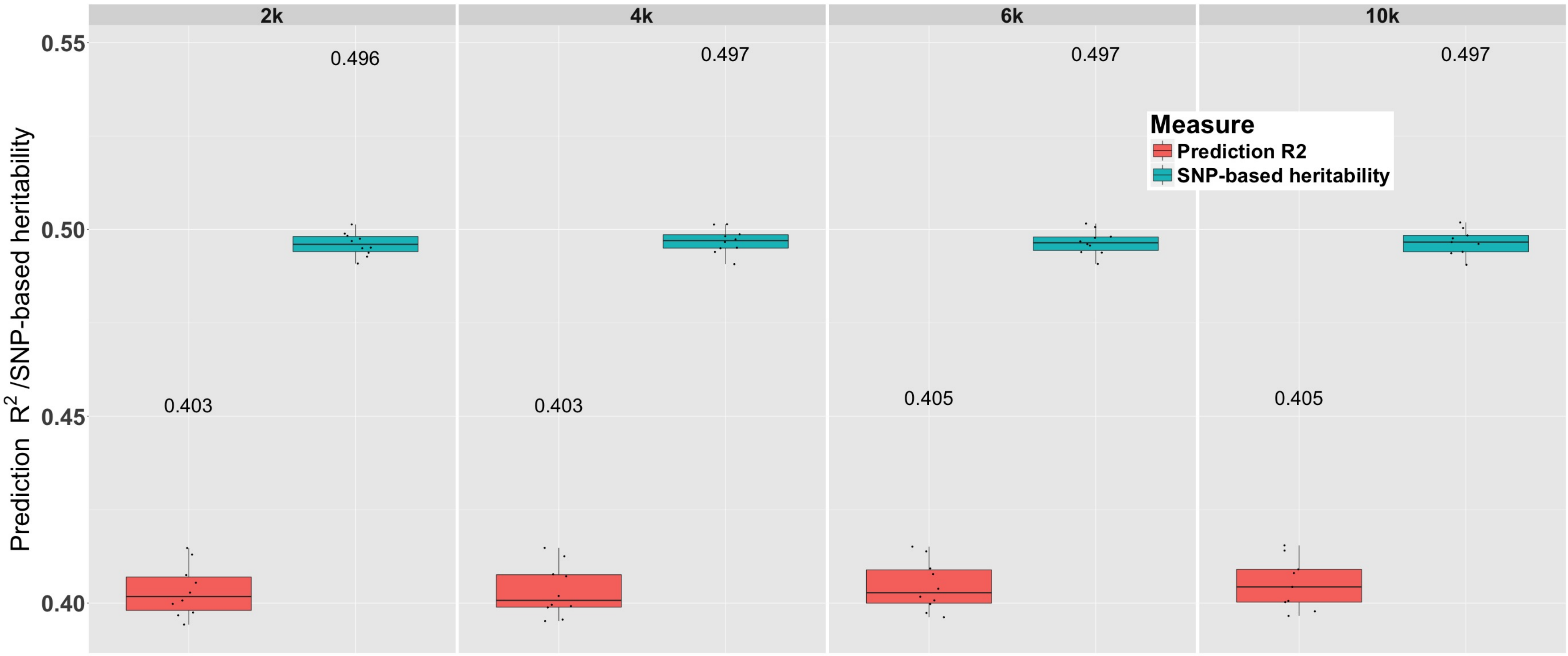

Supplementary Figure 7 BayesR prediction accuracy and SNP-based heritability (h^2_{SNP}) estimation change with MCMC chain length for one scenario of UKB genome-wide simulation. Each panel displays boxplot summaries of prediction R^2 and h^2_{SNP} estimates (y-axis) for 2,000 (2k), 4,000 (4k), 6,000 (6k) and 10,000 (10k) MCMC iterations of the BayesR method[6]. Each boxplot shows the results from the 10 replicates in the 10k (simulated under a BayesR model) causal variant and the true simulated $h^2_{SNP} = 0.5$ scenario. The mean prediction R^2 and h^2_{SNP} estimates across the 10 replicates are displayed above the relevant boxplot. The mean run time for each of the 2k, 4k, 6k and 10k MCMC iterations scenarios was 32.5, 56.7, 77.9 and 109.8 hours respectively. The box plot centre line is the median, the bottom and top of the box are the first (Q1) and third quartiles (Q3) and the lower and upper whiskers are Q1 – 1.5 IQR and Q3 + 1.5 IQR, respectively, where IQR = Q3 – Q1.

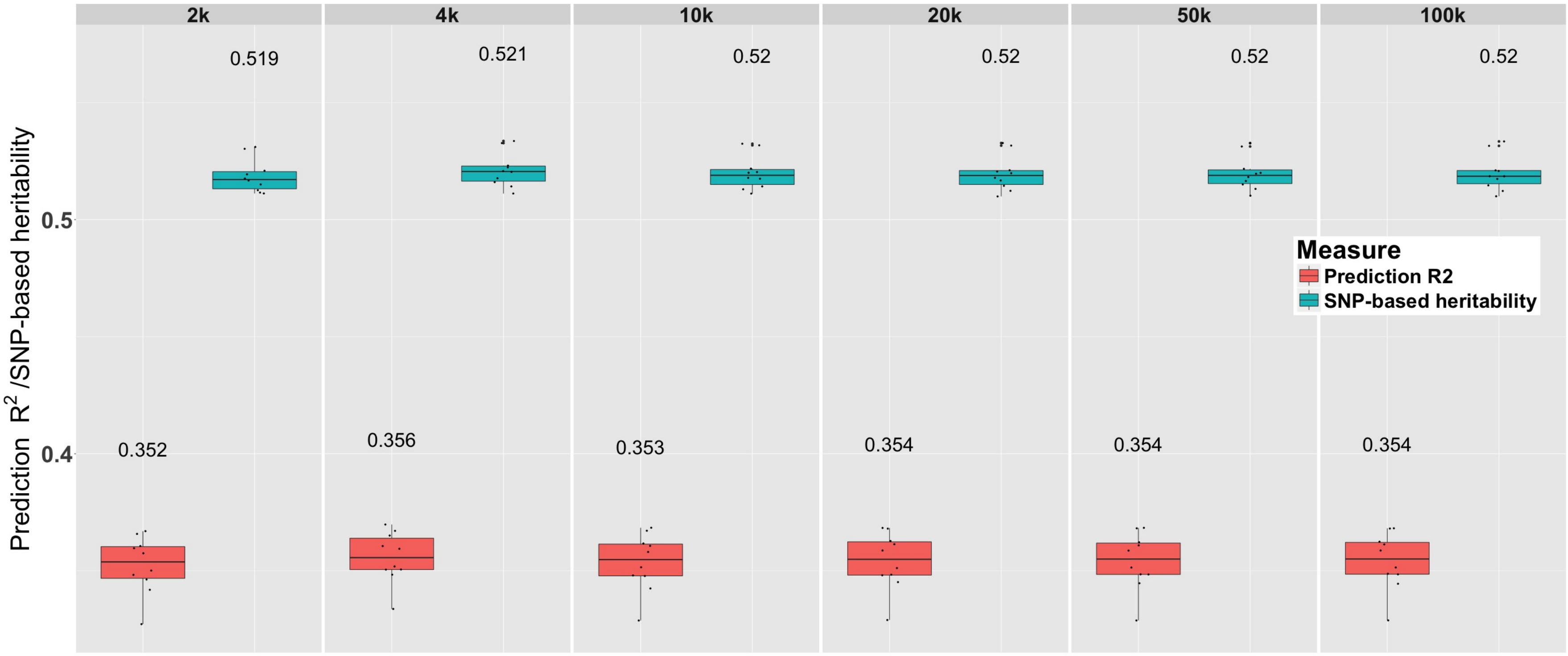

Supplementary Figure 8 SBayesR prediction accuracy and SNP-based heritability (h^2_{SNP}) estimation change with MCMC chain length for one scenario of UKB genome-wide simulation. Each panel displays boxplot summaries of prediction R^2 and h^2_{SNP} estimates (y-axis) for 2,000 (2k), 4,000 (4k), 10,000 (10k), 20,000 (20k), 50,000 (50k), and 100,000 (100k) MCMC iterations of the SBayesR method. Each boxplot shows the results from the 10 replicates in the 10k (simulated under a BayesR model) causal variant and the true simulated $h^2_{SNP} = 0.5$ scenario. The mean prediction R^2 and h^2_{SNP} estimates across the 10 replicates are displayed above the relevant boxplot. The mean run time for each of the 2k, 4k, 10k, 20k, 50k and 100k MCMC iterations scenarios was 0.35, 0.78, 4.4, 4.5, 7.7, and 14.6 hours respectively. The box plot centre line is the median, the bottom and top of the box are the first (Q1) and third quartiles (Q3) and the lower and upper whiskers are Q1 – 1.5 IQR and Q3 + 1.5 IQR, respectively, where IQR = Q3 – Q1.

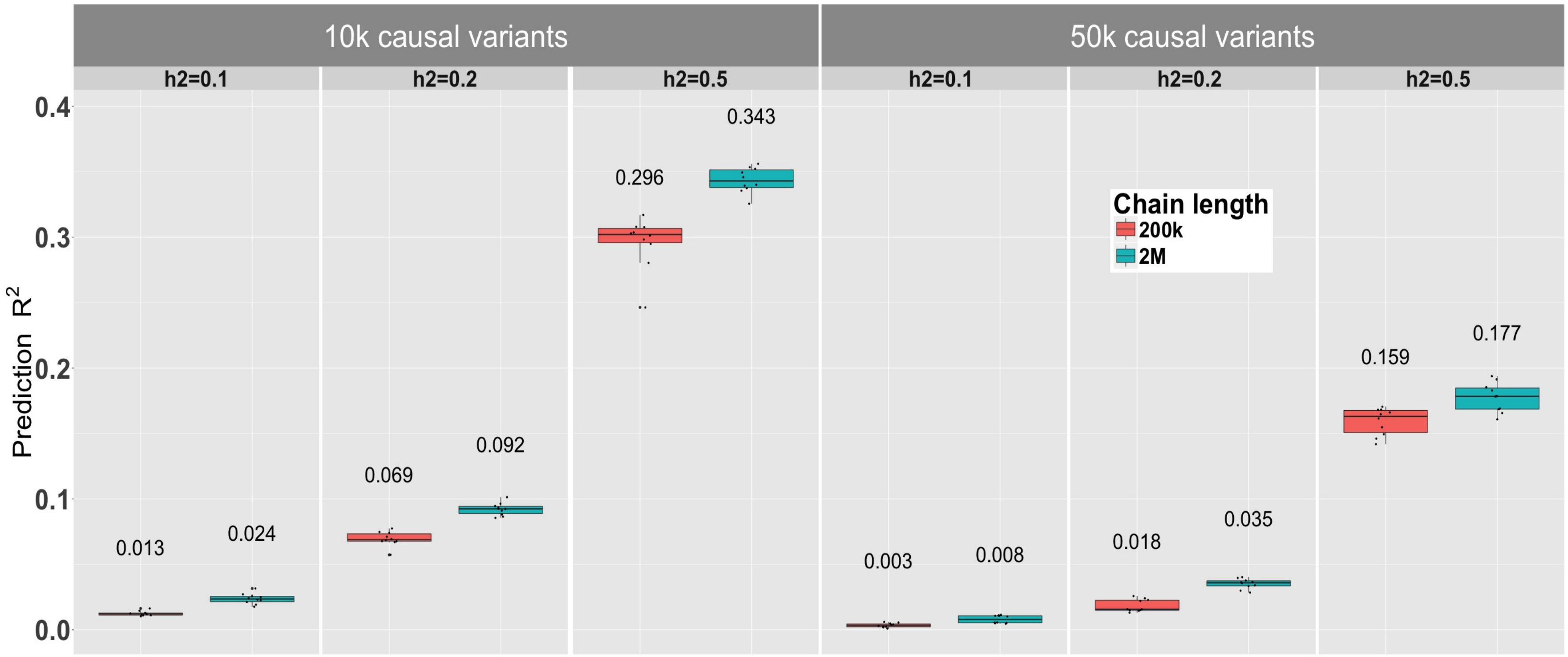

Supplementary Figure 9 Regression with Summary Statistics (RSS)[1] prediction accuracy for results generated from 200,000 (200k) and 2,000,000 (2M) iterations of the MCMC chain for all scenarios of the UKB genome-wide simulation. Each panel displays boxplot summaries of the prediction R^2 (y-axis) for RSS across the 10 replicates for each of the six simulation scenarios that varied in the number of causal variants, 10k and 50k, and the true simulated $h^2_{SNP} = (0.1, 0.2, 0.5)$. Two genetic architecture scenarios were generated: 10,000 causal variants sampled under the SBayesR model i.e., 2500, 5000, and 2500 variants from each of $N(0, 0.01\sigma^2_\beta)$, $N(0, 0.1\sigma^2_\beta)$, and $N(0, \sigma^2_\beta)$ distributions respectively and $\sigma^2_\beta = 1$. For the second architecture, 50,000 causal variants were sampled from a standard normal distribution. The mean prediction R^2 value across the 10 replicates is displayed above the relevant boxplot. The box plot centre line is the median, the bottom and top of the box are the first (Q1) and third quartiles (Q3) and the lower and upper whiskers are Q1 – 1.5 IQR and Q3 + 1.5 IQR, respectively, where IQR = Q3 – Q1.

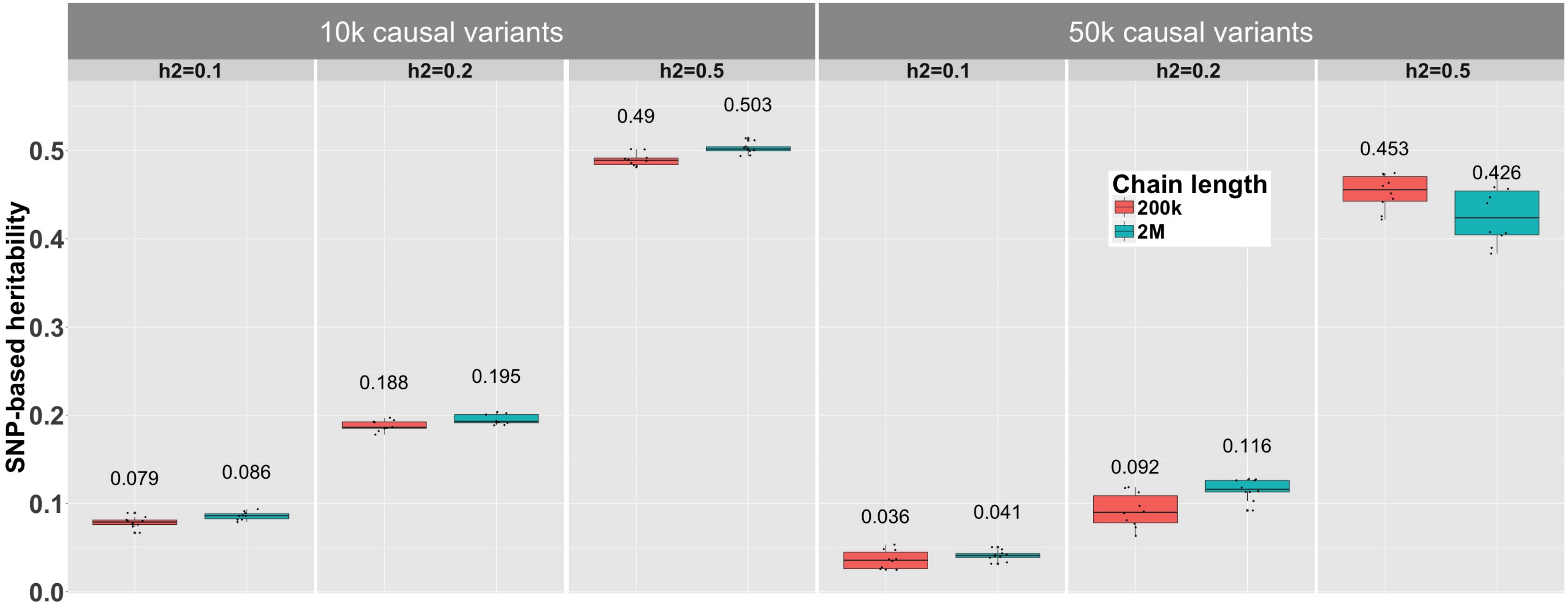

Supplementary Figure 10 Regression with Summary Statistics (RSS)[1] SNP-based heritability (h^2_{SNP}) estimates for results generated from 200,000 (200k) and 2,000,000 (2M) iterations of the MCMC chain for all scenarios of the UKB genome-wide simulation. Each panel displays boxplot summaries of h^2_{SNP} estimates (y-axis) for RSS across the 10 replicates for each of the six simulation scenarios that varied in the number of causal variants, 10k and 50k, and the true simulated $h^2_{SNP} = (0.1, 0.2, 0.5)$. Two genetic architecture scenarios were generated: 10,000 causal variants sampled under the SBayesR model i.e., 2500, 5000, and 2500 variants from each of $N(0, 0.01\sigma^2_\beta)$, $N(0, 0.1\sigma^2_\beta)$, and $N(0, \sigma^2_\beta)$ distributions respectively and $\sigma^2_\beta = 1$. For the second architecture, 50,000 causal variants were sampled from a standard normal distribution. The mean prediction h^2_{SNP} estimate across the 10 replicates is displayed above the relevant boxplot.The box plot centre line is the median, the bottom and top of the box are the first (Q1) and third quartiles (Q3) and the lower and upper whiskers are Q1 – 1.5 IQR and Q3 + 1.5 IQR, respectively, where IQR = Q3 – Q1.

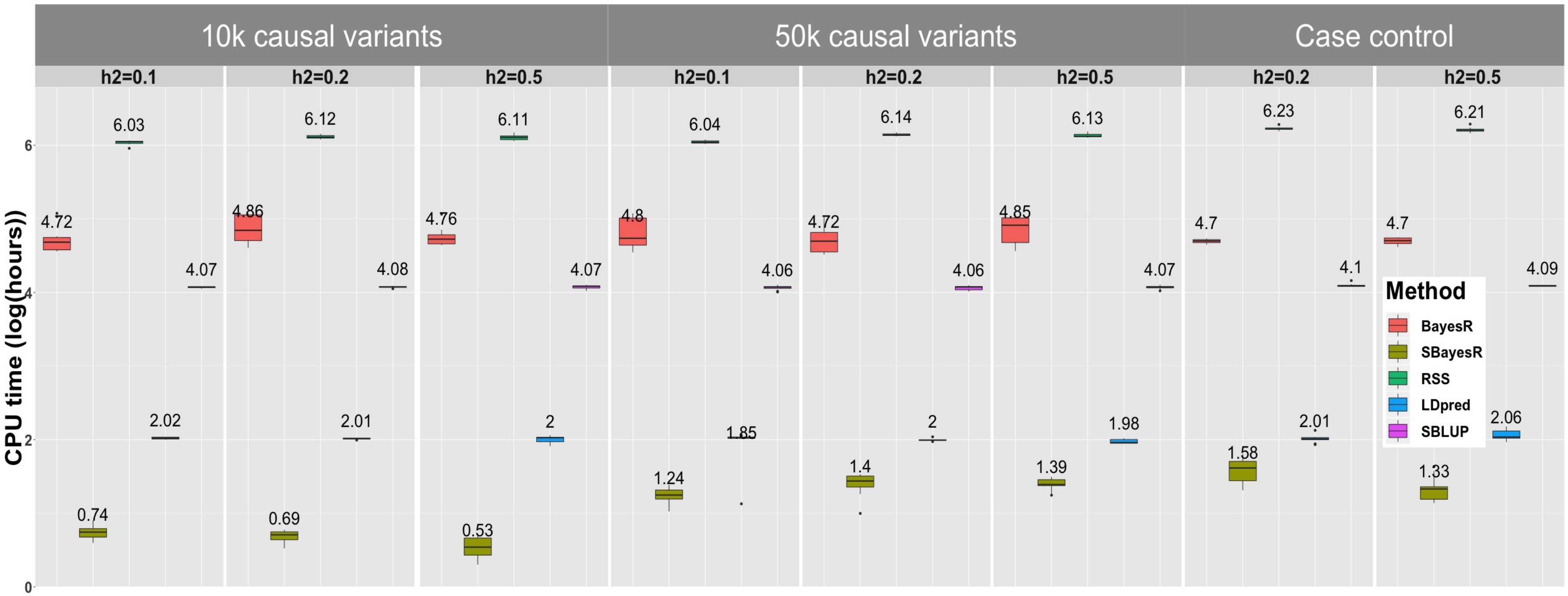

Supplementary Figure 11 Runtime (log(hours)) comparison for BayesR, SBayesR, RSS, LDpred and SBLUP for UKB genome-wide simulation. Each panel shows a boxplot summary of runtime across the 10 replicates for each scenario with the mean runtime displayed above each method's boxplot. The runtime for RSS, LDpred and SBLUP represents the sum over the runtimes for each chromosome. Results for P+T, HEreg, S-PCGC and LDSC are not shown as they required relatively minimal computing resources. The box plot centre line is the median, the bottom and top of the box are the first (Q1) and third quartiles (Q3) and the lower and upper whiskers are Q1 – 1.5 IQR and Q3 + 1.5 IQR, respectively, where IQR = Q3 – Q1.

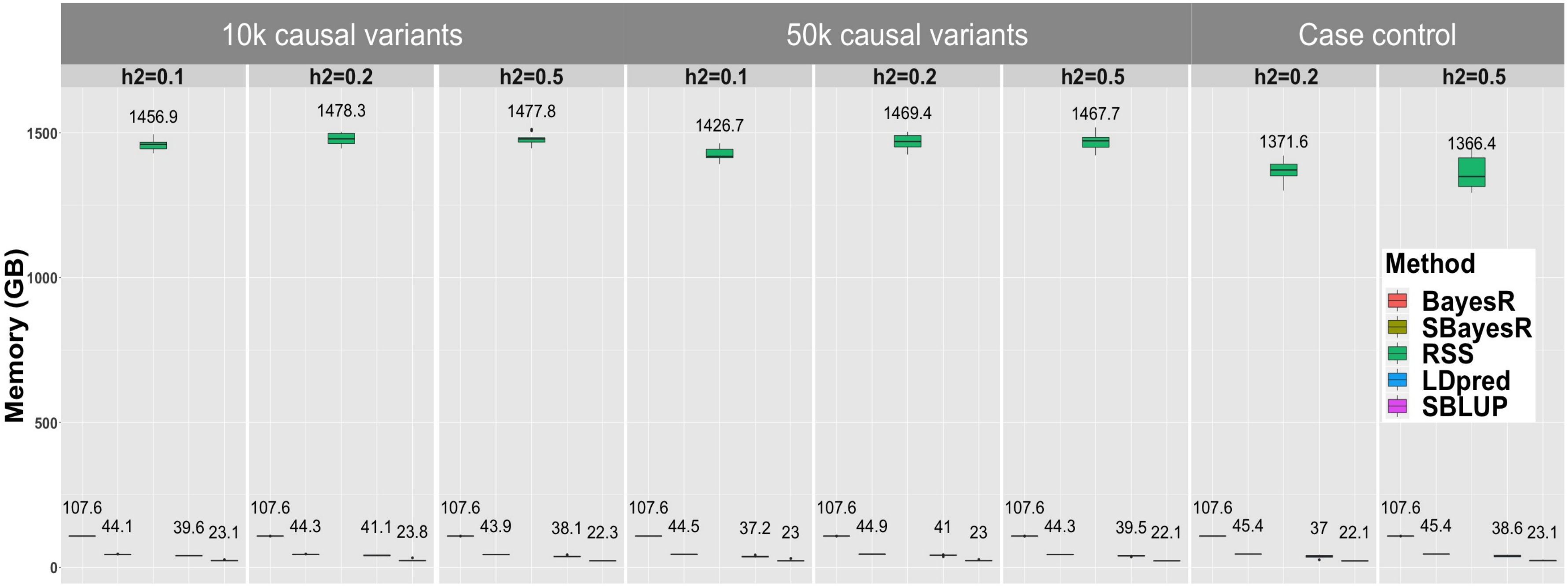

Supplementary Figure 12 Memory usage in gigabytes (GB) comparison for BayesR, SBayesR, RSS, LDpred and SBLUP for UKB genome-wide simulation. Each panel shows a boxplot summary of memory usage across the 10 replicates for each scenario with the mean memory displayed above each method's boxplot. The memory for RSS, and SBLUP represents the sum over the memory usage for each chromosome. The maximum chromosome memory requirement for RSS is for chromosome two which required on average 192 GB (SE = 5 GB) of RAM across simulation scenarios. Results for P+T, HEreg, S-PCGC and LDSC are not shown as they required relatively minimal computing resources. The box plot centre line is the median, the bottom and top of the box are the first (Q1) and third quartiles (Q3) and the lower and upper whiskers are Q1 – 1.5 IQR and Q3 + 1.5 IQR, respectively, where IQR = Q3 – Q1.

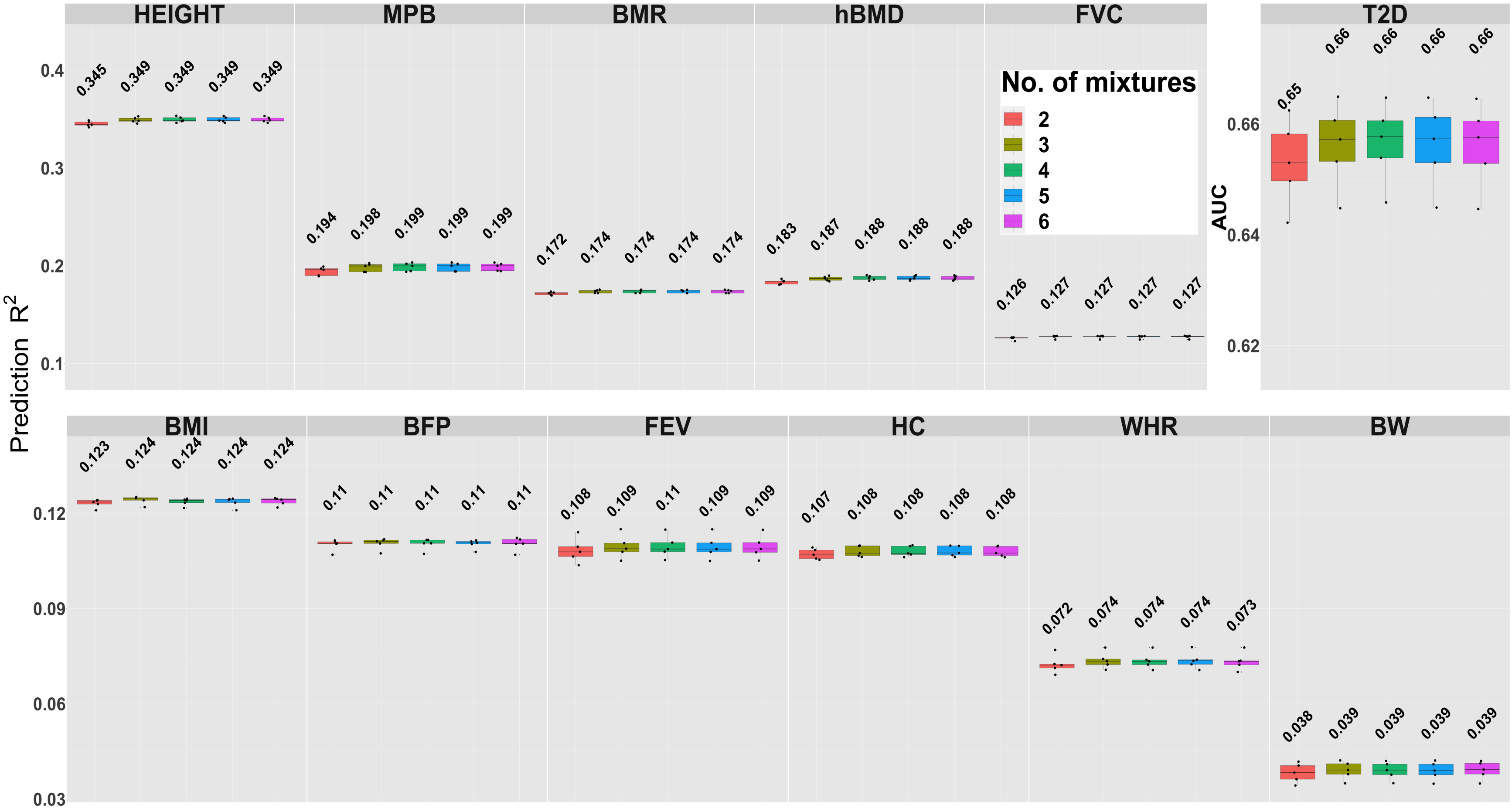

Supplementary Figure 13 SBayesR prediction accuracy in five-fold cross-validation for 12 traits in the UK Biobank using different numbers of mixture components. Panel headings describe the abbreviation for 12 traits including: standing height (HEIGHT, n=347,106), male pattern baldness (MPB, n =125,157), basal metabolic rate (BMR, n=341,819), heel bone mineral density T-score (hBMD, n=197,789), forced vital capacity (FVC, n=317,502), type-2 diabetes (T2D, n=274,271) body mass index (BMI, n=346,738), body fat percentage (BFP, n=341,633), forced expiratory volume in one-second (FEV, n=317,502), hip circumference (HC, n=347,231), waist-to-hip ratio (WHR, n=347,198) and birth weight (BW, n=197,778). Each panel shows a boxplot summary of the prediction R^2 across the five folds with the mean across the five folds displayed above each distribution number boxplot. Traits are ordered by mean estimated h^2_{SNP} (see Supplementary Figure 16) from highest to lowest. The box plot centre line is the median, the bottom and top of the box are the first (Q1) and third quartiles (Q3) and the lower and upper whiskers are Q1 – 1.5 IQR and Q3 + 1.5 IQR, respectively, where IQR = Q3 – Q1.

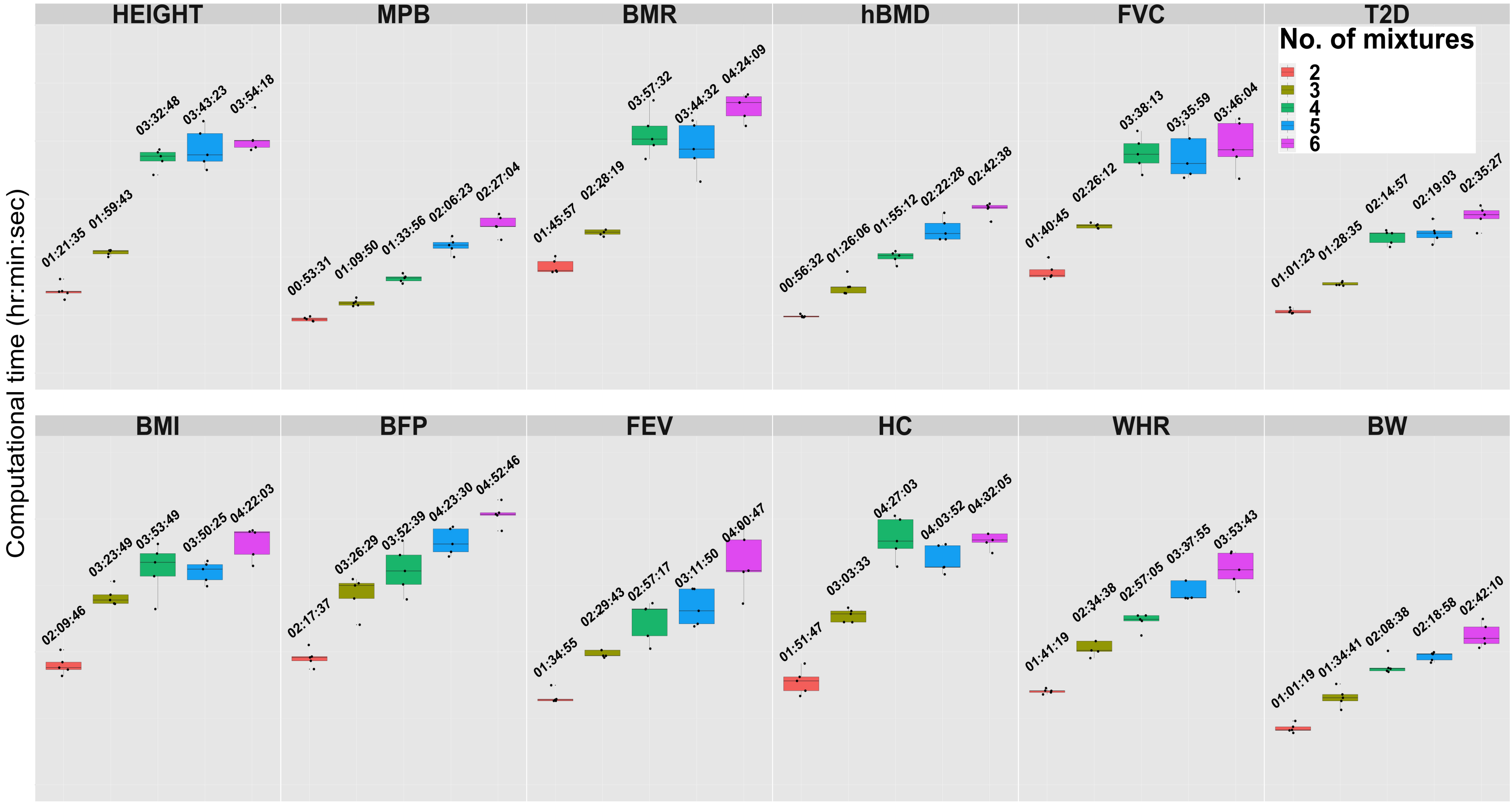

Supplementary Figure 14 SBayesR computational time change in five-fold cross-validation for 12 traits in the UK Biobank using different numbers of mixture components. Panel headings describe the abbreviation for 12 traits including: standing height (HEIGHT, n=347,106), male-pattern baldness (MPB, n =125,157), basal metabolic rate (BMR, n=341,819), heel bone mineral density T-score (hBMD, n=197,789), forced vital capacity (FVC, n=317,502), type-2 diabetes (T2D, n=274,271) body mass index (BMI, n=346,738), body fat percentage (BFP, n=341,633), forced expiratory volume in one-second (FEV, n=317,502), hip circumference (HC, n=347,231), waist-to-hip ratio (WHR, n=347,198) and birth weight (BW, n=197,778). Each panel shows a boxplot summary of the computation time (hours:minutes:seconds) across the five folds with the mean across the five folds displayed above each distribution number boxplot. Traits are ordered by mean estimated h^2_{SNP} (see Supplementary Figure 16) from highest to lowest. The box plot centre line is the median, the bottom and top of the box are the first (Q1) and third quartiles (Q3) and the lower and upper whiskers are Q1 – 1.5 IQR and Q3 + 1.5 IQR, respectively, where IQR = Q3 – Q1.

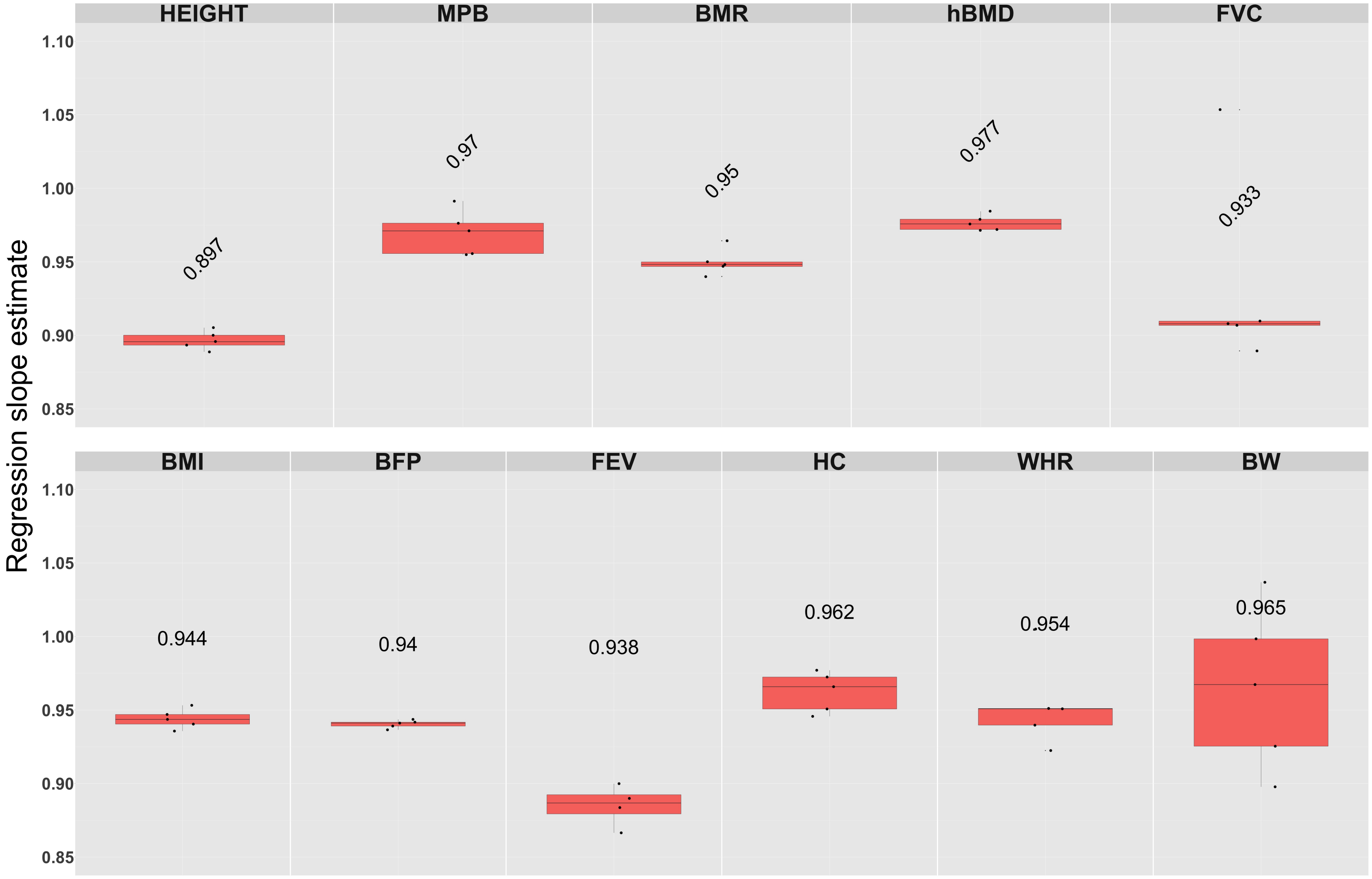

Supplementary Figure 15 Slope estimates from regression of observed phenotypic values on the predicted values from SBayesR for quantitative phenotypes in the UK Biobank cross-validation studies. Each panel shows a boxplot summary of the estimated regression slope across the five folds for each trait with the mean displayed above each method's boxplot. The box plot centre line is the median, the bottom and top of the box are the first (Q1) and third quartiles (Q3) and the lower and upper whiskers are Q1 – 1.5 IQR and Q3 + 1.5 IQR, respectively, where IQR = Q3 – Q1.

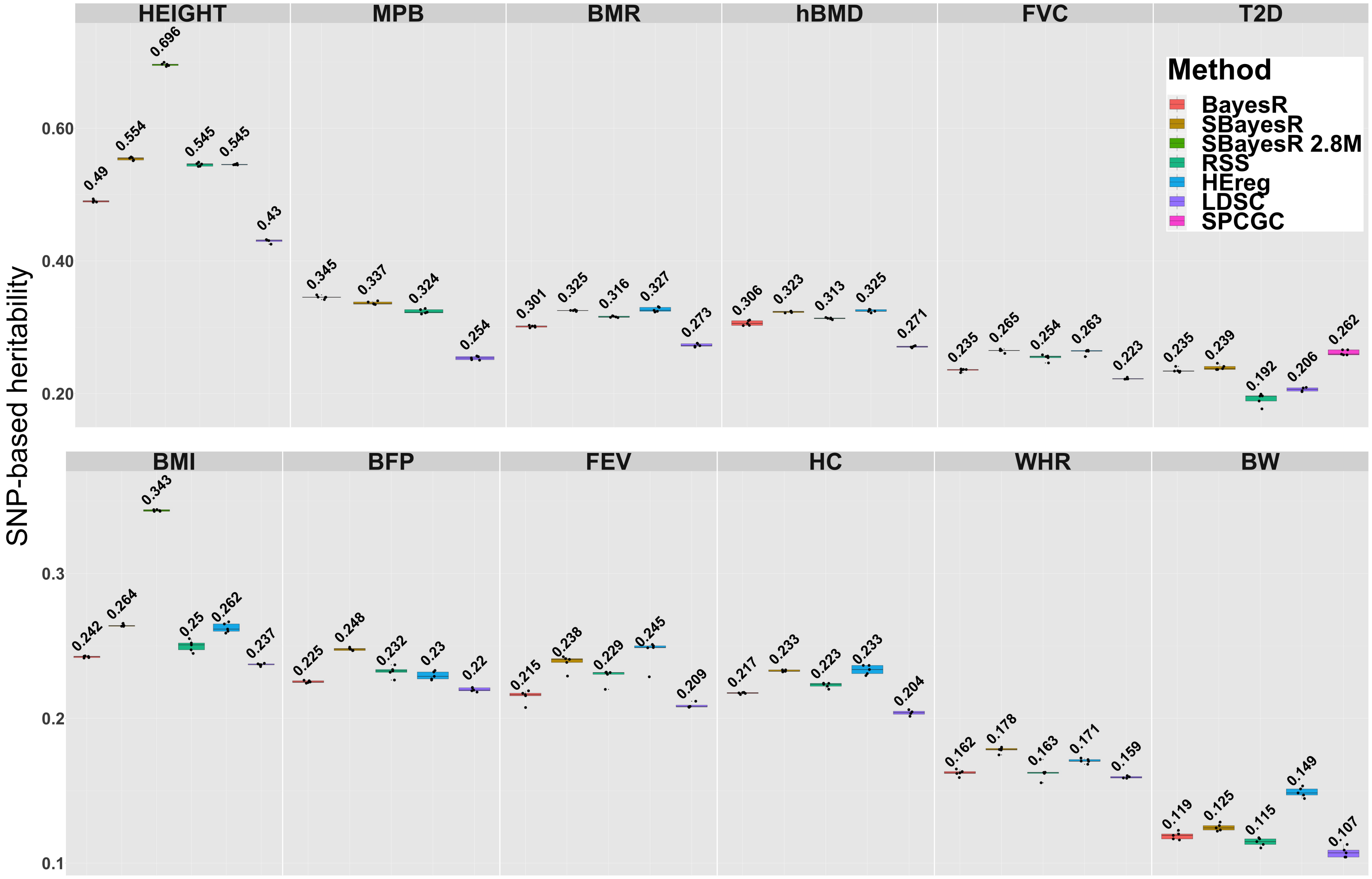

Supplementary Figure 16 SNP-based heritability (h^2_{SNP}) estimation performance for different methods in the 5-fold cross-validation analysis of 12 quantitative traits in the UKB. Panel headings describe the abbreviation for the 12 quantitative traits (see Supplementary Figure 13 for a description of the traits names). Each panel shows a boxplot summary of the h^2_{SNP} estimates across the five folds with the mean across the five folds displayed above each method's boxplot. The box plot centre line is the median, the bottom and top of the box are the first (Q1) and third quartiles (Q3) and the lower and upper whiskers are Q1 – 1.5 IQR and Q3 + 1.5 IQR, respectively, where IQR = Q3 – Q1.

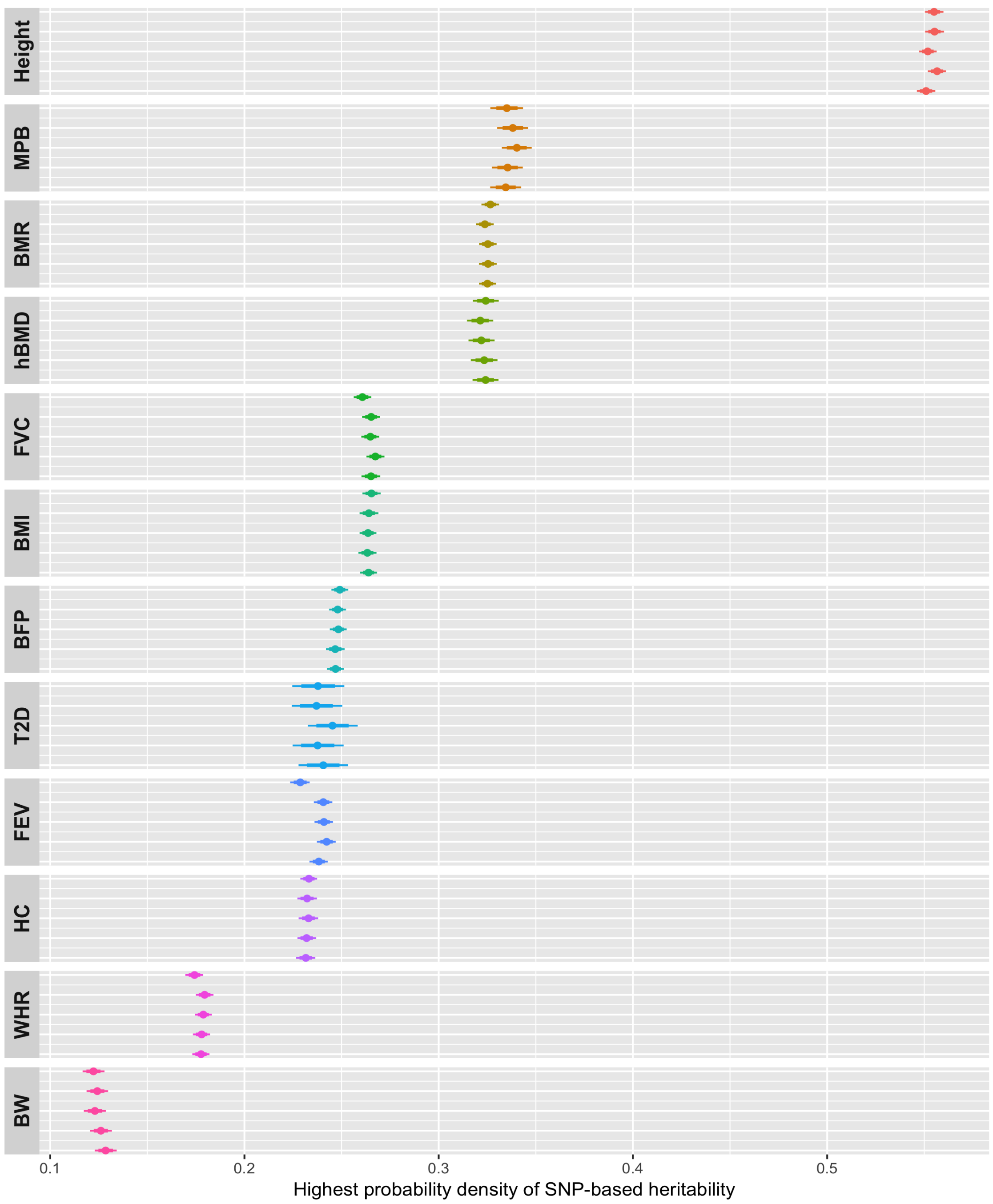

Supplementary Figure 17 SNP-based heritability (h^2_{SNP}) posterior mean estimates and highest-probability densities (HPD) for SBayesR results from five fold cross-validation analysis of 12 traits in the UKB. Y-axis labels describe the abbreviation for the 12 traits (see Supplementary Figure 13 for a description of the traits names). The standard error of h^2_{SNP} across folds for the traits as they appear in the plot are 0.0024, 0.0024, 0.0010, 0.0012, 0.0024, 0.0008, 0.0010, 0.0010, 0.0054, 0.0006, 0.0020, 0.0025. The mean of the reported posterior standard error of h^2_{SNP} across folds for the traits as they appear in the plot are 0.0024, 0.0040, 0.0023, 0.0034, 0.0024, 0.0023, 0.0023, 0.0016, 0.0023, 0.0024, 0.0022, 0.0029. The point represents the mean, thick line the 95% HPD and the thin line the 80% HPD.

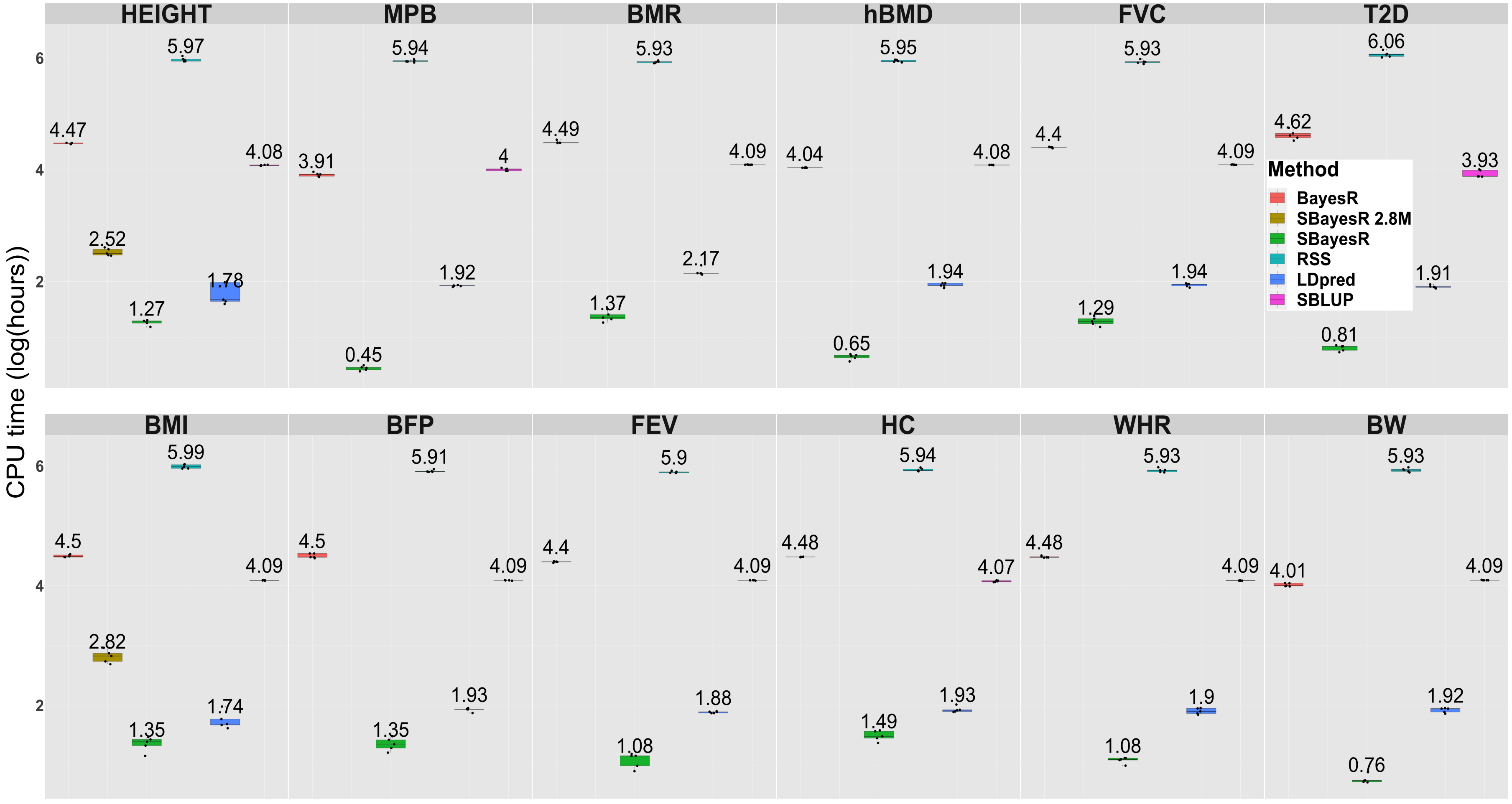

Supplementary Figure 18 Runtime (log(hours)) comparison for BayesR, SBayesR, RSS, LDpred and SBLUP in cross-validation analysis of 12 traits in the UKB. Panel headings describe the abbreviation for 12 traits including: standing height (HEIGHT, n=347,106), male-pattern baldness (MPB, n =125,157), basal metabolic rate (BMR, n=341,819), heel bone mineral density T-score (hBMD, n=197,789), forced vital capacity (FVC, n=317,502), type-2 diabetes (T2D, n=274,271) body mass index (BMI, n=346,738), body fat percentage (BFP, n=341,633), forced expiratory volume in one-second (FEV, n=317,502), hip circumference (HC, n=347,231), waist-to-hip ratio (WHR, n=347,198) and birth weight (BW, n=197,778). Each panel shows a boxplot summary of runtime with the mean across the five folds displayed above each method's boxplot. Results for RSS, LDpred and SBLUP represent the sum over time for each chromosome-wise analysis. Results for RSS and SBayesR do not include the time to compute the LD reference matrix. Results for P+T, HEreg, S-PCGC and LDSC are not shown as they required relatively minimal computing resources. The box plot centre line is the median, the bottom and top of the box are the first (Q1) and third quartiles (Q3) and the lower and upper whiskers are Q1 – 1.5 IQR and Q3 + 1.5 IQR, respectively, where IQR = Q3 – Q1.

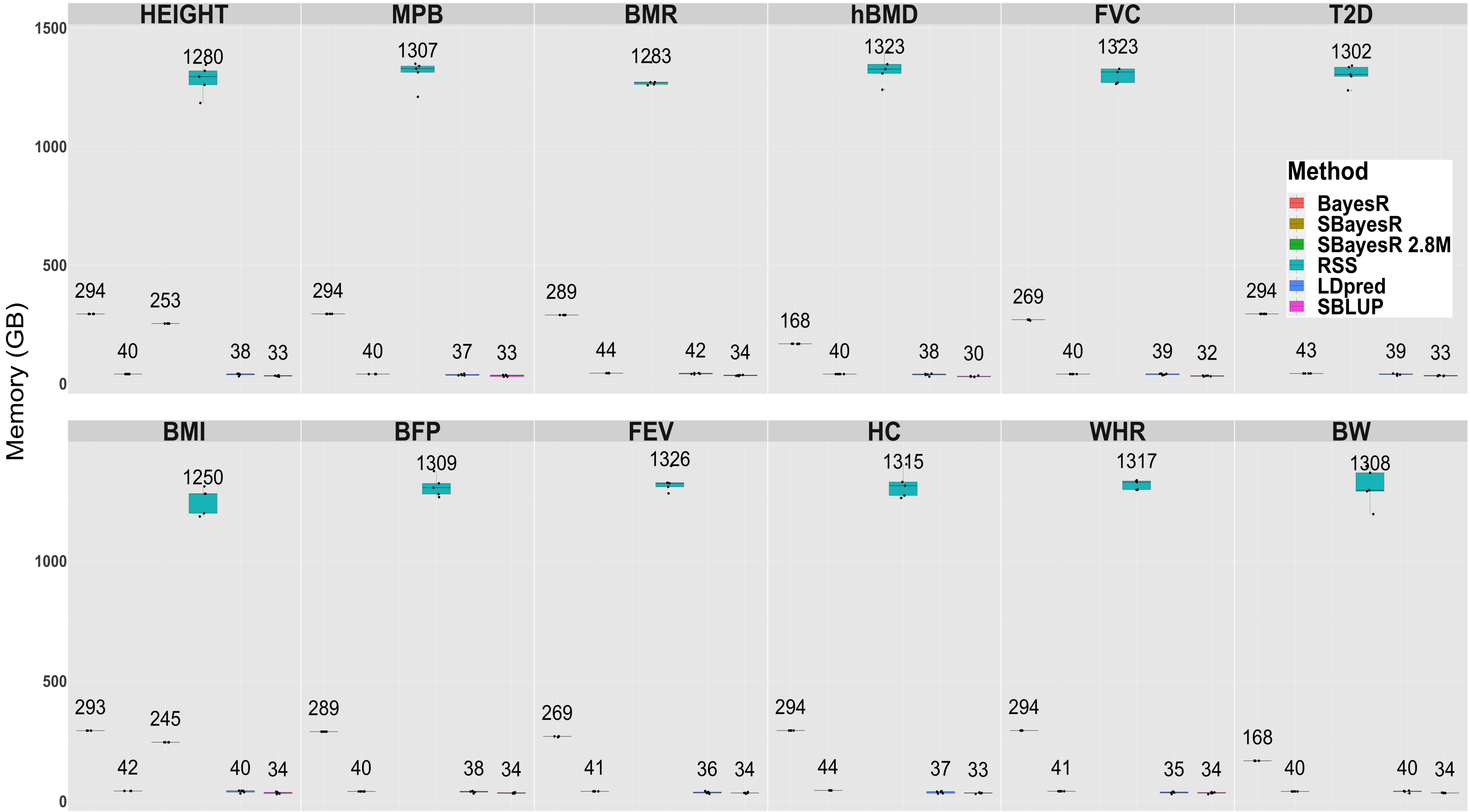

Supplementary Figure 19 Memory usage comparison in gigabytes (GB) for cross validation analysis of 10 quantitative traits in the UKB. Panels headings describe the abbreviation for the 10 quantitative traits. Each panel shows a boxplot summary of memory usage across the five folds with the mean across the five folds displayed above each method's boxplot. Results for RSS and SBLUP represent the sum over memory for each chromosome-wise analysis. Results for RSS and SBayesR do not include the memory required to compute the LD reference matrix. See Supplementary Figure 16 for description of trait abbreviations. The maximum chromosome memory requirement for RSS is for chromosome one which required on average 174 GB (SE = 22 GB) of RAM across simulation scenarios. Results for HEreg, S-PCGC and LDSC are not shown as they required relatively minimal computing resources. The box plot centre line is the median, the bottom and top of the box are the first (Q1) and third quartiles (Q3) and the lower and upper whiskers are Q1 – 1.5 IQR and Q3 + 1.5 IQR, respectively, where IQR = Q3 – Q1.

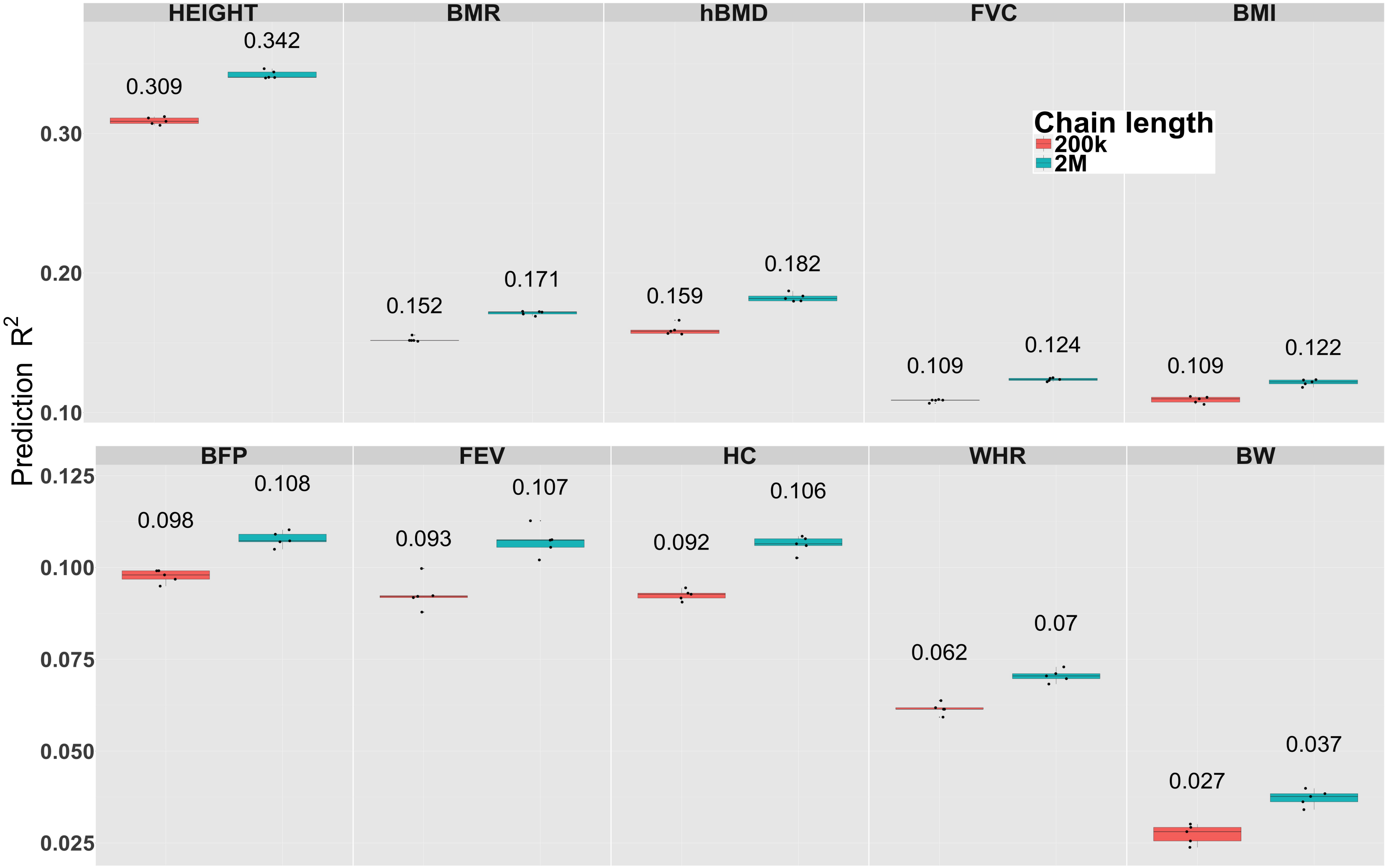

Supplementary Figure 20 Regression with Summary Statistics (RSS)[1] prediction accuracy for results generated from 200,000 (200k) and 2,000,000 (2M) iterations of the MCMC chain in the 5-fold cross-validation analysis of 10 quantitative traits in the UKB. Panel headings describe the abbreviation for the 10 quantitative traits including: standing height (HEIGHT), basal metabolic rate (BMR), heel bone mineral density T-score (hBMD), forced vital capacity (FVC), body mass index (BMI), body fat percentage (BFP), forced expiratory volume in one-second (FEV), hip circumference (HC), waist-to-hip ratio (WHR) and birth weight (BW). Each panel shows a boxplot summary of the prediction R^2 across the five folds with the mean across the five folds displayed above each boxplot. The box plot centre line is the median, the bottom and top of the box are the first (Q1) and third quartiles (Q3) and the lower and upper whiskers are Q1 – 1.5 IQR and Q3 + 1.5 IQR, respectively, where IQR = Q3 – Q1.

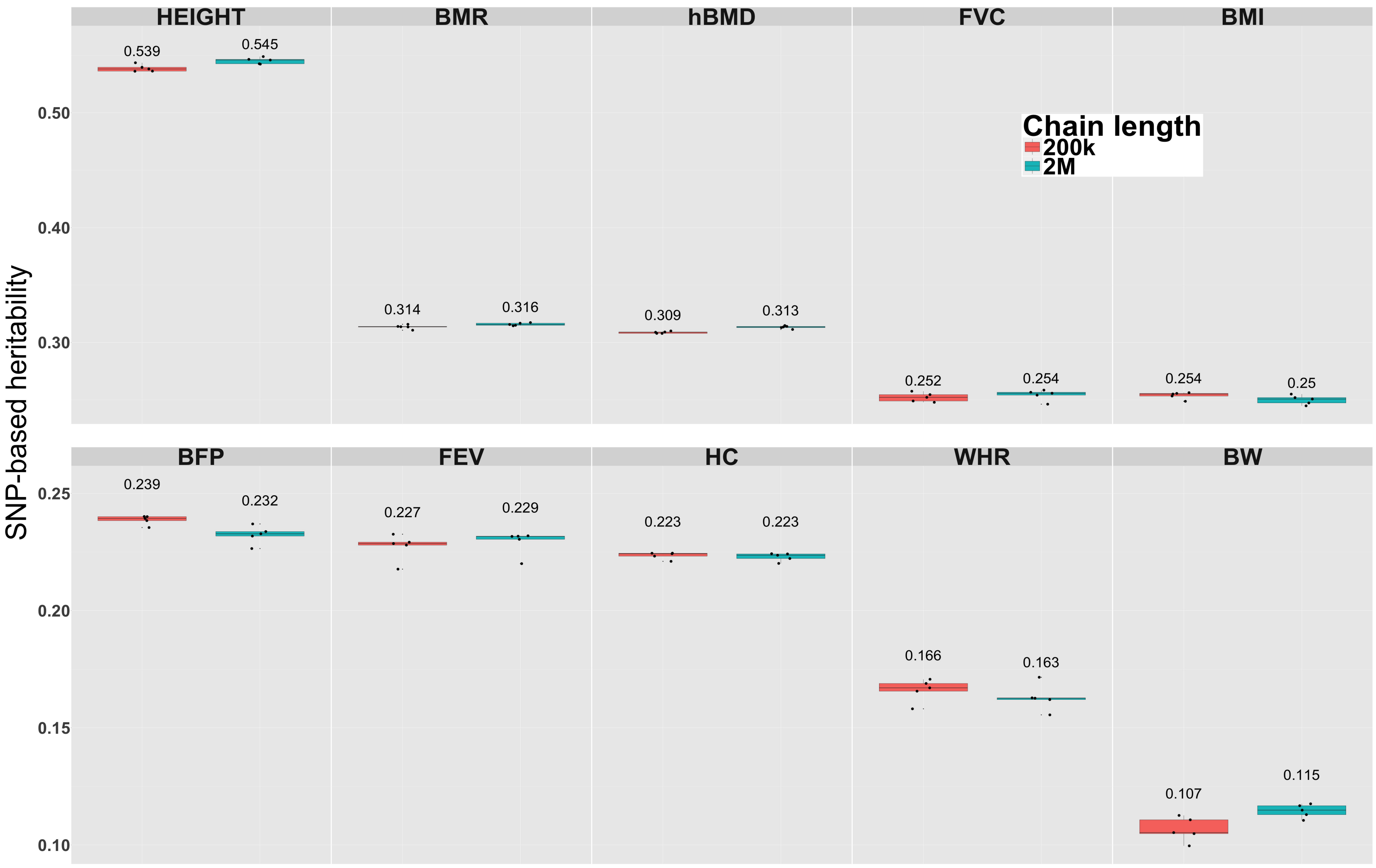

Supplementary Figure 21 Regression with Summary Statistics (RSS)[1] SNP-based heritability (h^2_{SNP}) estimates for results generated from 200,000 (200k) and 2,000,000 (2M) iterations of the MCMC chain in the 5-fold cross-validation analysis of 10 quantitative traits in the UKB. Panel headings describe the abbreviation for the 10 quantitative traits including: standing height (HEIGHT), basal metabolic rate (BMR), heel bone mineral density T-score (hBMD), forced vital capacity (FVC), body mass index (BMI), body fat percentage (BFP), forced expiratory volume in one-second (FEV), hip circumference (HC), waist-to-hip ratio (WHR) and birth weight (BW). Each panel shows a boxplot summary of the h^2_{SNP} estimates across the five folds with the mean across the five folds displayed above each boxplot. The box plot centre line is the median, the bottom and top of the box are the first (Q1) and third quartiles (Q3) and the lower and upper whiskers are Q1 – 1.5 IQR and Q3 + 1.5 IQR, respectively, where IQR = Q3 – Q1.

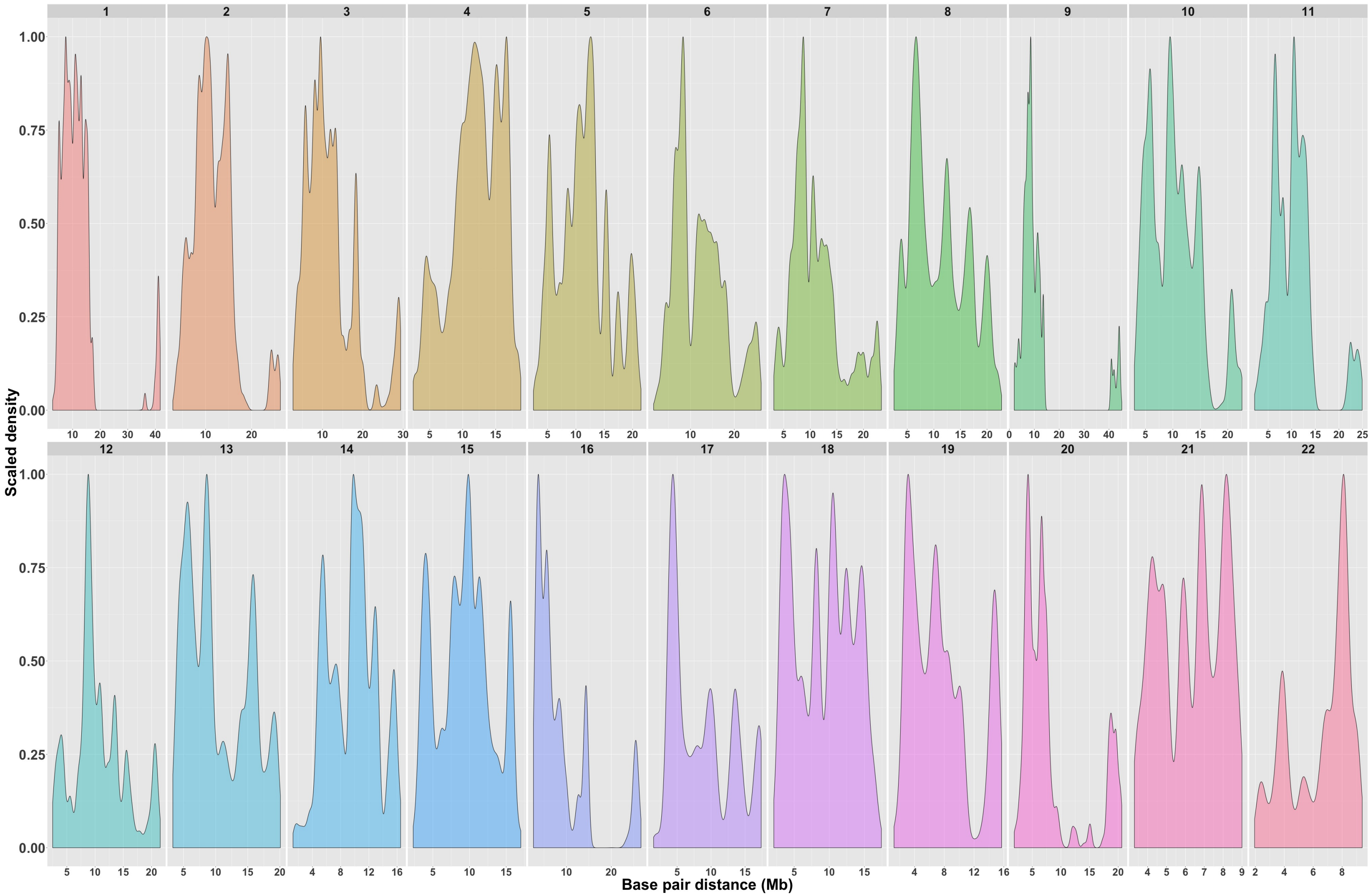

Supplementary Figure 22 Variability in per variant window width (measure in mega bases (Mb)) from the shrunk-sparse LD correlation matrix within chromosome for each of 1.09 million HapMap3 variants in the UKB. The chromosome-wise LD matrices were calculated using imputed genotype data for a random set of 50,000 individuals from the unrelated European individuals in the UKB data. The LD matrices depicted were from the shrunk matrix estimator described in the main text and the basepair window width represents the distance from the first non-zero row correlation to the last non-zero row correlation for each variant. N.B. some elements within the window may be equal to zero and are not stored in the sparse matrix form lowering memory and improving computational efficiency.

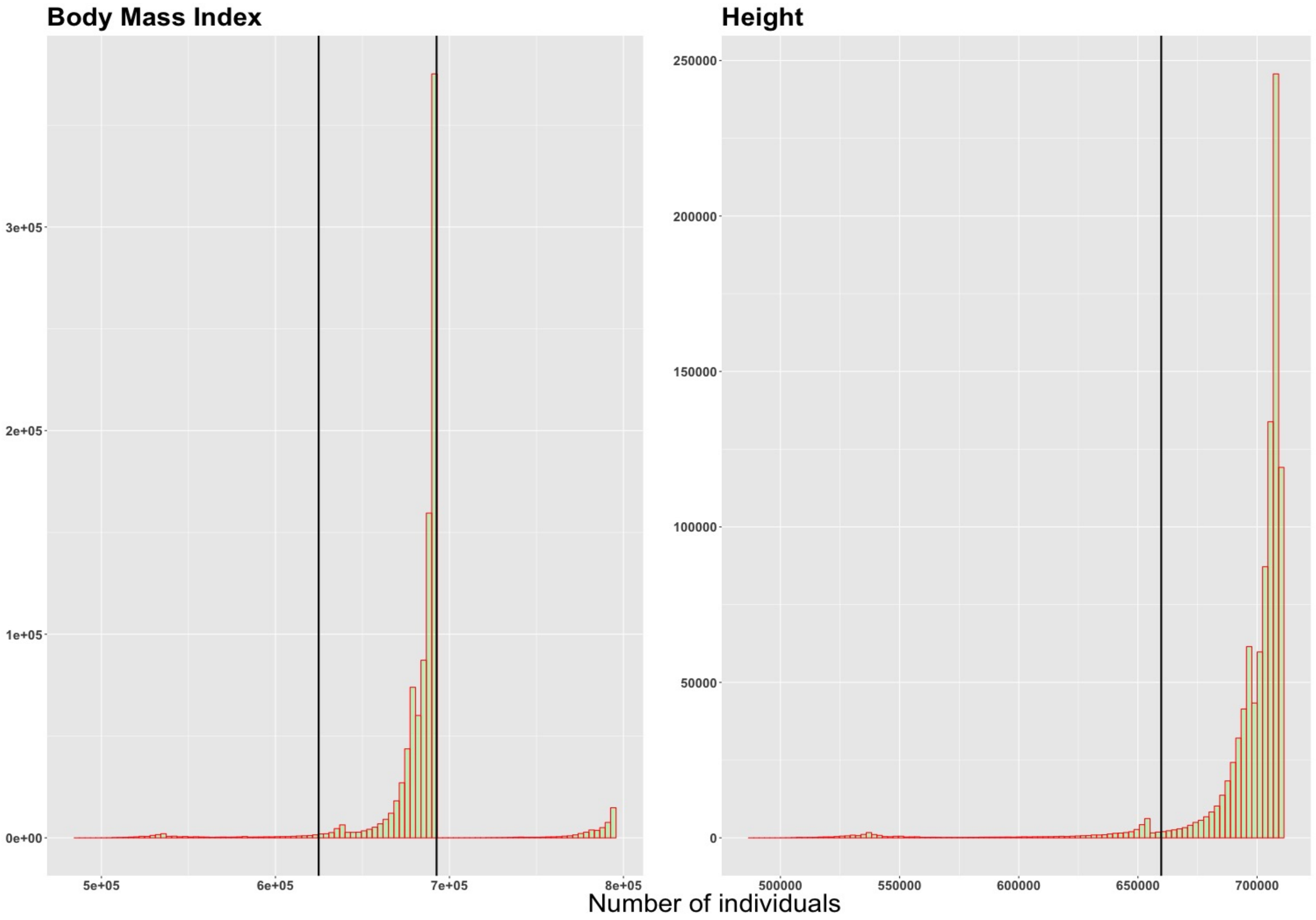

Supplementary Figure 23 Distribution and truncation of per-variant sample size from BMI and height summary statistics for 982,000 HapMap3 variants from Yengo *et al.*[2]. The 982,000 variants are those that overlap between the summary statistics made available from Yengo *et al.*[2] and the 1.09 M HM3 variants used in the simulation and cross-validation analyses. Vertical bars indicate the 0.025 and 0.95 percentiles for BMI and the 0.05 percentile for height. These truncations on n reduced the variant sets to 909,293 and 932,969 for BMI and height respectively. This truncation is required for model stability as the RSS and SBayesR models assume that the summary data were generated from the same set of individuals.

	Method	Prediction R^2	Null	Alternative	F-statistic	p-value	Partial R^2
HRS - BMI							
	P+T	0.1027	–	–	–	–	–
	BayesR	0.1251	P+T	P+T + BayesR	252.7	< 2.2e-16	0.030
	SBayesR-UKB	0.1323	BayesR	BayesR + SBayesR-UKB	77.3	< 2.2e-16	0.009
	LDpred	0.1326	SBayesR-UKB	SBayesR + LDpred	38.3	6.5e-10	0.005
	SBayesR 2.8 M	0.1343	LDpred	LDpred + SBayesR 2.8 M	99.4	< 2.2e-16	0.012
	RSS	0.1353	SBayesR 2.8 M	SBayesR 2.8 M + RSS	93.8	< 2.2e-16	0.011
	SBayesR	0.1357	RSS	RSS + SBayesR	11.8	5.8e-4	0.002
HRS - Height							
	P+T	0.2676	–	–	–	–	–
	LDPred	0.2973	P+T	P+T + LDPred	574.0	< 2.2e-16	0.065
	BayesR	0.3150	LDPred	LDPred + BayesR	448.0	< 2.2e-16	0.052
	RSS	0.3166	BayesR	BayesR + RSS	264.1	< 2.2e-16	0.031
	SBayesR-UKB	0.3213	RSS	RSS + SBayesR-UKB	202.4	< 2.2e-16	0.024
	SBayesR	0.3217	SBayesR-UKB	SBayesR-UKB + SBayesR	152.6	< 2.2e-16	0.018
	SBayesR 2.8 M	0.3416	SBayesR	SBayesR + SBayesR 2.8 M	431.7	< 2.2e-16	0.050
ESTB - BMI							
	P+T	0.0909	–	–	–	–	–
	BayesR	0.1095	P+T	P+T + BayesR*	870.0	< 2.2e-16	0.026
	SBayesR-UKB	0.1155	BayesR	BayesR + SBayesR-UKB	259.2	< 2.2e-16	0.008
	LDpred	0.1200	SBayesR-UKB	SBayesR-UKB + LDpred	222.2	< 2.2e-16	0.007
	SBayesR 2.8 M	0.1175	LDpred	LDpred + SBayesR 2.8 M	265.4	< 2.2e-16	0.008
	RSS	0.1203	SBayesR 2.8 M	SBayesR 2.8 M + RSS	387.1	< 2.2e-16	0.012
	SBayesR	0.1224	RSS	RSS + SBayesR	86.0	< 2.2e-16	0.003
ESTB - Height							
	P+T	0.2719	–	–	–	–	–
	LDPred	0.3012	P+T	P+T + LDPred	2287.8	< 2.2e-16	0.066
	BayesR	0.3147	LDPred	LDPred + BayesR	1638.9	< 2.2e-16	0.048
	RSS	0.32003	BayesR*	BayesR + RSS	1109.8	< 2.2e-16	0.033
	SBayesR-UKB	0.32006	RSS	RSS + SBayesR-UKB	673.0	< 2.2e-16	0.020
	SBayesR	0.3261	SBayesR-UKB	SBayesR-UKB + SBayesR	749.8	< 2.2e-16	0.023
	SBayesR 2.8 M	0.3521	SBayesR	SBayesR + SBayesR 2.8 M	2016.5	< 2.2e-16	0.058

Supplementary Table 1 Summary of across-biobank predictions and testing of polygenic risk scores (PRSs) variance explained. This table supplements the results presented in main text Figure 4. Methods are ranked by prediction R^2 and two linear models are run: Model 1 - true phenotype on the lower ranked PRS (null); Model 2 - true phenotype on lower plus higher ranked PRS (alternative). ANOVA is used to compare the null versus alternative and the F-statistic and associated p-value are reported from the ANOVA run. The coefficient of partial determination (Partial R^2) is also reported for the null versus alternative and measures the proportional reduction in sums of squares after the higher ranked PRS is introduced into the linear model.

1. Supplementary Note 1 - Simulation study using chromosomes 21 and 22

1.1. Description

To initially investigate the performance of the SBayesR methodology, we simulated quantitative phenotypes using 30,122 HM3 variants from chromosomes 21 and 22 for a random subset of 100,000 individuals from the 348,580 unrelated Europeans in the version three UKB data set. The variants on chromosomes 21 and 22 were taken from the list of 1,365,446 HM3 SNPs, which included a final filter that excluded SNPs with MAC $\geqslant 5$ and pHWE $< 1 \times 10^{-5}$ and missingness > 0.05, in the UKB data set. Taking the overlap between the HM3 variants on these chromosomes and the 1000G genetic map downloaded from joepickrell/1000-genomes-genetic-maps left 30,122 variants. The 1000G genetic map is required for use in the LD matrix shrinkage estimator of Wen and Stephens[7]. The genetic map files contain interpolated map positions for the CEU population generated from the 1000G OMNI arrays. The shrinkage estimator of the LD matrix, shrinks the off-diagonal entries of the an LD correlation matrix toward zero and is required for the Regression with Summary Statistics (RSS) method[1].

Using these genotypes, three genetic architecture scenarios were generated under the multiple regression model $y_i = \sum_{j=1}^{p} w_{ij}\beta_j + \varepsilon_i$, where $w_{ij} = (x_{ij} - 2q_j)/\sqrt{2q_j(1-q_j)}$ with x_{ij} being the reference allele count for the ith individual at the jth SNP, q_j the allele frequency of the jth variant and ε_i was sampled from a normal distribution with mean 0 and variance $\text{Var}(\mathbf{W}\boldsymbol{\beta})(1/h^2_{SNP} - 1)$ such that $h^2_{SNP} = 0.1$ for each simulation replicate, which is larger than the contribution to the genome-wide SNP-based heritability (h^2_{SNP}) estimate for these chromosomes for most quantitative traits. For each scenario replicate, the length p of $\boldsymbol{\beta}$ was set to 1,500 causal variants, which is the approximate number of causal variants that are expected given the proportion of HM3 variants on chromosomes 21 and 22 and a trait with 50,000 genome-wide causal variants. The elements β_j of $\boldsymbol{\beta}$ were sampled from the following distributions: the first genetic architecture (GA1) contained two causal

variants of large effect explaining 3% and 2% of the phenotypic variance respectively and a polygenic tail of 1,498 causal variants sampled from a N(0, 0.05/1, 498) distribution such that the expected total genetic variance explained by all variants was 0.1. The second architecture (GA2) was simulated under a BayesR model with three sets of causal variants: the first contained 1,445 causal variants sampled from a $N(0, 0.0.06/1445)$ distribution, the second contained 50 causal variants sampled from a $N(0, 0.02/50)$ distribution and the third five causal variants sampled from $N(0, 0.02/5)$ distribution. The third architecture (GA3) contained 1,500 variants sampled from a $N(0, 0.1/1500)$ distribution. For each of the three genetic architecture scenarios, 10 simulation replicates were generated for the 100,000 individuals by taking a new sample of genetic effects from the above distributions.

We generated two independent tuning and validation genotype sets from the remaining 248,580 unrelated European individuals each containing 10,000 individuals. The tuning genotype data set is required for parameter tuning, for example, the p-value threshold when performing clumping and then p-value thresholding Tuning and validation phenotypes were generated using the effects generated from the training data. For each of the 10 simulation replicates in the three scenarios, simple linear regression for each variant was run using the PLINK 1.9 software[8] to generate summary statistics. All phenotypes were generated using the R programming language. For each of the simulation scenarios the following methods were applied: LDpred[9], RSS[1], summary BLUP (SBLUP)[10], LD clumping and then p-value thresholding (P+T) implemented in PLINK 1.9, individual data BayesR[6] and the summary data implementation of BayesR (SBayesR) implemented in the GCTB software. For h^2_{SNP} comparison we ran Haseman-Elston regression (HEreg) in the GCTA software[11–13]. LDSC was run using LD scores calculated from the 1000G Europeans provided by the software and h^2_{SNP} estimation performed. . The SBayesR and RSS methods require precomputed reference LD correlation matrices.

To assess the influence of LD data reference on prediction performance and parameter estimation, we generated LD correlation matrices for the 30,122 HM3 variants using genotypes from the 1000G, ARIC and UK10K cohorts and six random subsamples from the

UKB genotype data with 378 (UKB-378), 500 (UKB-500), 750 (UKB-750), 1000 (UKB-1K), 5,000 (UKB-5K) and 50,000 (UKB-50K) individuals. The sample size of 378 individuals was chosen to match that of the 1000G and the other six to investigate a value at which optimal SBayesR performance is reached. For each LD reference cohort chromosome-wise LD matrices i.e., all inter chromosomal LD is ignored, were built and the shrinkage estimator of the LD matrix[7] calculated using an efficient implementation in the GCTB software. The calculation of the shrunk LD matrix requires the effective population sample size, which we set to be 11,400 (as in Zhu and Stephens[1]), the sample size of the genetic map reference, which corresponds to the 183 individuals from the CEU cohort of the 1000G and the hard threshold on the shrinkage value, which we set to 10^{-3}. We further stored the shrunk LD matrix in sparse matrix format (ignoring matrix elements equal to 0) for efficient SBayesR computation. SBayesR was run for each of the simulation scenarios using each reference LD matrix.

The PLINK 1.9 software was used to calculate the estimate genetic values for each individual for all LD matrix cohorts and the prediction R^2, calculated via linear regression of the true simulated phenotype in the validation data set on that predicted values from SBayesR, used as a measure of prediction accuracy. The UKB-50K reference showed marginal improvements over the other cohorts in prediction accuracy in the validation data set and had the smallest upward bias in h^2_{SNP} estimation (Figure Supplementary Figure 1). We therefore selected this LD reference cohort for all methods. For LDpred, SBLUP and P+T, a separate genotype data set is required to be specified for LD correlation reference and utilisation within each method's program. This was set to be the same 50,000 individual genotype set used for SBayesR and RSS. Furthermore, the full LD matrix that incorporates inter-chromosomal LD information was generated using the full 100,000 individuals such that the individual data BayesR model could be compared with the SBayesR model run with the full LD matrix, which is expected to produce equivalent results.

For LDpred, we specified h^2_{SNP} to be equal to the true 0.1, specified the number of SNPs

on each side of the focal SNP for which LD should be adjusted to be 3,500, which equated to an approximate 10 megabase (MB) window, and calculated effects size estimates for all of the 10 fraction of non-zero effects pre-specified parameters, which included LDpred-inf, 1, 0.3, 0.1, 0.03, 0.01, 0.003, 0.001, 0.0003, and 0.0001. A larger LD window size than recommended was chosen because of the large effects simulated, it was computationally feasible in this small simulation and to be comparable with the LD references used for SBayesR and RSS. For RSS, analyses were performed for each chromosome to limit the computational burden of running these analyses using MATLAB, as in Zhu and Stephens[1]. For each chromosome, the RSS-BSLMM model was run for 2 million MCMC iterations with 1 million as burn in and a thinning rate of 1 in 100 to arrive at 10,000 posterior samples for each of the model parameters. For each chromosome, the posterior mean over posterior samples for the SNP effects and h^2_{SNP} estimates was used. The chromosome wise h^2_{SNP} estimates were then summed to get the total estimate. For SBLUP, we used the GCTA software implementation, which required the specification of the $\lambda = m(1/h^2_{SNP} - 1)$ parameter, which was calculated using $h^2_{SNP} = 0.1$ and $m = 30,122$ and the LD correlation window size specification was set to 10 MB. For P+T, we used the PLINK 1.9 software to clump the GWAS summary statistics discarding variants within 1 MB (using 10 MB gave very similar results) of and in LD $R^2 > 0.1$ with the most associated SNP in the region. Using these clumped results, we generated PRSs for sets of SNPs at the following p-value thresholds: 5×10^{-8}, 1×10^{-6}, 1×10^{-4}, 0.001, 0.01, 0.05, 0.1, 0.2, 0.5, and 1.0. BayesR was run using a mixture of four normal distributions model with distribution variance weights γ = (0, 10^{-4}, 10^{-3}, 10^{-2})′. BayesR was run for 4,000 iterations with 2,000 taken as burn in and a thinning rate of 1 in 10. The posterior mean of the effects and the proportion of variance explained over the 200 posterior samples was taken as the parameter estimate for each scenario replicate. For SBayesR the MCMC chain was run for 4,000 iterations with 2,000 taken as burn in and a thinning rate of 1 in 10 and run with four distributions and variance weights γ = (0, 0.01, 0.1, 1)′. HEreg requires a genetic relatedness matrix, which was built from 30,122 HM3 variants from chromosomes 21 and 22 using the GCTA

software.

To assess prediction accuracy, the polygenic risk score (PRS) for each individual was calculated using the genotype data from the 10,000 individual tuning and validations data sets and genetic effect estimates from each method. Tuning was performed for LDpred and P+T where for each simulation replicate the prediction accuracy was assessed for each of the pre-specified fraction of non-zero effects parameters for LDpred and the p-value thresholds for P+T. The parameter that gave the optimal prediction R^2 in the tuning data set was then used for calculating the PRS in the validation data set. SNP effects from BayesR and SBayesR were estimated using scaled genotypes and thus each variant's effect was divided by $\sqrt{2q_j(1-q_j)}$, where q_j is the allele frequency from the validation cohort of the jth variant, before PLINK scoring was performed. The PLINK 1.9 software was used to perform the EGV calculation for all methods and the prediction R^2 calculated via linear regression of the true simulated phenotype on that estimated from each method used as a measure of prediction accuracy.

1.2. Results

The choice of LD matrix reference cohort for use in SBayesR analysis led to differences in absolute prediction accuracy and bias in h^2_{SNP} estimation (Supplementary Figure 1 and Supplementary Figure 2). The UKB-378 or 1000G cohort showed the poorest prediction accuracy and upward bias in on mean h^2_{SNP} estimates. Improvement absolute prediction accuracy and bias in h^2_{SNP} when the random subsample from the UKB was increase from 378 to 1000 individuals. The ARIC and UK10K LD reference cohorts showed similar on mean prediction R^2 and bias in h^2_{SNP} estimation as the UKB-50K and UKB-5K cohorts. The results for smaller references suggest that the larger sampling variance for the "non-true" LD matrix entries of these LD matrices can influence the SBayesR approximate Gibbs sampling algorithm with 3-5,000 individuals appearing a minimum for optimal SBayesR results although reasonable results can be obtained with smaller references. However, overall the UKB-50K cohort showed the maximum prediction accuracy and smallest upward bias in h^2_{SNP} estimation across all scenarios and thus was chosen as the

LD reference cohort for all analyses.

Across the three simulation scenarios we observed that the individual level analysis using BayesR, or SBayesR with the full LD matrix, gave the highest mean validation data set prediction R^2 (Supplementary Figure 3). Marginal differences in prediction accuracy variance were observed between BayesR and SayesR with the full LD matrix for the GA1 and GA2 simulation scenarios (Supplementary Figure 3). These differences could be caused by the different implementation of the methodology where BayesR results are generated from the program written in Moser et al.[6]. Furthermore, in the SBayesR method we allow each SNP to have a different residual variance, which could be slightly different to individual data results because of the small differences in per-SNP sample size and estimation variance. The rounding of the summary statistics and subsequent model reconstruction from these rounded values could also contribute. These sources are the likely cause of the marginal differences in variance (in scenarios GA1 and GA2) across replicates between the two methods. We highlight that the prediction accuracy means are exactly the same between these two methods in all scenarios.

The relative difference between the individual data BayesR model mean prediction accuracy and the highest performing summary statistics method, SBayesR, ranged from 1.2% to 3.9% (Supplementary Figure 3). P+T showed the lowest on mean prediction accuracy across scenarios but showed similar mean prediction accuracies to the LDpred infinitesimal model and SBLUP for scenario one, which contains variants of very large effect and a polygenic tail. SBayesR showed substantial improvement in prediction accuracy relative to other summary statistics methodologies particularly in scenario one, with RSS showing the closest prediction R^2 compared to SBayesR in all scenarios. RSS outperformed SBLUP and LDpred-inf in all scenarios but showed a smaller relative improvement in prediction R^2 as the simulated traits had fewer large effects. SBLUP outperformed LDpred-inf in each of the simulation scenarios, which is the most similar LDpred model to SBLUP (Supplementary Figure 3). Overall, on mean prediction R^2 improvement ranged from 1.3% to 7.9% when comparing SBayesR with the best alternative summary statistic method (RSS) across all

scenarios (Supplementary Figure 3).

Across all simulation scenarios all methods showed minimal bias in h^2_{SNP} estimation (Supplementary Figure 4). The deflation of the LDSC estimate across scenarios should be interpreted with caution as it is a likely result of the use of the small number of variants in this simulation. Simulations (not shown) using chromosomes 1-3 and the GA3 simulation scenario show unbiased LDSC estimates. Overall SBayesR using the full LD matrix showed the smallest bias, with HEreg showing the largest bias in scenario one with the bias diminishing as the scenarios became more similar to the infinitesimal model. RSS showed a downward bias in GA1 and was unbiased for GA2 and GA3. SBayesR maintained a marginal upward bias across all simulation scenarios and a maximum relative upward on mean bias of 3% in GA2 and GA3 (Supplementary Figure 4).

2. Supplementary Note 2 - Bayesian multiple regression

The starting point is the multiple linear model of the form

$$\mathbf{y} = \mathbf{X}\boldsymbol{\beta} + \boldsymbol{\varepsilon}, \tag{1}$$

where $\mathbf{y}$ is an $n \times 1$ vector (centred) of trait phenotypes, $\mathbf{X}$ is an $n \times p$ matrix of genotype covariates initially coded 0, 1, 2 representing the number of copies of a reference allele at each marker, and we consider that the columns of $\mathbf{X}$ can either be centred or centred and scaled. The vector $\boldsymbol{\beta}$ is a $p \times 1$ vector of random partial regression coefficients of the p SNPs (marker effects) and $\boldsymbol{\varepsilon}$ is a vector $(n \times 1)$ of residuals.

We wish to optimise the parameters of the stated linear model using Bayesian posterior inference, which requires the specification of prior distributions for $\boldsymbol{\beta}$ and $\boldsymbol{\varepsilon}$. We assume that the error term $\boldsymbol{\varepsilon}|\sigma^2_\varepsilon \sim \text{MVN}(\mathbf{0}, \mathbf{R}\sigma^2_\varepsilon)$, where MVN denotes the multivariate normal distribution, $\mathbf{0}$ is a column vector of zeroes of length n, and $\mathbf{R}$ is a covariance matrix, which is assumed here to be a diagonal matrix of ones. The parameter σ^2_ε is treated as an unknown with a scaled inverse chi-square distribution prior with scale parameter s^2_ε and degrees of freedom ν_ε.

Members of the Bayesian alphabet for genomic selection including BayesA and BayesB[14], BayesC and BayesCπ[15], BayesR[6,16], BSLMM[17], BayesS[18] among others, differ largely in the prior used for $\boldsymbol{\beta}$. In this work, we will focus on the BayesR model, which assumes that β_j comes from a finite mixture of normals distribution, which includes a point mass at zero. This prior is motivated by the capacity of the mixture distribution to be flexible and thus model a diverse set of underlying genetic effect distributions.

Inferences on marker associations are based on the posterior distribution of the marker effects $f(\boldsymbol{\beta}|\mathbf{y})$. Closed form expressions are not available for making inferences from $f(\boldsymbol{\beta}|\mathbf{y})$ and instead they are drawn from the posterior of interest. The following derivation describes a similar Markov chain Monte Carlo (MCMC) algorithm as in Habier *et al.*[15], which is discussed in detail in Fernando and Garrick[19].

Let $\boldsymbol{\theta} = (\boldsymbol{\beta}', \boldsymbol{\pi}', \sigma^2_\beta, \sigma^2_\varepsilon)'$ denote all the unknowns in the model including the random marker effects, mixing proportions of the mixture of normals, the variance of the marker effects, and the residual variance. For each model parameter, we draw posterior samples using the single site Gibbs sampler, which draws samples for each element i of the vector $\boldsymbol{\theta}$ from its full conditional posterior: $f(\theta_i|\boldsymbol{\theta}_{-i}, \mathbf{y})$. The full conditional can be expressed as

$$f(\theta_i|\boldsymbol{\theta}_{-i}, y) \propto f(\theta_i, \boldsymbol{\theta}_{-i}, \mathbf{y}). \tag{2}$$

The joint density in Supplementary Equation (2) can be written as

$$f(\theta_i, \boldsymbol{\theta}_{-i}, \mathbf{y}) = f(\mathbf{y}|\boldsymbol{\theta}) f(\theta_i) f(\boldsymbol{\theta}_{-i}),$$

where $f(\mathbf{y}|\boldsymbol{\theta})$ is the density function of the conditional distribution of $\mathbf{y}|\boldsymbol{\theta}$, and $f(\theta_i)$ and $f(\boldsymbol{\theta}_{-i})$ are the densities of the prior distributions of θ_i and θ_{-i}. Ignoring factors that are constant with respect to θ_i gives the kernel of the full-conditional posterior for each parameter of interest, which we will derive for each element of $\boldsymbol{\theta}$.

The conditional distribution of $\mathbf{y}$ given all the unknowns is MVN with expectation $\mathbf{X}\boldsymbol{\beta}$

and covariance matrix $\mathbf{R}\sigma_e^2$. The MVN density is thus

$$f(\mathbf{y}|\boldsymbol{\theta}) = (2\pi\sigma_e^2)^{-n/2} \exp\left[-\frac{(\mathbf{y}-\mathbf{X}\boldsymbol{\beta})'(\mathbf{y}-\mathbf{X}\boldsymbol{\beta})}{2\sigma_e^2}\right]. \tag{3}$$

Formally, under the BayesR model we assume the following prior on the genetic effects

$$\beta_j|\boldsymbol{\pi},\sigma_\beta^2 = \begin{cases} 0 & \text{with probability } \pi_1, \\ \sim N(0,\gamma_2\sigma_\beta^2) & \text{with probability } \pi_2, \\ \vdots & \\ \sim N(0,\gamma_C\sigma_\beta^2) & \text{with probability} 1-\sum_{c=1}^{C-1}\pi_c, \end{cases}$$

where C denotes the maximum number of components in the finite mixture model, which is prespecified. The γ_c coefficients are prespecified and constrain how the common marker effect variance σ_β^2 scales in each distribution. For example it is common in BayesR to assume $C = 4$ such that $\boldsymbol{\gamma} = (\gamma_1,\gamma_2,\gamma_3,\gamma_4)' = (0, 0.0001, 0.001, 0.01)'$ representing a class of effects with no effect and three further classes of small, medium and large effects. Under this prior assumption, we derive the MCMC Gibbs sampling routine for sampling of the key model parameters $\boldsymbol{\theta} = (\boldsymbol{\beta}',\boldsymbol{\pi}',\sigma_\beta^2,\sigma_\varepsilon^2)'$ from their full conditional distributions.

We introduce the dummy variable δ_j which is a random variable that takes values $1, 2, \ldots, C$ depending on which mixture distribution marker j is sampled in. The prior distribution for the marker effects conditional on the marker effect variance σ_β^2 and mixture class is

$$f(\boldsymbol{\beta}|\boldsymbol{\delta} = c, \gamma_c\sigma_\beta^2) = \prod_{j=1}^{k_c}(2\pi\gamma_c\sigma_\beta^2)^{-1/2}\exp\left[-\frac{\beta_j^2}{2\gamma_c\sigma_\beta^2}\right],$$

which represents the product over those markers sampled in class c denoted k_c. We assume that the prior for σ_β^2 is a scaled inverse chi-square distribution with density

$$f(\sigma_\beta^2;\nu_\beta,S_\beta^2) = \frac{(S_\beta^2\nu_\beta/2)^{\nu_\beta/2}}{\Gamma(\nu_\beta/2)}\frac{\exp(-\nu_\beta S_\beta^2/2\sigma_\beta^2)}{(\sigma_\beta^2)^{1+\nu_\beta/2}},$$

where S_β^2 and ν_β are the scale parameter and degrees of freedom respectively. As stated above the residual variance σ_ε^2 is assumed to have scaled inverse chi-square distribution prior with distribution

$$f(\sigma_\varepsilon^2; \nu_\varepsilon, S_\varepsilon^2) = \frac{(S_\varepsilon^2 \nu_\varepsilon/2)^{\nu_\varepsilon/2}}{\Gamma(\nu_\varepsilon/2)} \frac{\exp(-\nu_\varepsilon S_\varepsilon^2/2\sigma_\varepsilon^2)}{(\sigma_\varepsilon^2)^{1+\nu_\varepsilon/2}}.$$

The full conditional posterior of β_j is proportional to the product of the likelihood, the prior distribution for β_j, and the prior distributions of the variances. The variances don't contain β_j and nor do the other components of the product for the prior for β_j. Therefore, the full conditional for β_j can be written as

$$f(\beta_j|\delta_j = c, \boldsymbol{\theta}_{-\beta_j}, \mathbf{y}) \propto \exp\left[-\frac{(\mathbf{y}-\mathbf{X}\boldsymbol{\beta})'(\mathbf{y}-\mathbf{X}\boldsymbol{\beta})}{2\sigma_e^2}\right] \exp\left[-\frac{\beta_j^2}{2\gamma_c\sigma_\beta^2}\right].$$

We define $\mathbf{w} = \mathbf{y} - \sum_{k\neq j} \mathbf{x}_k\beta_k$ to be the vector of trait phenotypes corrected for all effects other than that being sampled. Given this we can write

$$\begin{aligned} f(\beta_j|\delta_j = c, \boldsymbol{\theta}_{-\beta_j}, \mathbf{y}) &\propto \exp\left\{-\frac{1}{2\sigma_e^2}\left[(\mathbf{w}-\mathbf{x}_j\beta_j)'(\mathbf{w}-\mathbf{x}_j\beta_j) + \frac{\beta_j^2\sigma_e^2}{2\gamma_c\sigma_\beta^2}\right]\right\} \\ &\propto \exp\left[-\frac{1}{2\sigma_e^2}\left(\mathbf{w}'\mathbf{w} - 2\mathbf{x}_j'\mathbf{w}\beta_j + \mathbf{x}_j'\mathbf{x}_j\beta_j^2 + \frac{\beta_j^2\sigma_e^2}{\sigma_\beta^2}\right)\right]. \end{aligned} \tag{4}$$

We can complete the square with respect to β_j in Supplementary Equation (4) to obtain

$$f(\beta_j|\delta_j = c, \boldsymbol{\theta}_{-\beta_j}, \mathbf{y}) \propto \exp\left[-\frac{1}{2\sigma_e^2}\left(\mathbf{w}'\mathbf{w} - l_{jc}\widehat{\beta}_j^2 + l_{jc}(\beta_j - \widehat{\beta}_j)^2\right)\right],$$

where $l_{jc} = \mathbf{x}_j'\mathbf{x}_j + \sigma_e^2/(\gamma_c\sigma_\beta^2)$ is the left hand side of the well known mixed model equations (MME)[20] for β_j, $\widehat{\beta}_j = \mathbf{x}_j'\mathbf{w}/l_{jc}$, and $\mathbf{x}_j'\mathbf{w}$ is the right hand side of the MME. Dropping terms that are free from β_j the full conditional becomes

$$f(\beta_j|\delta_j = c, \boldsymbol{\theta}_{-\beta_j}, \mathbf{y}) \propto \exp\left[-\frac{1}{2}\frac{(\beta_j - \widehat{\beta}_j)^2}{\frac{\sigma_e^2}{l_{jc}}}\right].$$

This can be seen to be the kernel of the normal distribution and within each iteration of the Gibbs sampler we sample the genetic effect from a normal distribution with mean $\widehat{\beta}_j$ and variance σ_e^2/l_{jc}.

In BayesR the prior assumption is that the marker effects have IID Gaussian mixture distributions, with a point mass at zero with probability π_1, a univariate normal distribution with variance $\gamma_2\sigma_\beta^2$ with probability π_2, a univariate normal distribution with variance $\gamma_3\sigma_\beta^2$ with probability π_3 etc. up to a univariate normal distribution with variance $\gamma_C\sigma_\beta^2$ with probability π_C , such that $\pi_C = 1 - \sum_{c=1}^{C-1} \pi_c$, where C here denotes the maximum number of components in the finite mixture model. The vector $\boldsymbol{\pi} = (\pi_1, \pi_2, \ldots, \pi_C)'$ is treated as an unknown and is assumed to have a Dirichlet prior, which is the extension of the concept in BayesCπ[15] that the π is treated as an unknown with a uniform prior. BayesR classically assumes that there are $C = 4$ classes but C can be chosen to be arbitrarily large with some scaling (not necessarily an exponential function) of each of the variance components assumed.

To derive the posterior update for $\boldsymbol{\pi}$, we treat the indicator variables δ_j as a random variable that takes values $1, 2, 3, \ldots, C$ depending on which class marker j is sampled in. Therefore, δ_j can be modelled as a categorical random variable, which implies that

$$f(\delta_j|\boldsymbol{\pi}) = \prod_{c=1}^{C} \pi_c^{[\delta_j=c]} \quad ,$$

where $[\delta_j = c]$ evaluates to 1 if $\delta_j = c$ and 0 otherwise. Therefore,

$$f(\delta|\boldsymbol{\pi}) = \prod_{j=1}^{p}\prod_{c=1}^{C} \pi_c^{[\delta_j=c]}.$$

The Dirichlet distribution is the conjugate prior distribution of the categorical distribution. Given, δ_j has a categorical distribution if we assume that $\boldsymbol{\pi}$ has a $Dirichlet(1, 1, \ldots, 1)$ prior, which assumes that the prior probability of a SNP being in any distribution is the same, then the posterior distribution of δ_j is also Dirichlet. Then, more generally in our setting $\boldsymbol{\alpha} = (\alpha_1, \ldots, \alpha_c, \ldots, \alpha_C)$ where $\alpha_c = 1$ and we can write our prior as $\boldsymbol{\pi}|\boldsymbol{\alpha} \sim Dirichlet(C, \boldsymbol{\alpha})$.

Given an initial vector $\boldsymbol{\pi}$ then $\delta|\boldsymbol{\pi} \sim Categorical(C, \boldsymbol{\pi})$. From the form of the Dirichlet distribution we have

$$f(\pi_1, ..., \pi_C; \alpha_1, \ldots, \alpha_C) = \frac{1}{B(\boldsymbol{\alpha})} \prod_{c=1}^{C} \pi_c^{\alpha_c - 1}.$$

As always we can ignore the normalising constant and look at

$$f(\boldsymbol{\pi}|\boldsymbol{\delta}, \boldsymbol{\alpha}) \propto f(\boldsymbol{\delta}|\boldsymbol{\pi}) f(\boldsymbol{\pi}|\boldsymbol{\alpha}) = \prod_{j=1}^{p} \prod_{c=1}^{C} \pi_c^{[\delta_j = c]} \prod_{c=1}^{C} \pi_c^{\alpha_c - 1} = \prod_{j=1}^{p} \prod_{c=1}^{C} \pi_i^{[\delta_j = c] + \alpha_c - 1},$$

which is the kernel of a $Dirichlet(C, \mathbf{c} + \boldsymbol{\alpha})$, where $\mathbf{c}$ is a vector of length C with the count of the number of variants in each class and $\mathbf{c} + \boldsymbol{\alpha}$ is a vector with elements $(c_1 + \alpha_1, c_2 + \alpha_2, \ldots, c_C + \alpha_C)$. Therefore, in the Gibbs sampler we sample $\boldsymbol{\pi}$ from a $Dirichlet(C, \mathbf{c} + \boldsymbol{\alpha})$ conditional on the number of variants sampled in each of the C mixture classes.

[16] do not sample the marker effect variance σ_β^2 but instead scale and centre the genotypes and equate the genetic variance $\sigma_g^2 = m\sigma_\beta^2$, where m is the number of causal loci[21] and substitute a pre-estimated value of σ_g^2, from a previous h_{SNP}^2 study.[6] also equate the genetic variance with the marker effect variance and sample σ_g^2 from a scaled inverse chi-square distribution with parameters $\nu_0 + m_g$ and $\frac{m_g \sum_{j=1}^{p} \beta_j^2 + \nu_0 S_0^2}{\nu_0 + m_g}$, where m_g is the number of SNPs included in the current model. Moser *et al.*[6] specify prior values of ν_0 and S_0^2 are to be -2 and 0, which are proposed to lead to a uninformative prior. For polygenic traits this is likely to be reasonable but a more general hypothesis is to not to connect the genetic variance with the marker effect variance under this assumption.

To derive a new update, we note that the common marker effect variance is only present in the normal density functions of β_j when $\delta_j \neq 1$ and its own prior. Therefore,

$$f(\boldsymbol{\beta}|\sigma_\beta^2, \boldsymbol{\delta}) = \prod_{j=1}^{q} \phi(\beta_j; 0, \gamma_{\delta_j} \sigma_\beta^2),$$

where $\boldsymbol{\delta} = (\delta_2, \ldots, \delta_p)$, $\delta_j \in (1, 2, \ldots, C)$, ϕ is the normal probability density function and

the number of non-zero effects in the model $q = |\boldsymbol{\beta}_{-\beta_j:\delta_j=1}|$. Given this

$$
\begin{aligned}
f(\sigma^2_\beta; \nu_\beta, S^2_\beta) f(\boldsymbol{\beta}|\sigma^2_\beta, \boldsymbol{\delta}) &= \frac{(S^2_\beta \nu_\beta/2)^{\nu_\beta/2}}{\Gamma(\nu_\beta/2)} \frac{\exp(-\nu_\beta S^2_\beta/\sigma^2_\beta)}{(\sigma^2_\beta)^{1+\nu_\beta/2}} \prod_{j=1}^{q} (2\pi\gamma_{\delta_j}\sigma^2_\beta)^{-1/2} \exp\left[-\frac{\beta_j^2}{2\gamma_{\delta_j}\sigma^2_\beta}\right] \\
&= \frac{(S^2_\beta \nu_\beta/2)^{\nu_\beta/2}}{\Gamma(\nu_\beta/2)} \frac{\exp(-\nu_\beta S^2_\beta/2\sigma^2_\beta)}{(\sigma^2_\beta)^{1+\nu_\beta/2}} \gamma_{\delta_2}^{-c_2/2} \ldots \gamma_{\delta_C}^{-c_C/2} (2\pi\sigma^2_\beta)^{-q/2} \exp\left[-\frac{1}{2\sigma^2_\beta} \sum_{j=1}^{q} \frac{\beta_j^2}{\gamma_{\delta_j}}\right],
\end{aligned}
$$

where $(c_2, \ldots, c_C)$ are the number of variants in each of the non-zero classes. Retaining only those elements that contain σ^2_β

$$
\begin{aligned}
&\propto \frac{\exp(-\nu_\beta S^2_\beta/2\sigma^2_\beta)}{(\sigma^2_\beta)^{1+\nu_\beta/2}} (\sigma^2_\beta)^{-q/2} \exp\left[-\frac{\sum_{j=1}^{q} \beta_j^2}{2\gamma_{\delta_j}\sigma^2_\beta}\right] \\
&\propto \exp\left[-\frac{1}{2\sigma^2_\beta}\left(\nu_\beta S^2_\beta + \sum_{j=1}^{q} \frac{\beta_j^2}{\gamma_{\delta_j}}\right)\right] (\sigma^2_\beta)^{-1-\nu_\beta/2-q/2},
\end{aligned}
$$

which is the kernel of a scale inverse chi-squared distribution with degrees of freedom $\nu_\beta + q$, where q is the number of non-zero markers in the model. The scale parameter can be determined by letting $\widetilde{\nu}_\beta = \nu_\beta + q$. The expression inside must be equal to $\widetilde{\nu}_\beta \widetilde{S}^2_\beta = \nu_\beta S^2_\beta + \sum_{j=1}^{q} \frac{\beta_j^2}{\gamma_{\delta_j}}$. The new scale parameter is thus now

$$
\widetilde{S}^2_\beta = \frac{\nu_\beta S^2_\beta + \sum_{j=1}^{q} \frac{\beta_j^2}{\gamma_{\delta_j}}}{\nu_\beta + q}.
$$

This is only equivalent to that presented in Moser *et al.*[6] when each γ_{δ_j} is equal to $1/q$.

2.1. Joint sampling of δ_j and β_j

We employ a similar strategy as[18] to jointly sample δ_j and β_j by first sampling δ_j unconditional on β_j and then sample β_j conditional on δ_j. Mathematically

$$
f(\beta_j, \delta_i|\boldsymbol{\theta}_{-\beta_j,\delta_j}, \mathbf{y}) = f(\beta_j|\delta_j, \boldsymbol{\theta}_{-\beta_j}, \mathbf{y}) f(\delta_j|\boldsymbol{\theta}_{-\delta_j}, \mathbf{y}),
$$

and then sample β_j from $f(\beta_j|\delta_j = c, \boldsymbol{\theta}_{-\beta_j}, \mathbf{y})$. The categorical random variable δ_j appears in the likelihood and in its own prior

$$f(\delta_j|\boldsymbol{\pi}) = \prod_{c=1}^{C} \pi_j^{[\delta_j=c]}.$$

Samples can be drawn from this categorical distribution by calculating the membership probabilities

$$\mathbb{P}(\delta_j = c|\boldsymbol{\theta}_{-\delta_j}, \mathbf{y}) = \frac{f(\mathbf{w}|\delta_j = c, \boldsymbol{\theta}, \mathbf{y})\mathbb{P}(\delta_j = c)}{\sum_{c=1}^{C} f(\mathbf{w}|\delta_j = c, \boldsymbol{\theta}, \mathbf{y})\mathbb{P}(\delta_j = c)},$$

and then use the sampling routine for a categorical distribution once the probabilities are known. If we would like to use this then if $\delta_j \neq 1$ then we need to integrate out β_j from $f_j(\mathbf{w}|\delta_j = c, \boldsymbol{\theta}, \mathbf{y})$, which requires the following integral

$$\begin{aligned} f(\mathbf{w}|\delta_j = c, \boldsymbol{\theta}, \mathbf{y}) &= \int f(\mathbf{w}|\beta_j, \sigma_\varepsilon^2) f(\beta_j|\delta_j, \sigma_\alpha^2) d\beta_j \\ &= \int (2\pi\sigma_\varepsilon^2)^{-n/2} \exp\left[-\frac{(\mathbf{w} - \mathbf{x}_j\beta_j)'(\mathbf{w} - \mathbf{x}_j\beta_j)}{2\sigma_\varepsilon^2}\right] (2\pi\gamma_c\sigma_\beta^2)^{-1/2} \exp\left[-\frac{\beta_j^2}{2\gamma_c\sigma_\beta^2}\right] d\beta_j. \end{aligned}$$

Expanding the product terms and combining the exponential terms we have

$$f(\mathbf{w}|\delta_j = c, \boldsymbol{\theta}, \mathbf{y}) = \int (2\pi\gamma_c\sigma_\beta^2)^{-1/2} (2\pi\sigma^2)^{-n/2} \exp\left[-\frac{1}{2\sigma_\varepsilon^2}\left(\mathbf{w}'\mathbf{w} - 2\mathbf{x}_j'\mathbf{w}\beta_j + \mathbf{x}_j'\mathbf{x}_j\beta_j^2 + \frac{\beta_j^2\sigma_\varepsilon^2}{\gamma_c\sigma_\beta^2}\right)\right] d\beta_j.$$

We let $l_{jc} = \mathbf{x}_j'\mathbf{x}_j + \sigma_e^2/(\gamma_c\sigma_\beta^2)$ and thus

$$= \int (2\pi\gamma_c\sigma_\beta^2)^{-1/2} (2\pi\sigma_\varepsilon^2)^{-n/2} \exp\left[-\frac{1}{2\sigma_\varepsilon^2}\left(\mathbf{w}'\mathbf{w} - 2\mathbf{x}_j'\mathbf{w}\beta_j + \beta_j^2 l_{jc}\right)\right] d\beta_j.$$

Letting $\widehat{\beta}_j = \mathbf{x}_j'\mathbf{w}/l_{jc}$ we again look to complete the square and take out from the integral those elements that do not involve β_j

$$\begin{aligned}
&= \int (2\pi\gamma_c\sigma_\beta^2)^{-1/2}(2\pi\sigma_\varepsilon^2)^{-n/2}\exp\left[-\frac{1}{2\sigma_e^2}\left(\mathbf{w}'\mathbf{w} - 2\widehat{\beta}_j l_{jc}\beta_j + \beta_j^2 l_{jc} + \widehat{\beta}_j^2 l_{jc} - \widehat{\beta}_i^2 l_{jc}\right)\right] d\beta_j \\
&= (2\pi\frac{\sigma_\varepsilon^2}{l_j})^{1/2}(2\pi\gamma_c\sigma_\beta^2)^{-1/2}(2\pi\sigma_\varepsilon^2)^{-n/2}\exp\left[-\frac{1}{2\sigma_\varepsilon^2}\left(\mathbf{w}'\mathbf{w} - l_{jc}\widehat{\beta}_j^2\right)\right] \times \\
&\quad \int (2\pi\frac{\sigma_\varepsilon^2}{l_{jc}})^{-1/2}\exp\left[-\frac{1}{2\frac{\sigma_\varepsilon^2}{l_{jc}}}(\beta_j - \widehat{\beta}_j)^2\right] d\beta_j.
\end{aligned}$$

The integral component is now a normal distribution and thus integrates to 1. The term left over after cleaning is

$$f(\mathbf{w}|\delta_j = c, \boldsymbol{\theta}, \mathbf{y}) = \left(\frac{\gamma_{\delta_j}\sigma_\beta^2 l_j}{\sigma_\varepsilon^2}\right)^{-1/2}(2\pi\sigma_\varepsilon^2)^{-n/2}\exp\left[-\frac{1}{2\sigma_e^2}\left(\mathbf{w}'\mathbf{w} - l_{jc}\widehat{\beta}_j^2\right)\right].$$

Our goal was to derive a form for $f(\mathbf{w}|\delta_j = c, \boldsymbol{\theta}, \mathbf{y})$ that was independent of β_j for use in the probability calculations in

$$\mathbb{P}(\delta_j = c|\boldsymbol{\theta}_{-\delta_j}, \mathbf{y}) = \frac{f(\mathbf{w}|\delta_j = c, \boldsymbol{\theta}, \mathbf{y})\mathbb{P}(\delta_j = c)}{\sum_{c=1}^{C} f(\mathbf{w}|\delta_j = c, \boldsymbol{\theta}, \mathbf{y})\mathbb{P}(\delta_j = c)}.$$

We only need to calculate $C - 1$ of these as the Cth probability is $1 - \sum_{c=1}^{C}\mathbb{P}(\delta_j = c|\boldsymbol{\theta}, \mathbf{y})$. We calculate the probabilities for multiple terms similarly to the following example

$$\begin{aligned}
\frac{f(\mathbf{w}|\delta_j = 1, \boldsymbol{\theta}, \mathbf{y})}{f(\mathbf{w}|\delta_j = 2, \boldsymbol{\theta}), \mathbf{y})} &= \frac{f(\mathbf{w}|\delta_j = 1, \boldsymbol{\theta}, \mathbf{y})}{\int f(\mathbf{w}|\delta_j = 2, \beta_j, \boldsymbol{\theta}, \mathbf{y})f(\beta_j|\delta_j, \sigma_\beta^2)d\beta_j} \\
&= \frac{(2\pi\sigma_\varepsilon^2)^{-n/2}\exp\left[-\frac{1}{2\sigma_\varepsilon^2}\left(\mathbf{w}'\mathbf{w}\right)\right]}{\left(\frac{\gamma_2\sigma_\beta^2 l_{j2}}{\sigma_\varepsilon^2}\right)^{-1/2}(2\pi\sigma_\varepsilon^2)^{-n/2}\exp\left[-\frac{1}{2\sigma_\varepsilon^2}\left(\mathbf{w}'\mathbf{w} - l_{j2}\widehat{\beta}_j^2\right)\right]} \\
&= \left(\frac{\gamma_2\sigma_\beta^2 l_{j2}}{\sigma_\varepsilon^2}\right)^{1/2}\exp\left[-\frac{1}{2\sigma_\varepsilon^2}(\mathbf{w}'\mathbf{w}) + \frac{1}{2\sigma_\varepsilon^2}(\mathbf{w}'\mathbf{w}) - \frac{1}{2\sigma_\varepsilon^2}(l_{j2}\widehat{\beta}_j^2)\right] \\
&= \left(\frac{\gamma_2\sigma_\beta^2 l_{j2}}{\sigma_\varepsilon^2}\right)^{1/2}\exp\left[-\frac{1}{2\sigma_\varepsilon^2}(l_{j2}\widehat{\beta}_j^2)\right],
\end{aligned}$$

where $l_{j2} = [\mathbf{x}_j'\mathbf{x}_j + \sigma_\varepsilon^2/(\gamma_2\sigma_\beta^2)]$ is the left hand side of the MME for β_j given $\delta_j = c$, $\widehat{\beta}_j = \mathbf{x}_j'\mathbf{w}/l_{j2}$, and $\mathbf{x}_j'\mathbf{w}$ is the right hand side of the MMEs. This form only depends on the scalar right hand side of the MMEs, which can be updated efficiently, and fixed constants for each iteration.

In the above example we observed a cancelling of the computationally difficult component $\mathbf{w}'\mathbf{w}$, which will happen for all forms of the ratio $\frac{f(\mathbf{w}|\delta_j=c,\boldsymbol{\theta},\mathbf{y})}{f(\mathbf{w}|\delta_j=\sim c,\boldsymbol{\theta}),\mathbf{y})}$. To calculate the probability updates for δ_j we will attempt to observe the form using $C = 2$ and extrapolate to an arbitrary C value. Therefore,

$$\mathbb{P}(\delta_j = 1|\boldsymbol{\theta}, \mathbf{y}) = \frac{f_j(\mathbf{w}|\delta_j = 1, \boldsymbol{\theta}, \mathbf{y})\pi_1}{f_j(\mathbf{w}|\delta_j = 1, \boldsymbol{\theta}, \mathbf{y})\pi_1 + f_j(\mathbf{w}|\delta_j = 2, \boldsymbol{\theta}, \mathbf{y})\pi_2}.$$

Let $\sigma_c^2 = \gamma_c\sigma_\beta^2$

$$\mathbb{P}(\delta_j = 1|\boldsymbol{\theta}, \mathbf{y}) = \frac{\left(\frac{\sigma_1^2\mathbf{x}_j'\mathbf{x}_j+\sigma_\varepsilon^2}{\sigma_\varepsilon^2}\right)^{-1/2}(2\pi\sigma_\varepsilon^2)^{-1/2}\exp\left[-\frac{1}{2\sigma_\varepsilon^2}\left(\mathbf{w}'\mathbf{w} - \frac{(\mathbf{x}_j'\mathbf{w})^2}{(\mathbf{x}_j'\mathbf{x}_j+\sigma_\varepsilon^2/\sigma_1^2)}\right)\right]\pi_1}{\left(\frac{\sigma_1^2\mathbf{x}_j'\mathbf{x}_j+\sigma_\varepsilon^2}{\sigma_\varepsilon^2}\right)^{-1/2}(2\pi\sigma_\varepsilon^2)^{-1/2}\exp\left[-\frac{1}{2\sigma_\varepsilon^2}\left(\mathbf{w}'\mathbf{w} - \frac{(\mathbf{x}_j'\mathbf{w})^2}{(\mathbf{x}_j'\mathbf{x}_j+\sigma_\varepsilon^2/\sigma_1^2)}\right)\right]\pi_1 + \left(\frac{\sigma_2^2\mathbf{x}_j'\mathbf{x}_j+\sigma_\varepsilon^2)}{\sigma_\varepsilon^2}\right)^{-1/2}(2\pi\sigma_\varepsilon^2)^{-1/2}\exp\left[-\frac{1}{2\sigma_\varepsilon^2}\left(\mathbf{w}'\mathbf{w} - \frac{(\mathbf{x}_j'\mathbf{w})^2}{(\mathbf{x}_j'\mathbf{x}_j+\sigma_\varepsilon^2/\sigma_2^2)}\right)\right]\pi_2}.$$

The component $(2\pi\sigma_e^2)^{-1/2}\exp\left[-\frac{1}{2\sigma_e^2}(\mathbf{w}'\mathbf{w})\right]$ is common to all the distributions and thus they can be partitioned out in the likelihood calculations. We therefore have

$$\mathbb{P}(\delta_j = 1|\boldsymbol{\theta}, \mathbf{y}) = \frac{(\frac{\sigma_1^2(\mathbf{x}_j'\mathbf{x}_j)+\sigma_\varepsilon^2)}{\sigma_\varepsilon^2})^{-1/2}\exp\left[\frac{\sigma_1^2}{2\sigma_\varepsilon^2}\left(\frac{(\mathbf{x}_j'\mathbf{w})^2}{(\sigma_1^2\mathbf{x}_j'\mathbf{x}_j+\sigma_\varepsilon^2)}\right)\right]\pi_1}{(\frac{\sigma_1^2(\mathbf{x}_j'\mathbf{x}_j)+\sigma_\varepsilon^2)}{\sigma_\varepsilon^2})^{-1/2}\exp\left[\frac{\sigma_1^2}{2\sigma_\varepsilon^2}\left(\frac{(\mathbf{x}_j'\mathbf{w})^2}{(\sigma_1^2\mathbf{x}_j'\mathbf{x}_j+\sigma_\varepsilon^2)}\right)\right]\pi_1 + (\frac{\sigma_2^2(\mathbf{x}_j'\mathbf{x}_j)+\sigma_\varepsilon^2)}{\sigma_\varepsilon^2})^{-1/2}\exp\left[\frac{\sigma_2^2}{2\sigma_\varepsilon^2}\left(\frac{(\mathbf{x}_j'\mathbf{w})^2}{(\sigma_2^2\mathbf{x}_j'\mathbf{x}_j+\sigma_\varepsilon^2)}\right)\right]\pi_2}.$$

This form is computationally important as it cancels the $\mathbf{w}'\mathbf{w}$ component so that we do no have to calculate it in each iteration. In order to calculate the posterior probability updates for an arbitrary number of components all that is required is $r_j = \mathbf{x}_j'\mathbf{w}$ and $\sigma_c^2 l_{jc} = \sigma_c^2(\mathbf{x}_j'\mathbf{x}_j) + \sigma_\varepsilon^2$. Then with respect to taking ratios of the components of interest it is sufficient to write

$$\log(\mathcal{L}_c) = \log[f(\mathbf{w}|\delta_j = c, \boldsymbol{\theta})] = -\frac{1}{2}\left[\log\left(\frac{\sigma_c^2 l_{jc}}{\sigma_\varepsilon^2}\right) - \frac{r_j^2}{\sigma_\varepsilon^2 l_{jc}}\right] + \log(\pi_c) \qquad (5)$$

and

$$\mathbb{P}(\delta_j = c|\boldsymbol{\theta}, \mathbf{y}) = \frac{\exp[\log(\mathcal{L}_c)]}{\sum_{c=1}^{C} \exp[\log(\mathcal{L}_c)]},$$

where C is the total number of mixture components. With the mixing proportions π_c being sampled in the previous iteration from the Dirichlet distribution. We use the numerically more stable version of the updates from Erbe *et al.*[16]

$$\mathbb{P}(\delta_j = c|\boldsymbol{\theta}, \mathbf{y}) = \frac{1}{\sum_{l=1}^{C} \exp[\log(\mathcal{L}_l) - \log(\mathcal{L}_c)]},$$

which can be shown to be equivalent by

$$\begin{aligned}
\mathbb{P}(\delta_j = c|\boldsymbol{\theta}, \mathbf{y}) &= \frac{\exp[\log(\mathcal{L}_c)]}{\exp[\log(\mathcal{L}_1)] + \exp[\log(\mathcal{L}_2)] + \ldots + \exp[\log(\mathcal{L}_C)]} \\
&= \frac{1}{\frac{\exp[\log(\mathcal{L}_1)]}{\exp[\log(\mathcal{L}_c)} + \frac{\exp[\log(\mathcal{L}_2)]}{\exp[\log(\mathcal{L}_c)]} + \ldots + \frac{\exp[\log(\mathcal{L}_C)]}{\exp[\log(\mathcal{L}_c)]}} \\
&= \frac{1}{\sum_{l=1}^{C} \exp[\log(\mathcal{L}_l) - \log(\mathcal{L}_c)]}. \qquad (6)
\end{aligned}$$

Combining Supplementary Equation (6) with the simple calculation of $\log(\mathcal{L}_c)$ using Supplementary Equation (5) we have all that is required to calculate the probabilities for the categorical distribution for an arbitrary number of mixture components.

Given these probabilities we need to sample from the categorical distribution, which determines which class the variant will be sampled from. With this sampled we can sample the effect from the relevant normal distribution or give it a zero effect. To sample from the categorical distribution we

- Create a vector of cumulative probabilities calculated from above $\mathbb{P}(\delta_j = 1|\boldsymbol{\theta}, \mathbf{y}), \mathbb{P}(\delta_j = 2|\boldsymbol{\theta}, \mathbf{y}), \ldots, \mathbb{P}(\delta_j = C|\boldsymbol{\theta}, \mathbf{y})$ ordered by category
- Accept the lowest c such that the cumulative probability $\geqslant u$, where u is sample form a $U(0, 1)$ distribution. For example if the sampled uniform value is 0.8 and the second group has a cumulative probability of 0.81 then we set $\delta_j = 2$.

Once we have sampled the distribution membership then we can sample the effect from

$$\beta_j|\delta_j = c, \boldsymbol{\theta}, \mathbf{y} \sim N(\mathbf{x}_j'\mathbf{w}/l_{jc}, \sigma_\varepsilon^2/l_{jc}), \tag{7}$$

where $l_{jc} = (\mathbf{x}_j'\mathbf{x}_j + \frac{\sigma_\varepsilon^2}{\sigma_c^2})$.

All that is required to do the sampling is the knowledge of $\mathbf{x}_j'\mathbf{x}_j$, which can be reconstructed from the LD matrix or estimated from the data, and $\mathbf{x}_j'\mathbf{w}$, which is the jth element of the right hand side, which can be updated efficiently using residual updating and reconstructed from summary statistics. This will highlighted in the next section.

Algorithm 1 – Individual level data algorithm

Initialise parameters and read genotypes and phenotypes in PLINK binary format
Initialise $\mathbf{y}^* = \mathbf{y} - \mathbf{X}\boldsymbol{\beta}$
for i :=1 **to** number of iterations **do**
  for j:=1 **to** p **do**
    Calculate $r_j^* = \mathbf{x}_j'\mathbf{y}^*$
    Calculate $r_j = r_j^* + \mathbf{x}_j'\mathbf{x}_j\beta_j^{(i-1)}$
    Calculate $\sigma_c^2 = \sigma_\beta^2\gamma_{\delta_j=c}$ for each of C classes (e.g., BayesR C=4 and $\boldsymbol{\gamma} = (0, 0.0001, 0.001, 0.01)$)
    Calculate the left hand side $l_{jc} = \mathbf{x}_j'\mathbf{x}_j + \frac{\sigma_\varepsilon^2}{\sigma_c^2}$ for each of the C classes
    Calculate the log densities of given $\delta_j = c$ using $\log(\mathcal{L}_c) = -\frac{1}{2}\left[\log\left(\frac{\sigma_c^2 l_{jc}}{\sigma_\varepsilon^2}\right) - \frac{r_j^2}{\sigma_\varepsilon^2 l_{jc}}\right] + \log(\pi_c)$, where π_c is the current
    Calculate the full conditional posterior probability for $\delta_j = c$ for C classes with $\mathbb{P}(\delta_j = c|\boldsymbol{\theta}, \mathbf{y}) = \frac{1}{\sum_{l=1}^{C}\exp[\log(\mathcal{L}_l) - \log(\mathcal{L}_c)]}$
    Using full conditional posterior probabilities sample class membership for $\beta_j^{(i)}$ using categorical random variable sampler
    Given class sample SNP effect $\beta_j^{(i)}$ from $N\left(\frac{r_j}{l_{jc}}, \frac{\sigma_\varepsilon^2}{l_{jc}}\right)$
    Given SNP effect adjust corrected phenotype side $(\mathbf{y}^*)^{(i)} = (\mathbf{y}^*)^{(i-1)} - \mathbf{x}_j\left(\beta_j^{(i)} - \beta_j^{(i-1)}\right)$
  od
  Sample update from full conditional for σ_β^2 from scaled inverse chi-squared distribution $\widetilde{\nu}_\beta = \nu_\beta + q$ and $\widetilde{S}^2{}_\beta = \frac{\nu_\beta S_\beta^2 + \sum_{j=1}^{q}\frac{\beta_j^2}{\gamma_c}}{\nu_\beta + q}$, where q is the number of non-zero variants
  Sample update from full conditional for σ_ε^2 from scaled inverse chi-squared distribution $\widetilde{\nu}_\varepsilon = n + \nu_\varepsilon$ and scale parameter $\widetilde{S}_\varepsilon^2 = \frac{SSE + \nu_\varepsilon S_\varepsilon^2}{n + \nu_\varepsilon}$ and $SSE = \mathbf{y}^{*\prime}\mathbf{y}^*$
  Sample update from full conditional for $\boldsymbol{\pi}$, which is Dirichlet($C, \mathbf{c} + \boldsymbol{\alpha}$), where $\mathbf{c}$ is a vector of length C and contains the counts of the number of variants in each variance class and $\boldsymbol{\alpha} = (1, \ldots, 1)$
  Estimate genetic variance for h_{SNP}^2 calculation using $\widehat{\sigma}_g^2 = V(\mathbf{X}\boldsymbol{\beta})$, where $V(\mathbf{X}\boldsymbol{\beta})$ is the sample variance of $\mathbf{X}\boldsymbol{\beta}$
  Calculate $h_{SNP}^2 = \frac{\widehat{\sigma}_g^2}{\widehat{\sigma}_g^2 + \widehat{\sigma}_\varepsilon^2}$
od

3. Supplementary Note 3 - Summary statistics based Bayesian multiple regression

We relate the phenotype to the set of genetic variants under the multiple linear regression model stated in Supplementary Equation (1). We can relate the multiple regression model to the the regression coefficients estimated from p simple linear regressions $\mathbf{b}$ from GWAS, by multiplying Supplementary Equation (1) by $\mathbf{D}^{-1}\mathbf{X}'$ where $\mathbf{D} = \text{diag}(\mathbf{x}_1'\mathbf{x}_1, \ldots, \mathbf{x}_p'\mathbf{x}_p)$ (assuming column centred genotypes) to arrive at

$$\mathbf{D}^{-1}\mathbf{X}'\mathbf{y} = \mathbf{D}^{-1}\mathbf{X}'\mathbf{X}\boldsymbol{\beta} + \mathbf{D}^{-1}\mathbf{X}'\boldsymbol{\varepsilon}. \tag{8}$$

Noting that the correlation matrix between all genetic markers $\mathbf{B} = \mathbf{D}^{-\frac{1}{2}}\mathbf{X}'\mathbf{X}\mathbf{D}^{-\frac{1}{2}}$ we rewrite the multiple regression model as

$$\mathbf{b} = \mathbf{D}^{-\frac{1}{2}}\mathbf{B}\mathbf{D}^{\frac{1}{2}}\boldsymbol{\beta} + \mathbf{D}^{-1}\mathbf{X}'\boldsymbol{\varepsilon}. \tag{9}$$

Assuming $\varepsilon_1, \ldots, \varepsilon_n$ are $N(0, \sigma_\varepsilon)$ the following likelihood can be proposed for the multiple regression coefficients $\boldsymbol{\beta}$

$$\mathcal{L}(\boldsymbol{\beta}; \mathbf{b}, \mathbf{D}, \mathbf{B}) := \mathcal{N}(\mathbf{b}; \mathbf{D}^{-\frac{1}{2}}\mathbf{B}\mathbf{D}^{\frac{1}{2}}\boldsymbol{\beta}, \mathbf{D}^{-\frac{1}{2}}\mathbf{B}\mathbf{D}^{-\frac{1}{2}}), \tag{10}$$

where $\mathcal{N}(\boldsymbol{\xi}; \boldsymbol{\mu}, \boldsymbol{\Sigma})$ represents the multivariate normal distribution with mean vector $\boldsymbol{\mu}$ and covariance matrix $\boldsymbol{\Sigma}$ for $\boldsymbol{\xi}$. If individual level data are available then inference about $\boldsymbol{\beta}$ can be obtained by replacing $\mathbf{D}$ and $\mathbf{B}$ with estimates $(\widehat{\mathbf{D}}, \widehat{\mathbf{B}})$ from the individual level data.

If individual level data are unavailable then we can replace $\mathbf{D}$ with $\widehat{\mathbf{D}} = \text{diag}[2n_1q_1(1 - q_1), \ldots, 2n_jq_j(1 - q_j)]$, where (n_j, q_j) are the sample size used to compute the simple linear regression coefficient and the variant allele frequency respectively. Furthermore, if we assume PLINK 1.9that genotype column j has been centred and scaled by $\sqrt{2q_j(1 - q_j)}$ then $\widehat{\mathbf{D}} = \text{diag}[n_1, \ldots, n_j]$. These approximations to $\mathbf{D}$, assume the the variant is in Hardy-Weinberg equilibrium, which may not be the true for all variants. Furthermore,

summary statistics in the public domain often do not include allele frequencies or report allele frequencies from a reference population. The methodology is susceptible to these deviations from the desired summary statistics for q_j, which is the allele frequency for the variant used in the analysis. Motivated by this drawback and the implementation of Zhu and Stephens[1] we seek an approximation to $\mathbf{D}$ that does not depend on q_j. For an individual variant from GWAS, the expression for the squared standard error of the estimated effect can be rearranged to arrive at

$$\mathbf{x}_j'\mathbf{x}_j = \frac{(\mathbf{y}'\mathbf{y})_j}{\widehat{\sigma}^2(\mathrm{b}_j)n_j + \mathrm{b}_j^2}. \tag{11}$$

Multiplying top and bottom of the right-hand side by $1/n$

$$\mathbf{x}_j'\mathbf{x}_j = \frac{(\mathbf{y}'\mathbf{y})_j/n_j}{\widehat{\sigma}^2(\mathrm{b}_j) + \mathrm{b}_j^2/n_j}, \tag{12}$$

and we note the numerator is the sample variance of the phenotype assuming it has been centred or a mean term fitting in the marginal regression. It is often the case that GWAS are performed on phenotypes that have been standardised to unit variance, which leads to

$$\mathbf{x}_j'\mathbf{x}_j = \frac{1}{\widehat{\sigma}^2(\mathrm{b}_j) + \mathrm{b}_j^2/n_j}. \tag{13}$$

If this is not the case we note that $\mathbf{y}'\mathbf{y}$ is a constant that can be shown to not contribute to the updates of any parameter in the sampling routine, which was also observed and proven in Mak *et al.*[22]. However, initialisation of the hyperparameters for the sampling of the marker effect variance, σ_β^2, of the SBayesR model requires an estimate of the phenotypic variance (more detail below) to improve mixing. Initially, we reconstruct $\mathbf{x}_j'\mathbf{x}_j$ using the reported allele frequency and sample size summary statistics for each variant. An estimate of the phenotypic variance $(\mathbf{y}'\mathbf{y})/n$ is then reconstructed by calculating the total sum of

squares for each variant

$$(\mathbf{y}'\mathbf{y})_j = \widehat{\sigma}^2(\mathrm{b}_j)\mathbf{x}_j'\mathbf{x}_j(n-2) + \mathrm{b}_j^2\mathbf{x}_j'\mathbf{x}_j.$$

and taking the ratio of the median over the set of $(\mathbf{y}'\mathbf{y})_j$ and n_j values, which Yang *et al.*[23] suggest is reliable. Given the estimated phenotypic variance we reconstruct $\mathbf{D}$ using the more reliable Supplementary Equation (12).

Similarly, we replace $\mathbf{B}$, the LD correlation matrix between the genotypes at all markers in the population, which the genotypes in the sample are assumed to be a random sample, with $\widehat{\mathbf{B}}$ an estimate calculated from a population reference that is assumed to closely resemble the sample used to generate the GWAS summary statistics. Zhu and Stephens[1] discuss further the theoretical properties of a similar likelihood and approximate reconstruction.

3.1. Sampling β_j

As shown in the previous section the update of the jth SNP effect involves the calculation of $r_j = \mathbf{x}_j'\mathbf{w}$ and $l_{jc} = \sigma_c^2(\mathbf{x}_j'\mathbf{x}_j) + \sigma_\varepsilon^2$. We require

$$r_j = \mathbf{x'}_j\mathbf{w} = \mathbf{x'}_j[\mathbf{y} - \mathbf{X}_{-j}\boldsymbol{\beta}_{-j}]$$

for use in Supplementary Equation (5) and Supplementary Equation (7). To find this we define the corrected right hand side as $\mathbf{X}'\mathbf{y}$ corrected for all current $\boldsymbol{\beta}$

$$\mathbf{r}^* = \mathbf{X}'\mathbf{y} - \mathbf{X}'\mathbf{X}\boldsymbol{\beta} = \mathbf{X}'\mathbf{y} - \mathbf{X}'\mathbf{X}_{-\mathbf{j}}\boldsymbol{\beta}_{-j} - \mathbf{X}'\mathbf{x}_j\beta_j,$$

where $\mathbf{r}^*$ is a vector of dimension $p \times 1$ and $\mathbf{X}_{-j}$ is $\mathbf{X}$ minus the jth column. We reconstruct $\mathbf{X}'\mathbf{y}$ using the GWAS effect estimates $\mathbf{b}$ and $\mathbf{D}$ such that $\mathbf{X}'\mathbf{y} = \mathbf{Db}$. The jth element of $\mathbf{r}^*$ is

$$r_j^* = \mathbf{x}_j'\mathbf{y} - \mathbf{x}_j'\mathbf{X}_{-j}\boldsymbol{\beta}_{-j} - \mathbf{x'}_j\mathbf{x}_j\beta_j.$$

$$r_j^* + \mathbf{x'}_j\mathbf{x}_j\beta_j = \mathbf{x}_j'\mathbf{y} - \mathbf{x}_j'\mathbf{X}_{-j}\boldsymbol{\beta}_{-j} = \mathbf{x}_j'(\mathbf{y} - \mathbf{X}_{-j}\boldsymbol{\beta}_{-j}) = r_j.$$

Therefore, for each SNP we calculate

$$r_j = \mathbf{x'}_j \mathbf{w} = r_j^* + \mathbf{x'}_j \mathbf{x}_j \beta_j.$$

This can be used in conjunction with Supplementary Equation (5) to calculate the class membership probabilities and then update the SNP effect using Supplementary Equation (7), which only requires r_j and the diagonal elements of $\mathbf{X'X}$. The matrix $\mathbf{X'X}$ is easily calculated from summary statistics via $\mathbf{X'X} = \mathbf{D}^{\frac{1}{2}}\mathbf{B}\mathbf{D}^{\frac{1}{2}}$. Given a new β_j in MCMC iteration m we can update the corrected right hand side ($\mathbf{r}^*$) by

$$(\mathbf{r}^*)^{(m+1)} = (\mathbf{r}^*)^{(m)} - \mathbf{X'x}_j[\beta_j^{(m+1)} - \beta_j^{(m)}].$$

This can be shown by noting

$$\begin{aligned}
(\mathbf{r}^*) &= \mathbf{X'y} - \mathbf{X'X}\boldsymbol{\beta} = \mathbf{X'y} - \mathbf{X'X_{-j}}\boldsymbol{\beta}_{-j} - \mathbf{X'x}_j\beta_j \\
(\mathbf{r}^*)^{(m)} &= \mathbf{X'y} - \mathbf{X'X_{-j}}\boldsymbol{\beta}_{-j} - \mathbf{X'x}_j\beta_j^{(m)} \\
(\mathbf{r}^*)^{(m+1)} - (\mathbf{r}^*)^{(m)} &= \mathbf{X'y} - \mathbf{X'X_{-j}}\boldsymbol{\beta}_{-j} - \mathbf{X'x}_j\beta_j^{(m+1)} - \mathbf{X'y} + \mathbf{X'X_{-j}}\boldsymbol{\beta}_{-j} + \mathbf{X'x}_j\beta_j^{(m)} \\
(\mathbf{r}^*)^{(m+1)} &= (\mathbf{r}^*)^{(m)} - \mathbf{X'x}_j(\beta_j^{(m+1)} - \beta_j^{(m)}).
\end{aligned}$$

This forms the basis of the RHS updating scheme. The beauty of this updating scheme is that it only requires scalar operations and one vector subtraction. If the effect is 0 for the jth SNP no sampling of the effect is required.

The LD matrix only enters into the sampling routine through the diagonal elements in the calculation of l_{jc}, which are scalar and efficiently stored, and in the $\mathbf{X'x}_j$ of the residual update. If we are only updating elements of the $\mathbf{r}^*$ that are in LD with the current SNP then the update is even more efficient as we only have to do the vector subtraction on the non-zero elements. The LD approximation using a sparse matrix or formed by block diagonalising the matrix using a window based approach leads to such a scenario. The efficient updating scheme and the fact that we don't have to store and read the genotype

matrix makes this method very efficient. We also avoid the dot product computation of $\mathbf{x}'\mathbf{y}^*$, which was present in the previous BayesR algorithm (Algorithm 1).

3.2. Sampling σ_ε^2

The updating of the σ_ε^2 is one of the most critical parts of the summary statistics Gibbs sampling algorithm because its update requires the reconstruction of unobservables, which if we had the full LD matrix could be approximated very well. However, when a sparse LD matrix is used the updating of σ_ε^2 can become very unstable due to the approximation.

We begin by deriving the full conditional distribution for σ_ε^2. The parameter σ_ε^2 appears only in its prior

$$f(\sigma_\varepsilon^2; \nu_\varepsilon, S_\varepsilon^2) = \frac{(S_\varepsilon^2 \nu_\varepsilon/2)^{\nu/2}}{\Gamma(\nu_\varepsilon/2)} \frac{\exp(-\nu_\varepsilon S_\varepsilon^2/2\sigma_\varepsilon^2)}{(\sigma_\varepsilon^2)^{1+\nu_\varepsilon/2}},$$

and in the conditional distribution of $\mathbf{y}$ given all the unknowns,

$$f(\mathbf{y}|\boldsymbol{\theta}) = (2\pi\sigma_\varepsilon^2)^{-n/2} \exp\left[-\frac{(\mathbf{y}-\mathbf{X}\boldsymbol{\beta})'(\mathbf{y}-\mathbf{X}\boldsymbol{\beta})}{2\sigma_\varepsilon^2}\right].$$

Therefore,

$$\begin{aligned} f(\sigma_\varepsilon^2|\boldsymbol{\theta}_{-\sigma_\varepsilon^2}, \mathbf{y}) &= \frac{(S_\varepsilon^2 \nu_\varepsilon/2)^{\nu/2}}{\Gamma(\nu_\varepsilon/2)} \frac{\exp(-\nu_\varepsilon S_\varepsilon^2/2\sigma_\varepsilon^2)}{(\sigma_\varepsilon^2)^{1+\nu_\varepsilon/2}} (2\pi\sigma_\varepsilon^2)^{-n/2} \exp\left[-\frac{(\mathbf{y}-\mathbf{X}\boldsymbol{\beta})'(\mathbf{y}-\mathbf{X}\boldsymbol{\beta})}{2\sigma_\varepsilon^2}\right] \\ &\propto \frac{\exp(-\nu_\varepsilon S_\varepsilon^2/2\sigma_\varepsilon^2)}{(\sigma_\varepsilon^2)^{1+\nu_\varepsilon/2}} (\sigma_\varepsilon^2)^{-n/2} \exp\left[-\frac{SSE}{2\sigma_\varepsilon^2}\right] \\ &\propto (\sigma_\varepsilon^2)^{-(n+2+\nu_\varepsilon)/2} \exp\left[-\frac{SSE + \nu_\varepsilon S_\varepsilon^2}{2\sigma_\varepsilon^2}\right], \end{aligned}$$

where SSE is the sum of squared errors of prediction. This can be recognised as the kernel of a scaled inverse chi-square distribution with degrees of freedom $\widetilde{\nu}_\varepsilon = n + \nu_\varepsilon$ and scale parameter $\widetilde{S}_\varepsilon^2 = \frac{SSE+\nu_\varepsilon S_\varepsilon^2}{n+\nu_\varepsilon}$.

If we don't have the individual level data then we cannot observe certain components of the SSE. Assuming that the fixed effects have already been corrected from the phenotype

then the SSE can be written as

$$\begin{aligned} SSE &= \mathbf{y}'\mathbf{y} - 2(\mathbf{X}\boldsymbol{\beta})'\mathbf{y} + (\mathbf{X}\boldsymbol{\beta})'\mathbf{X}\boldsymbol{\beta} \\ &= \mathbf{y}'\mathbf{y} - 2\boldsymbol{\beta}'\mathbf{D}\mathbf{b} + \boldsymbol{\beta}'\mathbf{X}'\mathbf{X}\boldsymbol{\beta}, \end{aligned} \tag{14}$$

where $\mathbf{D} = \text{diag}(\mathbf{X}'\mathbf{X})$ and $\mathbf{b} = \mathbf{D}^{-1}\mathbf{X}'\mathbf{y}$. We don't observe $\mathbf{y}'\mathbf{y}$ but can we approximate it from the GWAS results. For an individual variant from GWAS, the expression for the squared standard error of the estimated effect can be rearranged to arrive at

$$(\mathbf{y}'\mathbf{y})_j = \widehat{\sigma}^2(\mathrm{b}_j)\mathbf{x}_j'\mathbf{x}_j(n-2) + \mathrm{b}_j^2\mathbf{x}_j'\mathbf{x}_j. \tag{15}$$

Yang *et al.*[23] suggest that the mean across all SNPs is a good estimate of $\mathbf{y}'\mathbf{y}$.

The estimation of SSE highlights the problems with approximating the individual data algorithm using summary data and a sparse $\mathbf{X}'\mathbf{X}$. For example, the SSE equation contains the genetic effect estimates $\mathbf{b}$, which were calculated using the full $\mathbf{X}$ matrix, whereas the $\mathbf{X}'\mathbf{X}$ is now banded. In the individual data algorithm each marker requires the sampled value of σ_ε^2 in each calculation of l_{jc} and the sample of the SNP effect using Supplementary Equation (7). If the full LD matrix is used in the algorithm routine then SSE is the same for each variant. However, as each variant ignores a unique set of LD correlations we propose a marker specific variance $(\sigma_\varepsilon^2)_j$, which attempts to correct for the discrepancy between the fact that a sparse $\mathbf{X}'\mathbf{X}$ has replaced the full $\mathbf{X}'\mathbf{X}$ in Supplementary Equation (14).

We can attempt to improve on this by estimating a marker specific residual variance that only contains a contribution from non-zero LD correlation matrix elements of $\boldsymbol{\beta}'\mathbf{X}'\mathbf{X}\boldsymbol{\beta}$, which is specific to each variant.

$$(\widehat{\sigma}_\varepsilon^2)_j = \frac{SSE_j}{n_j - 1}. \tag{16}$$

The computation of $\boldsymbol{\beta}'\mathbf{X}'\mathbf{X}\boldsymbol{\beta}$ for each variant is expensive. Therefore, we use the corrected right hand side to efficiently compute the marker specific residual variance. Generally, corrected right hand side $\mathbf{r}^* = \mathbf{X}'\mathbf{y} - \mathbf{X}'\mathbf{X}\boldsymbol{\beta}$ can be combined with Supplementary Equation (14) for a more efficient update

$$\begin{aligned} SSE &= \mathbf{y}'\mathbf{y} - 2\boldsymbol{\beta}'\mathbf{X}'\mathbf{y} + \boldsymbol{\beta}'\mathbf{X}'\mathbf{X}\boldsymbol{\beta} \\ &= \mathbf{y}'\mathbf{y} - \boldsymbol{\beta}'\mathbf{X}'\mathbf{y} - \boldsymbol{\beta}'\mathbf{X}'\mathbf{y} + \boldsymbol{\beta}'\mathbf{X}'\mathbf{X}\boldsymbol{\beta} \\ &= \mathbf{y}'\mathbf{y} - \boldsymbol{\beta}'\mathbf{X}'\mathbf{y} - \boldsymbol{\beta}'\mathbf{r}^* \qquad (17) \\ &= \mathbf{y}'\mathbf{y} - \boldsymbol{\beta}'(\mathbf{r}^* + \mathbf{X}'\mathbf{y}) = \mathbf{y}'\mathbf{y} - \sum_{k=1}^{p:\mathrm{LD}\neq 0} \beta_k'[r_k^* + (\mathbf{X}'\mathbf{y})_k] - \sum_{k=1}^{p:\mathrm{LD}=0} \beta_k'[r_k^* + (\mathbf{X}'\mathbf{y})_k]. \qquad (18) \end{aligned}$$

This allows for an efficient calculation of the per variant marker effects variance

$$SSE_j = (\mathbf{y}'\mathbf{y})_j - \sum_{k=1}^{p:\mathrm{LD}\neq 0} \beta_k'[{r_k}^* + (\mathbf{X}'\mathbf{y})_k],$$

which intuitively is the individual variant total sum of squares subtract the contributions from those other variants in LD with variant j. The individual variance is calculated using Supplementary Equation (16) and used in the per variant calculation of l_{jc} and the sampling of the effect from Supplementary Equation (7). These values are updated every 100 iterations of the MCMC chain as a compromise between accuracy and computational efficiency. After the completion of the sampling of all marker effects the SSE is calculated using Supplementary Equation (17) and a global σ_ε^2 is sampled from a scaled inverse chi-square distribution with degrees of freedom $\widetilde{\nu}_\varepsilon = n + \nu_\varepsilon$ and scale parameter $\widetilde{S}_\varepsilon^2 = \frac{SSE + \nu_\varepsilon S_\varepsilon^2}{n + \nu_\varepsilon}$. This value is only used for h_{SNP}^2 estimation.

3.3. Computing estimate of genotypic variance

We estimate the genetic variance σ_g^2 by computing the sample variance of the vector $\mathbf{X}\boldsymbol{\beta}$ defined to be $V(X\boldsymbol{\beta})$ by treating $\boldsymbol{\beta}$ as fixed at the sampled value of $\boldsymbol{\beta}^{(i)}$ at the ith MCMC iteration and $\mathbf{X}$ as random as per the definition of genetic variance (similar to Zhu and Stephens[1]). Conditioning on the sampled value of $\boldsymbol{\beta}$ in each MCMC iteration, $V(X\boldsymbol{\beta})$ can

be approximated by MSS/n, where

$$MSS = \boldsymbol{\beta}'\mathbf{X}'\mathbf{X}\boldsymbol{\beta}.$$

Again to efficiently update this we use $\mathbf{r}^* = \mathbf{X}'\mathbf{y} - \mathbf{X}'\mathbf{X}\boldsymbol{\beta} \rightarrow (\mathbf{X}'\mathbf{y} - \mathbf{r}^*) = \mathbf{X}'\mathbf{X}\boldsymbol{\beta}$ and thus

$$\begin{aligned} MSS &= \boldsymbol{\beta}'(\mathbf{X}'\mathbf{y} - \mathbf{r}^*) \\ &= \boldsymbol{\beta}'\mathbf{X}'\mathbf{y} - \boldsymbol{\beta}'r^*. \end{aligned}$$

Therefore, for each iteration of the MCMC chain we estimate σ_g^2 using the corrected right hand side, the current sampled $\boldsymbol{\beta}$, $\mathbf{X}'\mathbf{y}$ and compute

$$\widehat{\sigma}_g^2 = V(\mathbf{X}\boldsymbol{\beta}) = MSS/n.$$

4. Supplementary Note 4 - Method summary and implementation

The full joint distribution of the data $(\mathbf{b}, \mathbf{B}, \mathbf{D})$ and the model parameters $\boldsymbol{\theta} = (\boldsymbol{\beta}', \boldsymbol{\pi}', \sigma_\beta^2, \sigma_\varepsilon^2)'$ is

$$\begin{aligned} f(\boldsymbol{\theta}, \mathbf{b}, \mathbf{B}, \mathbf{D}) = &\, |2\pi\sigma_\varepsilon^2\mathbf{D}^{-1/2}\mathbf{B}\mathbf{D}^{-1/2}|^{-1/2}\times \\ &\exp\left[-\frac{(\mathbf{b} - \mathbf{D}^{-1/2}\mathbf{B}\mathbf{D}^{1/2}\boldsymbol{\beta})'(\mathbf{D}^{-1/2}\mathbf{B}\mathbf{D}^{-1/2})^{-1}(\mathbf{b} - \mathbf{D}^{-1/2}\mathbf{B}\mathbf{D}^{1/2}\boldsymbol{\beta})}{2\sigma_\varepsilon^2}\right]\times \\ &\prod_{j=1}^{p}\sum_{c=1}^{C}\pi_c(2\sigma_\beta^2\gamma_c)^{-1/2}\exp\left[-\frac{\beta_j^2}{2\sigma_\beta^2\gamma_c}\right]\times \\ &\prod_{c=1}^{C}\pi_c^{\alpha_c-1}\times \\ &\frac{\exp(-\nu_\beta S_\beta^2/2\sigma_\beta^2)}{(\sigma_\beta^2)^{1+\nu_\beta/2}}\times \\ &\frac{\exp(-\nu_\varepsilon S_\varepsilon^2/2\sigma_\varepsilon^2)}{(\sigma_\varepsilon^2)^{1+\nu_\varepsilon/2}}\,, \end{aligned}$$

where $\boldsymbol{\alpha} = (\alpha_1, \ldots, \alpha_c, \ldots, \alpha_C)$ and $\alpha_c = 1$ are Dirichlet prior hyperparameters, the variance

weights γ_c are pre-specified and are taken to be magnitudes of 10, for example for default $C = 4$ the vector of weights are $\gamma = (0, 0.01, 0.1, 1.0)'$, S^2_β and ν_β and S^2_ε and ν_ε are the scale parameter and degrees of freedom respectively for the prior of the scaled inverse chi-squared distribution prior of the variance components. We use a Gibbs sampling algorithm to draw posterior samples for all parameters will the following implementation.

The summary based Bayesian multiple regression method has been implemented in a software tool named Genome-wide Complex Trait Bayesian analyses (GCTB). The tool has been written in the C++ programming language and is available and freely distributable under a MIT License. The method requires the following data:

- The univariate regression effects from GWAS $\mathbf{b}$.
- The standard error estimates for each genetic effect from univariate regression $\widehat{\sigma}^2(\mathbf{b})$.
- An LD matrix calculated from the cohort or a population matched reference $\mathbf{B} = \mathbf{D}^{-1/2}\mathbf{X}'\mathbf{X}\mathbf{D}^{-1/2}$, where $\mathbf{X}$ is the genotype matrix from the cohort to analysed or a reference. If we assume that the SNP covariates have been mean adjusted, or the mean has been fitted in the univariate regression analysis, then $\mathbf{D}$ is a diagonal matrix with diagonal elements that should be very well approximated in large samples by Supplementary Equation (13). If the genotypes are assumed to have been centred and scaled then $\mathbf{D}$ is a diagonal matrix with diagonal elements n_j. The algorithm requires $\mathbf{X}'\mathbf{X}$ and thus using effects estimated from a PLINK GWAS we have $\mathbf{X}'\mathbf{X} = \mathbf{D}^{1/2}\mathbf{B}\mathbf{D}^{1/2}$.
- The algorithm also requires $\mathbf{X}'\mathbf{y}$, which from the least squares solutions can be recovered $\widehat{\mathbf{b}} = \text{diag}(\mathbf{X}'\mathbf{X})^{-1}\mathbf{X}'\mathbf{y} = \mathbf{D}^{-1}\mathbf{X}'\mathbf{y}$ and thus $\mathbf{X}'\mathbf{y} = \mathbf{D}\widehat{\mathbf{b}}$.

Parameters to be estimated

- The joint genetic effects $\boldsymbol{\beta}_0$. Initialised as all zeros.
- The proportion of effects in each class $\boldsymbol{\pi}$. This is by default the original BayesR model with four components and initialised such that a large proportion of variants have no effect, for example, $\boldsymbol{\pi}_0 = (0.95, 0.02, 0.02, 0.01)$.

- The vector γ specifies the weights for the mixture normal class variances, which are by default set to $(0, 0.01, 0.1, 1)$. These deviate from the BayesR model $(0, 0.0001, 0.001, 0.01)'$ as they represent the weights for the marker effect variance as opposed to the genetic variance as in Erbe *et al.*[16] and Moser *et al.*[6].
- The variance components include the marker effect variance σ_β^2 and the residual variance σ_ε^2. The initial value of the residual variance is set to $(\sigma_\varepsilon^2)_0 = \frac{SSE}{n-1}$, where $SSE = \mathbf{y}'\mathbf{y} - 2\boldsymbol{\beta}_0'\mathbf{X}'\mathbf{y} + \boldsymbol{\beta}_0'\mathbf{X}'\mathbf{X}\boldsymbol{\beta}_0 = \mathbf{y}'\mathbf{y}$ (given $\boldsymbol{\beta}_0 = \mathbf{0}$), and $\mathbf{y}'\mathbf{y} = \frac{1}{p}\sum_{j=1}^{p}(\mathbf{y}'\mathbf{y})_j$ where $(\mathbf{y}'\mathbf{y})_j = \widehat{\sigma}^2(\widehat{b}_j)\mathbf{x}_j'\mathbf{x}_j(n-2) + \widehat{b}_j^2\mathbf{x}_j'\mathbf{x}_j$, which is reconstructed from the summary statistics from the univariate regression for each variant. The parameter σ_β^2 is initialised as $(\sigma_\beta^2)_0 = (\sigma_g^2)_0/[(1-(\boldsymbol{\pi}_0)_1)\sum_j 2q_j(1-q_j)]$[24], where q_j is the allele frequency of allele j $(\sigma_g^2)_0$ is the genotypic variance and is set to $(\sigma_g^2)_0 = h_{SNP}^2\frac{\mathbf{y}'\mathbf{y}}{(n-1)}$ and $h_{SNP}^2 = 0.5$ is set by default set a starting h_{SNP}^2.

Hyperparameters to be set

- The degrees of freedom ν and scale parameters S^2 for the scale inverse chi-squared distribution, which form the priors for the σ_β^2 and σ_ε^2 parameters are required to be set. For both the degrees of freedom are set to 4[19] and $S_\alpha^2 = \frac{(\nu_\beta-2)(\sigma_\beta^2)_0}{\nu_\beta} = \frac{(\sigma_\beta^2)_0}{2}$ and $S_\varepsilon^2 = \frac{(\nu_\varepsilon-2)(\sigma_\varepsilon^2)_0}{\nu_\varepsilon} = \frac{(\sigma_\varepsilon^2)_0}{2}$, which comes from a method of moments estimator for the scale parameter.

Algorithm 2 Summary data algorithm

Initialise parameters and read summary statistics
Reconstruct $\mathbf{X}'\mathbf{X}$ and $\mathbf{X}'\mathbf{y}$ from summary statistics and LD reference panel
Calculate $\mathbf{r}^* = \mathbf{X}'\mathbf{y} - \mathbf{X}'\mathbf{X}\boldsymbol{\beta}$
for i :=1 **to** number of iterations **do**
  for j :=1 **to** p **do**
    Calculate $\mathbf{r}_j = \mathbf{r}_j^* + \mathbf{x}_j'\mathbf{x}_j\beta_j$
    Calculate $\sigma_c^2 = \sigma_\beta^2\gamma_{\delta_j=c}$ for each fo C classes (e.g., SBayesR C=4 and $\gamma = (0, 0.01, 0.1, 1)'$)
    Calculate the left hand side $l_{jc} = \mathbf{x}_j'\mathbf{x}_j + \frac{\sigma_\varepsilon^2}{\sigma_c^2}$ for each of the C classes
    Calculate the log densities of given $\delta_j = c$ using $\log(\mathcal{L}_c) = -\frac{1}{2}\left[\log\left(\frac{\sigma_c^2 l_{jc}}{\sigma_\varepsilon^2}\right) - \frac{r_j^2}{\sigma_\varepsilon^2 l_{jc}}\right] + \log(\pi_c)$, where π_c is the current
    Calculate the full conditional posterior probability for $\delta_j = c$ for C classes with $\mathbb{P}(\delta_j = c|\boldsymbol{\theta}, \mathbf{y}) = \frac{1}{\sum_{l=1}^{C}\exp[\log(\mathcal{L}_l) - \log(\mathcal{L}_c)]}$
    Using full conditional posterior probabilities sample class membership for $\beta_j^{(i)}$ using categorical random variable sampler
    Given class sample SNP effect $\beta_j^{(i)}$ from full conditional $N\left(\frac{\mathbf{r}_j}{l_{jc}}, \frac{\sigma_\varepsilon^2}{l_{jc}}\right)$
    Given SNP effect adjust corrected right hand side $(\mathbf{r}^*)^{(i+1)} = (\mathbf{r}^*)^{(i)} - \mathbf{X}'\mathbf{x}_j\left(\beta_j^{(i+1)} - \beta_j^{(i)}\right)$. $\mathbf{X}'\mathbf{x}_j$ is the jth column of $\mathbf{X}'\mathbf{X}$.
  od
  Sample update from full conditional for σ_β^2 from scaled inverse chi-squared distribution $\tilde{\nu}_\beta = \nu_0 + q$ and $\tilde{\tau^2}_\beta = \frac{\nu_0\tau_0^2 + \sum_{j=1}^{q}\frac{\beta_j^2}{\gamma_{\delta_j}}}{\nu_0 + q}$, where q is the number of non-zero variants
  Sample update from full conditional for σ_ε^2 from scaled inverse chi-squared distribution $\tilde{\nu}_\varepsilon = n + \nu_\varepsilon$ and scale parameter $\tilde{\tau}_\varepsilon^2 = \frac{SSE + \nu_\varepsilon\tau_\varepsilon^2}{n + \nu_\varepsilon}$ and $SSE = \mathbf{y}'\mathbf{y} - \boldsymbol{\beta}'\mathbf{r}^* - \boldsymbol{\beta}'\mathbf{X}'\mathbf{y}$
  Sample update from full conditional for $\boldsymbol{\pi}$, which is Dirichlet($C, \mathbf{c} + \boldsymbol{\beta}$), where $\mathbf{c}$ is a vector of length C and contains the counts of the number of variants in each variance class.
  Calculate genetic variance for h_{SNP}^2 calculation using $\widehat{\sigma}_g^2 = MSS/n$, where $MSS = \widehat{\boldsymbol{\beta}}'\mathbf{X}'\mathbf{y} - \widehat{\boldsymbol{\beta}}' r^*$
  Calculate $h_{SNP}^2 = \frac{\widehat{\sigma}_g^2}{\widehat{\sigma}_g^2 + \widehat{\sigma}_\varepsilon^2}$
od

5. Supplementary Note 5 - Full-data likelihood equivalence

If we have access to the individual level data then under the multiple regression model the full-data likelihood for inferring $\boldsymbol{\beta}$ is

$$\mathcal{L}(\boldsymbol{\beta}; \mathbf{y}, \mathbf{X}, \sigma_\varepsilon^2) = (2\pi\sigma_\varepsilon^2)^{-n/2}\exp\left[-\frac{(\mathbf{y} - \mathbf{X}\boldsymbol{\beta})'(\mathbf{y} - \mathbf{X}\boldsymbol{\beta})}{2\sigma_\varepsilon^2}\right]. \quad (19)$$

Zhu and Stephens[1] show that under their likelihood and the assumptions that the LD correlation matrix $\widehat{\mathbf{B}}$ has been computed from the genotypes $\mathbf{X}$, $n > p$ and that $\sigma_\varepsilon^2 = n^{-1}\mathbf{y}'\mathbf{y}$ that the full-data likelihood is equivalent to their likelihood up to a constant that does not depend on $\boldsymbol{\beta}$. We will seek to arrive at the same conclusion under the likelihood proposed in Supplementary Equation (10). Replacing in Supplementary Equation (10) $\mathbf{B}$ with $\widehat{\mathbf{B}}$ and

$\mathbf{D}$ with $\widehat{\mathbf{D}}$ then the summary data likelihood is

$$\mathcal{L}(\boldsymbol{\beta}; \mathbf{b}, \widehat{\mathbf{B}}, \widehat{\mathbf{D}}) = |2\pi\sigma_\varepsilon^2 \widehat{\mathbf{D}}^{-1/2}\widehat{\mathbf{B}}\widehat{\mathbf{D}}^{-1/2}|^{-1/2} \times \\ \exp\left[-\frac{(\mathbf{b} - \widehat{\mathbf{D}}^{-1/2}\widehat{\mathbf{B}}\widehat{\mathbf{D}}^{1/2}\boldsymbol{\beta})'(\widehat{\mathbf{D}}^{-1/2}\widehat{\mathbf{B}}\widehat{\mathbf{D}}^{-1/2})^{-1}(\mathbf{b} - \widehat{\mathbf{D}}^{-1/2}\widehat{\mathbf{B}}\widehat{\mathbf{D}}^{1/2}\boldsymbol{\beta})}{2\sigma_\varepsilon^2} \right].$$

When $n > p$ then $\widehat{\mathbf{B}}$ computed from the sample genotypes is non-singular and thus we require this assumption in the statement of the likelihood. Taking the logarithm and expanding we have

$$\log[\mathcal{L}(\boldsymbol{\beta}; \mathbf{b}, \widehat{\mathbf{B}}, \widehat{\mathbf{D}})] = -\frac{p}{2}\log(2\pi\sigma_\varepsilon^2) - \frac{1}{2}\log|\widehat{\mathbf{D}}^{-1/2}\widehat{\mathbf{B}}\widehat{\mathbf{D}}^{-1/2}| \; - \frac{1}{2\sigma_\varepsilon^2}\mathbf{b}'(\widehat{\mathbf{D}}^{-1/2}\widehat{\mathbf{B}}\widehat{\mathbf{D}}^{-1/2})^{-1}\mathbf{b} \; + \\ \frac{1}{\sigma_\varepsilon^2}(\widehat{\mathbf{D}}^{-1/2}\widehat{\mathbf{B}}\widehat{\mathbf{D}}^{1/2}\boldsymbol{\beta})'(\widehat{\mathbf{D}}^{-1/2}\widehat{\mathbf{B}}\widehat{\mathbf{D}}^{-1/2})^{-1}\mathbf{b} \; - \\ \frac{1}{2\sigma_\varepsilon^2}(\widehat{\mathbf{D}}^{-1/2}\widehat{\mathbf{B}}\widehat{\mathbf{D}}^{1/2}\boldsymbol{\beta})'(\widehat{\mathbf{D}}^{-1/2}\widehat{\mathbf{B}}\widehat{\mathbf{D}}^{-1/2})^{-1}(\widehat{\mathbf{D}}^{-1/2}\widehat{\mathbf{B}}\widehat{\mathbf{D}}^{1/2}\boldsymbol{\beta}).$$

Similarly for the full data likelihood, we take the logarithm and expand

$$\log[\mathcal{L}(\boldsymbol{\beta}; \mathbf{y}, \mathbf{X}, \sigma_\varepsilon^2)] = -\frac{n}{2}\log(2\pi\sigma_\varepsilon^2) - \frac{1}{2\sigma_\varepsilon^2}\mathbf{y}'\mathbf{y} + \frac{1}{\sigma_\varepsilon^2}\boldsymbol{\beta}'\mathbf{X}'\mathbf{y} - \frac{1}{2\sigma_\varepsilon^2}\boldsymbol{\beta}'\mathbf{X}'\mathbf{X}\boldsymbol{\beta}. \tag{20}$$

Looking at the difference we have

$$\log[\mathcal{L}(\boldsymbol{\beta}; \mathbf{y}, \mathbf{X}, \sigma_\varepsilon^2)] - \log[\mathcal{L}(\boldsymbol{\beta}; \mathbf{b}, \widehat{\mathbf{B}}, \widehat{\mathbf{D}})] = -\frac{n}{2}\log(2\pi\sigma_\varepsilon^2) - \frac{1}{2\sigma_\varepsilon^2}\mathbf{y}'\mathbf{y} + \frac{1}{\sigma_\varepsilon^2}\boldsymbol{\beta}'\mathbf{X}'\mathbf{y} - \frac{1}{2\sigma_\varepsilon^2}\boldsymbol{\beta}'\mathbf{X}'\mathbf{X}\boldsymbol{\beta} - \\ \frac{p}{2}\log(2\pi\sigma_\varepsilon^2) + \frac{1}{2}\log|\widehat{\mathbf{D}}^{-1/2}\widehat{\mathbf{B}}\widehat{\mathbf{D}}^{-1/2}| \; + \frac{1}{2\sigma_\varepsilon^2}\mathbf{b}'(\widehat{\mathbf{D}}^{-1/2}\widehat{\mathbf{B}}\widehat{\mathbf{D}}^{-1/2})^{-1}\mathbf{b} \; - \\ \frac{1}{\sigma_\varepsilon^2}(\widehat{\mathbf{D}}^{-1/2}\widehat{\mathbf{B}}\widehat{\mathbf{D}}^{1/2}\boldsymbol{\beta})'(\widehat{\mathbf{D}}^{-1/2}\widehat{\mathbf{B}}\widehat{\mathbf{D}}^{-1/2})^{-1}\mathbf{b} \; + \frac{1}{2\sigma_\varepsilon^2}(\widehat{\mathbf{D}}^{-1/2}\widehat{\mathbf{B}}\widehat{\mathbf{D}}^{1/2}\boldsymbol{\beta})'(\widehat{\mathbf{D}}^{-1/2}\widehat{\mathbf{B}}\widehat{\mathbf{D}}^{-1/2})^{-1}(\widehat{\mathbf{D}}^{-1/2}\widehat{\mathbf{B}}\widehat{\mathbf{D}}^{1/2}\boldsymbol{\beta}).$$

Gathering up terms that do not depend on $\boldsymbol{\beta}$ and letting them equal Q, and substituting $\widehat{\mathbf{D}}^{-1/2}\mathbf{X}'\mathbf{X}\widehat{\mathbf{D}}^{-1/2} = \widehat{\mathbf{B}}$, $\mathbf{b} = \widehat{\mathbf{D}}^{-1}\mathbf{X}'\mathbf{y}$ and $\left(\widehat{\mathbf{D}}^{-1}\mathbf{X}'\mathbf{X}\widehat{\mathbf{D}}^{-1}\right)^{-1} = \widehat{\mathbf{D}}(\mathbf{X}'\mathbf{X})^{-1}\widehat{\mathbf{D}}$ then the difference is

$$
\begin{aligned}
&= Q + \frac{1}{\sigma_\varepsilon^2}\boldsymbol{\beta}'\mathbf{X}'\mathbf{y} - \frac{1}{\sigma_\varepsilon^2}(\widehat{\mathbf{D}}^{-1}\mathbf{X}'\mathbf{X}\boldsymbol{\beta})'\left(\widehat{\mathbf{D}}^{-1}\mathbf{X}'\mathbf{X}\widehat{\mathbf{D}}.^{-1}\right)^{-1}\mathbf{b} \\
&\quad - \frac{1}{2\sigma_\varepsilon^2}\boldsymbol{\beta}'\mathbf{X}'\mathbf{X}\boldsymbol{\beta} \ + \frac{1}{2\sigma_\varepsilon^2}(\widehat{\mathbf{D}}^{-1}\mathbf{X}'\mathbf{X}\boldsymbol{\beta})'\left(\widehat{\mathbf{D}}^{-1}\mathbf{X}'\mathbf{X}\widehat{\mathbf{D}}^{-1}\right)^{-1}(\widehat{\mathbf{D}}^{-1}\mathbf{X}'\mathbf{X}\boldsymbol{\beta}) \\
&= Q + \frac{1}{\sigma_\varepsilon^2}\boldsymbol{\beta}'\mathbf{X}'\mathbf{y} - \frac{1}{\sigma_\varepsilon^2}(\widehat{\mathbf{D}}^{-1}\mathbf{X}'\mathbf{X}\boldsymbol{\beta})'\widehat{\mathbf{D}}(\mathbf{X}'\mathbf{X})^{-1}\widehat{\mathbf{D}}\widehat{\mathbf{D}}^{-1}\mathbf{X}'\mathbf{y} \ + \\
&\quad - \frac{1}{2\sigma_\varepsilon^2}\boldsymbol{\beta}'\mathbf{X}'\mathbf{X}\boldsymbol{\beta} + \frac{1}{2\sigma_\varepsilon^2}(\widehat{\mathbf{D}}^{-1}\mathbf{X}'\mathbf{X}\boldsymbol{\beta})'\widehat{\mathbf{D}}(\mathbf{X}'\mathbf{X})^{-1}\widehat{\mathbf{D}}(\widehat{\mathbf{D}}^{-1}\mathbf{X}'\mathbf{X}\boldsymbol{\beta}) \\
&= Q.
\end{aligned}
$$

Given this the summary and individual data models will be equivalent up to a constant $Q = -\frac{n}{2}\log(2\pi\sigma_\varepsilon^2) + \frac{p}{2}\log(2\pi\sigma_\varepsilon^2) + \frac{1}{2}\log|\widehat{\mathbf{D}}^{-1/2}\widehat{\mathbf{B}}\widehat{\mathbf{D}}^{-1/2}|$, as the $-\frac{1}{2\sigma_\varepsilon^2}\mathbf{y}'\mathbf{y}$ and $\frac{1}{2\sigma_\varepsilon^2}\mathbf{b}'\left(\widehat{\mathbf{D}}^{-1/2}\widehat{\mathbf{B}}\widehat{\mathbf{D}}^{-1/2}\right)^{-1}\mathbf{b}$ terms cancel, that does not depend on $\boldsymbol{\beta}$. This assumes that σ_ε^2 is known and the full LD matrix is computed from the individual data genotypes. In reality we do not know σ_ε^2 but estimate it using posterior inference and the MCMC algorithm. The difference between the individual and summary likelihoods in dependent on σ_ε^2 with the deviation dependent on the difference between n and p.

6. Supplementary Note 5 - Additional acknowledgements

UKB: This study has been conducted using UK Biobank resource under Application Number 12514. UK Biobank was established by the Wellcome Trust medical charity, Medical Research Council, Department of Health, Scottish Government and the Northwest Regional Development Agency. It has also had funding from the Welsh Assembly Government, British Heart Foundation and Diabetes UK. **ARIC**: dbGaP accession: phs000090. The Atherosclerosis Risk in Communities Study is carried out as a collaborative study supported by National Heart, Lung, and Blood Institute contracts (HHSN268201100005C, HHSN268201100006C, HHSN268201100007C, HHSN268201100008C, HHSN268201100009C,

HHSN268201100010C, HHSN268201100011C, and HHSN268201100012C), R01HL087641, R01HL59367 and R01HL086694; National HumanGenome Research Institute contract U01HG004402; and National Institutes of Health contract HHSN268200625226C. The authors thank the staff and participants of the ARIC study for their important contributions. Infrastructure was partly supported by Grant Number UL1RR025005, a component of the National Institutes of Health and NIH Roadmap for Medical Research. **NHS & HPFS (GENEVA)**: dbGaP accessions phs000091. Funding support for the GWAS of Gene and Environment Initiatives in Type 2 Diabetes was provided through the NIH Genes, Environment and Health Initiative [GEI] (U01HG004399). The human subjects participating in the GWAS derive from The Nurses' Health Study (NHS) and Health Professionals' Follow-up Study (HPFS) and these studies are supported by National Institutes of Health grants CA87969, CA55075, and DK58845. Assistance with phenotype harmonization and genotype cleaning, as well as with general study coordination, was provided by the Gene Environment Association Studies, GENEVA Coordinating Center (U01 HG004446). Assistance with data cleaning was provided by the National Center for Biotechnology Information. Funding support for genotyping, which was performed at the Broad Institute of MIT and Harvard, was provided by the NIH GEI (U01HG004424).**UK10K**: The UK10K project was funded by the Wellcome Trust award WT091310. Twins UK (TUK): TUK was funded by the Wellcome Trust and ENGAGE project grant agreement HEALTH-F4-2007-201413. The study also receives support from the Department of Health via the National Institute for Health Research (NIHR)-funded BioResource, Clinical Research Facility and Biomedical Research Centre based at Guy's and St. Thomas' NHS Foundation Trust in partnership with King's College London. Dr Spector is an NIHR senior Investigator and ERC Senior Researcher. Funding for the project was also provided by the British Heart Foundation grant PG/12/38/29615 (Dr Jamshidi). A full list of the investigators who contributed to the UK10K sequencing is available from www.UK10K.org. **HRS**: dbGaP accession phs000428.v1.p1. HRS is supported by the National Institute on Aging (NIA U01AG009740). The genotyping was funded separately by the National

Institute on Aging (RC2 AG036495, RC4 AG039029). Genotyping was conducted by the NIH Center for Inherited Disease Research (CIDR) at Johns Hopkins University. Genotyping quality control and final preparation of the data were performed by the Genetics Coordinating Center at the University of Washington. **ESTB**: The Estonian Genome Centre of University of Tartu Study was supported by EU Horizon 2020 grants 692145, 676550, and 654248; Estonian Research Council Grant IUT20-60, NIASC, EIT Health; NIH BMI grant 2R01DK075787-06A1; and the European Regional Development Fund (project 2014-2020.4.01.15-0012 GENTRANSMED). Estonian Biobank data were accessed via request from the Estonian Genome Center through data release procedures described at https://www.geenivaramu.ee/en/biobank.ee/data-access.

Supplementary References

[1] Zhu, X., Stephens, M. *et al.* Bayesian large-scale multiple regression with summary statistics from genome-wide association studies. *The Annals of Applied Statistics* **11**, 1561–1592 (2017).

[2] Yengo, L. *et al.* Meta-analysis of genome-wide association studies for height and body mass index in ≈700000 individuals of european ancestry. *Human Molecular Genetics* **27**, 3641–3649 (2018).

[3] ARIC Investigators. The atherosclerosis risk in community (aric) Study: Design and objectives. *American Journal of Epidemiology* **129**, 687–702 (1989).

[4] 1000 Genomes Project Consortium *et al.* A global reference for human genetic variation. *Nature* **526**, 68 (2015).

[5] UK10K consortium. The UK10K project identifies rare variants in health and disease. *Nature* **526**, 82 (2015).

[6] Moser, G. *et al.* Simultaneous discovery, estimation and prediction analysis of complex traits using a bayesian mixture model. *PLoS Genetics* **11**, e1004969 (2015).

[7] Wen, X. & Stephens, M. Using linear predictors to impute allele frequencies from summary or pooled genotype data. *The Annals of Applied Statistics* **4**, 1158 (2010).

[8] Chang, C. C. *et al.* Second-generation PLINK: rising to the challenge of larger and richer datasets. *Gigascience* **4**, 7 (2015).

[9] Vilhjálmsson, B. J. *et al.* Modeling linkage disequilibrium increases accuracy of polygenic risk scores. *The American Journal of Human Genetics* **97**, 576–592 (2015).

[10] Robinson, M. R. *et al.* Genetic evidence of assortative mating in humans. *Nature Human Behaviour* **1**, 0016 (2017).

[11] Haseman, J. & Elston, R. The investigation of linkage between a quantitative trait and a marker locus. *Behavior Genetics* **2**, 3–19 (1972).

[12] Yang, J., Lee, S. H., Goddard, M. E. & Visscher, P. M. GCTA: a tool for genome-wide

complex trait analysis. *The American Journal of Human Genetics* **88**, 76–82 (2011).

[13] Yang, J., Zeng, J., Goddard, M. E., Wray, N. R. & Visscher, P. M. Concepts, estimation and interpretation of snp-based heritability. *Nature Genetics* **49**, 1304 (2017).

[14] Meuwissen, T. H. E., Hayes, B. J. & Goddard, M. E. Prediction of total genetic value using genome-wide dense marker maps. *Genetics* **157**, 1819–1829 (2001).

[15] Habier, D., Fernando, R. L., Kizilkaya, K. & Garrick, D. J. Extension of the Bayesian alphabet for genomic selection. *BMC Bioinformatics* **12**, 186 (2011).

[16] Erbe, M. *et al.* Improving accuracy of genomic predictions within and between dairy cattle breeds with imputed high-density single nucleotide polymorphism panels. *Journal of Dairy Science* **95**, 4114–4129 (2012).

[17] Zhou, X., Carbonetto, P. & Stephens, M. Polygenic modeling with Bayesian sparse linear mixed models. *PLoS Genetics* **9**, e1003264 (2013).

[18] Zeng, J. *et al.* Signatures of negative selection in the genetic architecture of human complex traits. *Nature genetics* **50**, 746 (2018).

[19] Fernando, R. L. & Garrick, D. Bayesian methods applied to GWAS. *Genome-Wide Association Studies and Genomic Prediction* 237–274 (2013).

[20] Henderson, C. R. Estimation of genetic parameters. *Annals of Mathematical Statistics* **21**, 309–310 (1950).

[21] Yang, J. *et al.* Common SNPs explain a large proportion of the heritability for human height. *Nature Genetics* **42**, 565–569 (2010).

[22] Mak, T. S. H., Porsch, R. M., Choi, S. W., Zhou, X. & Sham, P. C. Polygenic scores via penalized regression on summary statistics. *Genetic Epidemiology* **41**, 469–480 (2017).

[23] Yang, J. *et al.* Conditional and joint multiple-SNP analysis of GWAS summary statistics identifies additional variants influencing complex traits. *Nature Genetics* **44**, 369–375 (2012).

[24] Fernando, R. L., Habier, D., Stricker, C., Dekkers, J. C. M. & Totir, L. R. Genomic selection. *Acta Agriculturae Scandinavica, Section A — Animal Science* **57**, 192–195 (2007). URL https://doi.org/10.1080/09064700801959395. https://doi.org/10.1080/

09064700801959395.
