# Supplementary material for: Improved polygenic prediction by Bayesian multiple regression on summary statistics
Source: Nat Commun. 2019 Nov 8;10:5086. doi: 10.1038/s41467-019-12653-0 (PMC6841727; doi:10.1038/s41467-019-12653-0)
Supplement: Supplementary file 2 — Reporting Summary [file 41467_2019_12653_MOESM2_ESM.pdf]

nature research

Corresponding author(s): Luke Lloyd-Jones, Jian Zeng, Jian Yang, Peter Visscher

Last updated by author(s): 27/8/19

Reporting Summary

Nature Research wishes to improve the reproducibility of the work that we publish. This form provides structure for consistency and transparency in reporting. For further information on Nature Research policies, see Authors & Referees and the Editorial Policy Checklist.

Statistics

For all statistical analyses, confirm that the following items are present in the figure legend, table legend, main text, or Methods section.

n/a	Confirmed	
☐	☒	The exact sample size (*n*) for each experimental group/condition, given as a discrete number and unit of measurement
☒	☐	A statement on whether measurements were taken from distinct samples or whether the same sample was measured repeatedly
☐	☒	The statistical test(s) used AND whether they are one- or two-sided<br>*Only common tests should be described solely by name; describe more complex techniques in the Methods section.*
☐	☒	A description of all covariates tested
☐	☒	A description of any assumptions or corrections, such as tests of normality and adjustment for multiple comparisons
☐	☒	A full description of the statistical parameters including central tendency (e.g. means) or other basic estimates (e.g. regression coefficient) AND variation (e.g. standard deviation) or associated estimates of uncertainty (e.g. confidence intervals)
☐	☒	For null hypothesis testing, the test statistic (e.g. *F*, *t*, *r*) with confidence intervals, effect sizes, degrees of freedom and *P* value noted<br>*Give P values as exact values whenever suitable.*
☐	☒	For Bayesian analysis, information on the choice of priors and Markov chain Monte Carlo settings
☐	☒	For hierarchical and complex designs, identification of the appropriate level for tests and full reporting of outcomes
☐	☒	Estimates of effect sizes (e.g. Cohen's *d*, Pearson's *r*), indicating how they were calculated

Our web collection on statistics for biologists contains articles on many of the points above.

Software and code

Policy information about availability of computer code

Data collection

UK Biobank data were downloaded from repository under application number 12514. Quality control was performed using the PLINK v1.90b3.41 software. Ancestry assignment and relatedness assignment was performed using principal component projection in GCTA (v1.90.0beta). Principal components used for covariate adjustment for summary statistics generation were calculated using flashPCA version 2. Other data set QC had been previously performed in other work with the QC parameters described in the Materials and Methods data section. PLINK v1.90b3.41 and GCTA (v1.90.0beta) were the primary software used to process these data.

Data analysis

A custom implementation of the methods described in the manuscript was performed in the GCTB (version 2) software available at http://cnsgenomics.com/software/gctb/#Overview and at source code at GitHub (https://github.com/jianzeng/GCTB). Methods used for prediction comparison include: individual data BayesR in software bayesRv2 (https://github.com/syntheke/bayesR); LDpred download and installed from https://github.com/bvilhjal/ldpred; SBLUP performed in GCTA software (version 1.91.4 beta3); clumping and variant thresholding performed in PLINK v1.90b3.41; Regression with Summary Statistics downloaded and installed from https://github.com/stephenslab/rss. For heritability comparison the following: LD score regression downloaded and installed from https://github.com/bulik/ldsc and HE regression performed in GCTA software (version 1.91.4 beta3). Processing of results and figure generation was performed using the R programming language.

For manuscripts utilizing custom algorithms or software that are central to the research but not yet described in published literature, software must be made available to editors/reviewers. We strongly encourage code deposition in a community repository (e.g. GitHub). See the Nature Research guidelines for submitting code & software for further information.

Data

Policy information about availability of data

All manuscripts must include a data availability statement. This statement should provide the following information, where applicable:

- Accession codes, unique identifiers, or web links for publicly available datasets
- A list of figures that have associated raw data
- A description of any restrictions on data availability

This study makes use of data from dbGaP ARIC, HRS and (accessions: phs000090, phs000091 and phs000674.v2.p2)), UK10K project (EGA accessions: EGAS00001000108 and EGAS00001000090), and UK Biobank Resource (application number: 12514). Estonian Biobank data were accessed via request from the Estonian Genome Center through data release procedures described at https://www.geenivaramu.ee/en/biobank.ee/data-access.

Field-specific reporting

Please select the one below that is the best fit for your research. If you are not sure, read the appropriate sections before making your selection.

☒ Life sciences ☐ Behavioural & social sciences ☐ Ecological, evolutionary & environmental sciences

For a reference copy of the document with all sections, see nature.com/documents/nr-reporting-summary-flat.pdf

Life sciences study design

All studies must disclose on these points even when the disclosure is negative.

Sample size

For the simulation studies a random subset of 100,000 individuals from the 348,580 unrelated European ancestry individuals from the UK Biobank were chosen. The simulation studies were designed to be a within data comparison between the new proposed method and existing methods. From experience this sample size is sufficient (more than) to produce accurate predictors. For the cross-validation analyses the full set of 348,580 unrelated European ancestry individuals from the UKB were used for summary statistics generation. This sample size was chosen to observe the maximum prediction accuracy that could be achieved using unrelated individuals in the UKB data set. In the across Biobank prediction analyses the full set of 456,426 UKB European ancestry individuals from the UKB were used. For out of sample prediction, the HRS data set consisted of 8,552 unrelated individuals, which was the maximum number available. For the Estonian Biobank 32,594 individuals genotyped on the Global Screening Array were used, which was the maximum available at the time of analysis.

Data exclusions

The UKB data contains genotypes for 488,377 individuals (including related individuals) that passed sample quality control (99.9\% of total samples). A subset of 456,426 European ancestry individuals was selected using the protocol described in Yengo et al. 2018. To exclude related individuals, a genomic relationship matrix (GRM) was constructed
with 1,123,943 HapMap3 variants further filtered for minor allele frequency (MAF) > 0.01, pHWE < 10^-6 and missingness < 0.05 in the European subset, resulting in a final set of 348,580 unrelated (absolute GRM off-diagonal < 0.05) Europeans. Variant quality control included: removal of multi-allelic variants, SNPs with imputation info score < 0.3, retained SNPs with hard-call genotypes with > 0.9 probability, removed variants with minor allele count (MAC) < 5, Hardy-Weinberg p-value (pHWE) < 10^{-5} and removed variants with missingness > 0.05, which resulted in 46,500,935 SNPs for the 456,426 individuals.

The ARIC+GENEVA data consisted of 12,942 unrelated individuals determined by an absolute GRM off-diagonal relatedness cutoff of < 0.05. After imputation to the
Phase 3 of the 1000 Genomes Project (1000G), 1,182,558 HM3 SNPs (MAF > 0.01) were selected and available for analysis after quality control.
Whole-genome sequencing data from the 1000G project was used for LD matrix reference calculation. These data were subsetted to a set of 397 individuals with
European ancestry to be consistent with the LD reference used in Zhu and Stephens 2017. Whole-genome sequencing data from the UK10K project was also used for analysis. The UK10K contains 17.6 million genetic variants (excluding singletons and doubletons) in 3,642 unrelated individuals after quality control,
which was performed as per Yang et al. 2015.

We used genotypes imputed to the 1000G reference panel and phenotypes from 8,552 unrelated (absolute GRM off-diagonal < 0.05) participants of the Health and Retirement Study (HRS). After imputation and restricting variants with an imputation quality score >0.3, MAF >0.01 and a pHWE >10^{-6} there were 24,777,992 SNPs available for prediction. The Estonian Biobank is a cohort study of over 50,000 individuals over 18 years of age with phenotypic and genotypic data. For the prediction analysis we used data from 32,594 individuals genotyped on the Global Screening Array. These data were imputed to the Estonian reference \citep{mitt2017improved}, created from the whole genome sequence data of 2,244 participants. Markers with imputation quality score > 0.3 were selected leaving a total of 11,130,313 SNPs for prediction.

For simulation and cross validation, the 1,094,841 variant subset was formed from the 1,365,446 HM3 SNPs further filtered on
MAF>0.01, strand ambiguous SNPs, removal of long-range LD regions (defined in Bycroft et al. 2018
Table S13 and includes the MHC), which increased model stability across a large set of phenotypes, and overlapped with the 1000G genetic map downloaded from \url{joepickrell/1000-genomes-genetic-maps}. The 1000G genetic map is required for use in the LD matrix shrinkage estimator. To investigate the capacity of SBayesR to perform analyses at large
scale, we generated a pruned set (R2>0.99) of 2,865,810 common (MAF > 0.01) variants
that were of good quality in the UKB, overlapped with previous large scale GWAS and
were present in the 1000G genetic map

For across biobank prediction, we subsetted the set of 1,094,841 HM3 variants
to 982,074 variants that overlapped with those in both the BMI and height summary statistics sets. To improve method convergence we removed variants from the Yengo \emph{et al.}\citep{yengo_height} summary statistics To improve method convergence we removed variants from the Yengo \emph{et al.}\citep{yengo_height} summary statistics
that had a per variant sample size that deviated substantially from the mean of the sample size distribution over
all variants, which was also performed by Pickrell et al. 2014 and recommended by Zhu and Stephens 2017. To minimise the variants removed, we
interrogated the distributions of per variant sample size in each of the BMI and height summary statistics sets and removed variants in the lower 2.5th percentile and upper 5th percentile of the per variant sample size distribution for BMI and in the lower 5th percentile for height (Figure S6).
This left 932,969 and 909,293 variants with summary information for height and BMI respectively. These sets of variants were also used in the LDpred and RSS analyses.

Replication

The major experimental findings include the validation of the the newly proposed method to be more accurate at polygenic prediction at a much smaller computational cost. The breadth of scenarios and real data analyses are sufficient, we believe, evidence for reviewers to assess these conclusions.

Randomization

For each of the genome-wide association studies age, sex and 10 principal components were adjusted for. These covariates are standard in these types of genetic analyses.

Blinding

Blinding in population data collection is not concern as no treatment is being investigated.

Reporting for specific materials, systems and methods

We require information from authors about some types of materials, experimental systems and methods used in many studies. Here, indicate whether each material, system or method listed is relevant to your study. If you are not sure if a list item applies to your research, read the appropriate section before selecting a response.

Materials & experimental systems

n/a	Involved in the study	
☒	☐	Antibodies
☒	☐	Eukaryotic cell lines
☒	☐	Palaeontology
☒	☐	Animals and other organisms
☐	☒	Human research participants
☒	☐	Clinical data

Methods

n/a	Involved in the study	
☒	☐	ChIP-seq
☒	☐	Flow cytometry
☒	☐	MRI-based neuroimaging

Human research participants

Policy information about studies involving human research participants

Population characteristics

All data we access from previous studies that detail the population characteristics. The data and the agreement numbers have been acknowledged in the manuscript.

Recruitment

All data we access from previous studies that detail the data recruitment. The data and the agreement numbers have been acknowledged in the manuscript.

Ethics oversight

All data we access from previous studies that detail the ethics oversight, which is mentioned in detail in the data methods section. The data and the agreement numbers have been acknowledged in the manuscript.

Note that full information on the approval of the study protocol must also be provided in the manuscript.
